# The Discovery of phiAGATE, A Novel Phage Infecting *Bacillus pumilus*, Leads to New Insights into the Phylogeny of the Subfamily *Spounavirinae*

**DOI:** 10.1371/journal.pone.0086632

**Published:** 2014-01-23

**Authors:** Jakub Barylski, Grzegorz Nowicki, Anna Goździcka-Józefiak

**Affiliations:** Department of Molecular Virology, Faculty of Biology, Adam Mickiewicz University, Poznań, Poland; Institute of Immunology and Experimental Therapy, Polish Academy of Sciences, Poland

## Abstract

The *Bacillus* phage phiAGATE is a novel myovirus isolated from the waters of Lake Góreckie (a eutrophic lake in western Poland). The bacteriophage infects *Bacillus pumilus*, a bacterium commonly observed in the mentioned reservoir. Analysis of the phiAGATE genome (149844 base pairs) resulted in 204 predicted protein-coding sequences (CDSs), of which 53 could be functionally annotated. Further investigation revealed that the bacteriophage is a member of a previously undescribed cluster of phages (for the purposes of this study we refer to it as “Bastille group”) within the *Spounavirinae* subfamily. Here we demonstrate that these viruses constitute a distinct branch of the *Spounavirinae* phylogenetic tree, with limited similarity to phages from the *Twortlikevirus* and *Spounalikevirus* genera. The classification of phages from the Bastille group into any currently accepted genus proved extremely difficult, prompting concerns about the validity of the present taxonomic arrangement of the subfamily.

## Introduction

*Spounavirinae* is a subfamily of Myoviruses with large (75–100 nm diameter) isometric heads and long tails (140–220 nm) [1]. All known members of the taxon infect bacteria from the phylum *Firmicutes* and are strictly virulent [1], [2]. Since phages incapable of lysogeny make good candidates for phage therapy, there is increasing interest in this subfamily [3], [4].

Spounaviruses possess large (127–157 bp), linear dsDNA genomes ending with long terminal repeats (LTRs). This can cause problems during assembly of reads from shotgun sequencing methods, since the resulting sequences appear circular despite the linear arrangement of the genome [5], [6]. Genes of these bacteriophages have a modular arrangement, with distinguishable DNA packaging, head and tail morphogenesis and DNA replication modules. Viruses from the subfamily frequently carry inteins within immature polypeptides or introns in transcripts and have their own, unique sets of tRNAs [3], [7]–[11].

The taxon comprises two genera: *Spounalikevirus* (termed “SPO1-like viruses” prior to the 2012 Virus Taxonomy release) and *Twortlikevirus*. The genus *Spounalikevirus* currently includes only one ICTV (International Committee on Taxonomy of Viruses) recognized member, *Bacillus* phage SPO1, while *Twortlikevirus* contains the staphylococcal phages (phages Twort, G1, and K), as well as viruses infecting *Listeria* (phages A100 and a A510). Together with orphan species (*Enterococcus* phage phiEC24C and *Lactobacillus* phage LP65), there are eight ICTV-accepted subfamily members [12], [13]. Nevertheless, the NCBI Taxonomy database lists 25 different phages (9 Spounalikeviruses, 9 Twortlikeviruses, and 7 unclassified phages), most with complete genomes available in GenBank [14].

The aim of this study was to characterize the *Bacillus* phage phiAGATE, a large spounavirus isolated from the waters of a stratified, eutrophic lake (Lake Góreckie in western Poland). phiAGATE infects *Bacillus pumilus,* a Gram-positive (or variable), motile, spore-forming bacterium related to *B. subtilis* and *B. lichenoformis. B. pumilus* is also often found in soil, food products and water (including water from studied lake, Barylski et al. unpublished data) [14], [15]. Some strains have been isolated from the mid-gut of black tiger shrimp, emperor moth caterpillars, feathers, leather, paper, and even on the surfaces in the International Space Station or clean-rooms of the spacecraft assembly facility [15]–[17].

*B. pumilus* is a nitrogen-fixer, capable of metabolic transformation of molecular nitrogen into ammonia [18], [19]. It also solubilizes and mineralizes otherwise insoluble phosphorus compounds [20], [21] and promotes the growth of some crops [22], [23]. (however, at least one known strain is pathogenic to mango plants [24]).

Several strains are used in industry (as a source of alkaline protease utilized for hide dehairing [25], [26], or a xylanase used in papermaking [27], [28]). Other synthesize a bacteriocin active against many Gram-positive bacteria (including Methicillin-resistant *Staphylococcus aureus*) [29]. Certain marine isolates possess a quorum-sensing interference mechanism [30], [31]. Although the species is generally considered to be non-pathogenic to humans, a few cases of cutaneous lesions and food poisoning were associated with this bacterium [32], [33].

At least one environmental isolate has been shown to carry active prophages within its genetic material [34]. However, few phages are known to infect *B. pumilus* and there are almost no associated genomic data (see Table 1). We therefore sought to comprehensively characterize phiAGATE and learn more about its structure, genomics and phylogeny.

**Table 1:** Phages infecting *Bacillus pumilis*.

Family	Phage	Genome	Comments/source
*Myoviridae*	PMB1	n.a.	sporulation-converting [51]
*Myoviridae*	PMJ1	n.a.	sporulation-converting [51]
*Myoviridae*	702phi1-1	14 partial sequences (8.9 kb)	(Hardies and Serwer, unpublished data)
*Siphoviridae*	PBP1	n.a.	[62]
*Podoviridae*	Φ29	NC_011048.1 (19.2 kb)	isolated as *Bacillus subtilis* phage, also infects *B amyloliquefaciens* and *B. licheniformis* [63]
n.a.	31	n.a.	Available at JSCC [64]
n.a.	NP-5	n.a.	Available at JSCC [64]
n.a.	236	n.a.	Available at ATCC (Gordon RE, unpublished data)

(JSCC - Japan Society for Culture Collections, ATCC - American Type Culture Collection).

## Materials and Methods

### Ethics Statement

All samples from protected areas were provided by the Department of Hydrobiology, Adam Mickiewicz University, Poznań, Poland (hydro@amu.edu.pl). An appropriate permit (number 6/2011-09/2009) was obtained from the Wielkopolski National Park Administration (Jeziory, 62–050 Mosina, Poland, sekretariat@wielkopolskipn.pl) for this study.

### Bacterial Strains

The host organism, *Bacillus pumilus* strain GL1, had been isolated from sediments of Lake Góreckie (52°15′46″N 16°47′53″E, Greater Poland region, western Poland) in previous studies. 16S rDNA (using primers16S_fD1 [39], 16S_pA [40], 16S_1100R, 16S_1100F, 16S_519RDeg and 16_S357F [41]) was sequenced and compared to the NCBI Nucleotide collection database (nr/nt) to determine species identity of this microorganism [14]. The obtained sequence was deposited in GenBank database, under accession number KC412012. Identification was confirmed using Microbact 24E tests (Oxoid, procedure modified for *Bacillus* sp. according to Logan and Berkeley [42]) and peptide profiling of the total proteome using LC-ESI-MS/MS spectrometry (in the Laboratory of Mass Spectrometry, IBB PAS) [43].

### Growth Media

Water Plate Count Agar (CM1012, Oxoid) and LB broth were used for bacteriophage isolation. Routine phage cultures were prepared either in LB-MM medium (LB broth supplemented with 0.2% maltose and 10 mM MgSO_4_) or on LB agar.

### Bacteriophage Isolation

Water samples were collected between April and August 2010 in the littoral and pelagic zones of Lake Góreckie. An enrichment culture strategy was used to multiply phages prior to detection. Each sample (4.5 ml) was incubated overnight with 0.5 ml of 10×LB broth and 0.5 ml of candidate host culture (previously grown 18 h in LB broth medium). The resulting mixture was filtered through a 0.22 µm sterile Millex-GP filter unit (Millipore) and a drop (1–2 µl) of the filtrate was spotted on the freshly prepared bacterial lawn. The lawn was allowed to grow overnight. If a clearance zone was observed, the remaining filtrate was titred and single plaques cut out to be used as an inoculum for further steps.

### Phage Growth

Host cultures were allowed to grow for 18 h at 30°C, inoculated with phage suspension to reach the titer of ∼5×10^7^ PFU/ml (or, in the case of initial cultures with material isolated from a single plaque), and incubated overnight. Crude phage lysates were filtered through a 0.22 µm sterile Millex-GP filter unit, titred, and stored at 4°C.

### Transmission Electron Microscopy (TEM)

Phage particles were separated from the bacteria-free lysates (10 ml) by filtering through a 0.015 µm Nuclepore Track-Etched Membrane (Whatman) [44], washed with SM buffer without NaCl and gelatin (8 mM MgSO4, 50 mM Tris-HCl pH 7.5) and resuspended in the same buffer. The resulting suspension was applied to a Formvar/Carbon-coated copper EM grids and the phage particles were allowed to absorb for 45 s after which the grids were washed with sterile deionized water. After negative staining with 2% uranyl acetate they were air-dried and studied using a JEOL JEM-1400 transmission electron microscope at 120 kV. The size of the head and length of the tail were calculated from 12 independent measurements of separate virions and reported as a mean values ± standard deviation.

### Phage Adsorption and Replication Characteristics

Both adsorption rate and one-step growth curves were determined as described by Sillankorva et al. [45], with minor modifications (mean values from four independent replicates are presented).

For the adsorption experiment, the bacteria in the steady-state growth phase were diluted in LB-MM broth to an optical density OD_600_ of 0.6 (∼3×10^8^ CFU/ml). 30 ml of the bacterial suspension and 30 µl of the appropriately diluted phage solution were mixed in order to obtain a multiplicity of infection (MOI) of 0.01 and the resulting mixture was incubated at 30°C with shaking (230 rpm). Samples (1 ml) were collected every minute over a period of 10 min, immediately treated with chloroform (1% v/v), diluted, mixed with 3 ml of soft agar (LB broth with 0.7% low gelling temperature agarose), and plated on LB agar plates. After overnight incubation at 30°C, plaques were counted and the adsorption rate was calculated according to Barry and Goebel [46].

To determine the dynamics of phage growth, 10 ml of an overnight host culture was harvested by centrifugation (4000×g, 12 min, 21°C), resuspended in 30 ml of fresh LB-MM medium, and incubated (30°C, 230 rpm) until the suspension reached an OD_600_ of 0.6. The phage solution (3 µl) was then added to obtain a MOI of 0.001, and phages were allowed to adsorb for 10 min at 30°C. The mixture was centrifuged (4000×g, 12 min, 21°C) and the pellet resuspended in 30 ml of fresh LB-MM medium and incubated at 30°C. Two samples (0.5 ml) were taken every 10 min over a period of 80 min. One was mixed with 3 ml of soft agar and plated immediately, while the other was plated after treatment with 1% (v/v) chloroform to release intracellular phages.

### Purification and Sequencing of viral DNA

20 ml of cleared lysate were treated with DNase I (1,5 Kunitz units per ml of suspension, 37°C, 30 min) to remove the remains of unprotected host genetic material. Phage particles were concentrated by precipitation with PEG-8000 solution (5 ml of 20% PEG-8000 in 2.5 M NaCl) followed by centrifugation (35000 rpm, 30 min, 4°C, rotor 55.2 Ti Beckman). DNA was isolated from the resulting pellet using a QIAamp DNA Mini Kit (Qiagen, manufacturer’s instructions, protocol D). After assessment of the quality by electrophoresis, DNA samples were either stored at −20°C or repurified, if needed (using the same kit, protocol L).

454 sequencing was performed at Genomed Inc. (Warsaw, Poland) as one single-ended run of a Genome Sequencer Junior (Roche) and assembled using GS De Novo Assembler 9 with the average coverage of 52.2× distributed among 16492 reads (leaving no gaps to be filled by the Sanger method).

### Analysis of the Phage Genome

Coding sequences (CDSs) were predicted using Genemark.hmm 2.8 (http://exon.gatech.edu/gmhmm2_prok.cgi) [47], Glimmer 3 [48], fgenesV0 (http://linux1.softberry.com) [49], and RAST 4.0 (http://rast.nmpdr.org/) [50]. Only the CDSs predicted by at least three tools were selected for further analysis. Domains contained in predicted proteins were detected with the InterProScan tool from the Geneious 5.6.6 software suite [51], [52]. Functional annotation was carried out by comparison of the results of the RAST analysis and a BLASTp (http://blast.ncbi.nlm.nih.gov/) search (against the non-redundant protein database) [53] with the detected domains. The quality of predictions was assessed using PFP, EFG online tools (http://kiharalab.org/) [54], [55] and Blast2GO (http://www.blast2go.com/) [56]. The latter program was also used to assign CDSs to ontological categories. The search for tRNA genes was performed with tRNAscan-SE 1.21 (http://lowelab.ucsc.edu/tRNAscan-SE/) [57], while tandem repeats were located by the Phobos Tandem Repeat Finder plug-in in Geneious [52], [58]. All stages of annotation were curated manually.

### Phylogenetic Analyses

To determine the phylogenetic position of the investigated virus, we chose to study six marker sequences representing different genomic modules: the major capsid protein and portal protein (head morphogenesis module), tail sheath protein (tail morphogenesis module), DNA polymerase and helicase (DNA replication module), and the large terminase subunit (product of the only functionally annotated gene in phiAGATE in the packaging module). With the exception of the helicase, we used proteins often described as taxonomic markers in the literature concerning phylogeny of myoviruses [1], [59]–[63]. The sequences from phiAGATE were predicted as described above, while their closest homologues from other phages were retrieved from GenBank using BLASTp and scanned for the relevant domains using the InterProScan tool from the Geneious suite (for details see Table S1).

To choose preliminary candidate taxa for the studied phages we performed BLASTp analysis (querying the non-redundant protein database [14]) of selected markers. Preliminary classification was confirmed by cluster analysis of the phages and 202 myoviruses with complete genomes deposited in the NCBI Reference Sequence database (RefSeq) [14], based on the similarity of the genomes. This analysis was conducted using the CLuster ANalysis of Sequences (CLANS) software package [64] which performs all-against-all BLAST searches, calculates attraction values from *P* values of high scoring segment pairs (HSPs), and visualizes the resulting similarity network using a variant of the Fruchterman–Reingold graph layout algorithm. Drulis-Kawa et al. previously applied a similar approach to Podoviruses [65], however, while they used tBLASTx algorithm to compare sequences, we found that this approach generated too much background noise to obtain meaningful clustering; we therefore used BLASTn (word size 7, other settings default). A similar analysis was also performed at the subfamily level to group the analyzed phages with 21 sequenced and classified (according to the NCBI taxonomy database) taxon members [7]–[11], [66]–[74]. We also compared the whole genomes of these phages using Gegenees 2.0.0 (tBLASTx method, fragment size –50, step size –25) [75] and generated dendrograms with SplitsTree 4.13.1 [76], using the neighbor joining method (as in [75] and [77]) based on the resulting similarity matrix.

To further explore in-subfamily relationships we prepared maximum likelihood (ML) trees for each protein marker, as well as the condensed tree (generated from concentrated alignments of all marker sequences), using PhyML 3.0 [78] (BEST topology search, 250 bootstrap replicates). The sequences were aligned using the MUSCLE alignment tool in Geneious [79] (with max. 1000 iterations), while evolution models were selected using ProtTest 3.2.1 [80]. All resulting trees were visualized in the Geneious tree viewer. Their congruence was assessed using the Online Calculation of Congruency Index (I_cong_, http://max2.ese.u-psud.fr/bases/upresa/pages/devienne/) based on the maximum agreement subtrees (MAST) method [81].

### Comparative Genomics

We used the BLAST Ring Image Generator (BRIG, with the tBLASTx as a comparison algorithm) [82] to generate circular maps of genomic similarity and the Progressive Mauve tool from the Geneious software suite to perform linear comparison [83]. Default parameters and settings were used for all tools unless stated otherwise. For detailed information about all the analyzed sequences see Table S2 (supporting information).

## Results

### Bacteriophage Isolation and Morphology

The first isolate of the novel bacteriophage (later named phiAGATE) was found in April 2010 in water samples collected above the sediment in the littoral zone of Lake Góreckie (52°15′46″N 16°47′53″E, Greater Poland region, western Poland).

**Figure 1:**
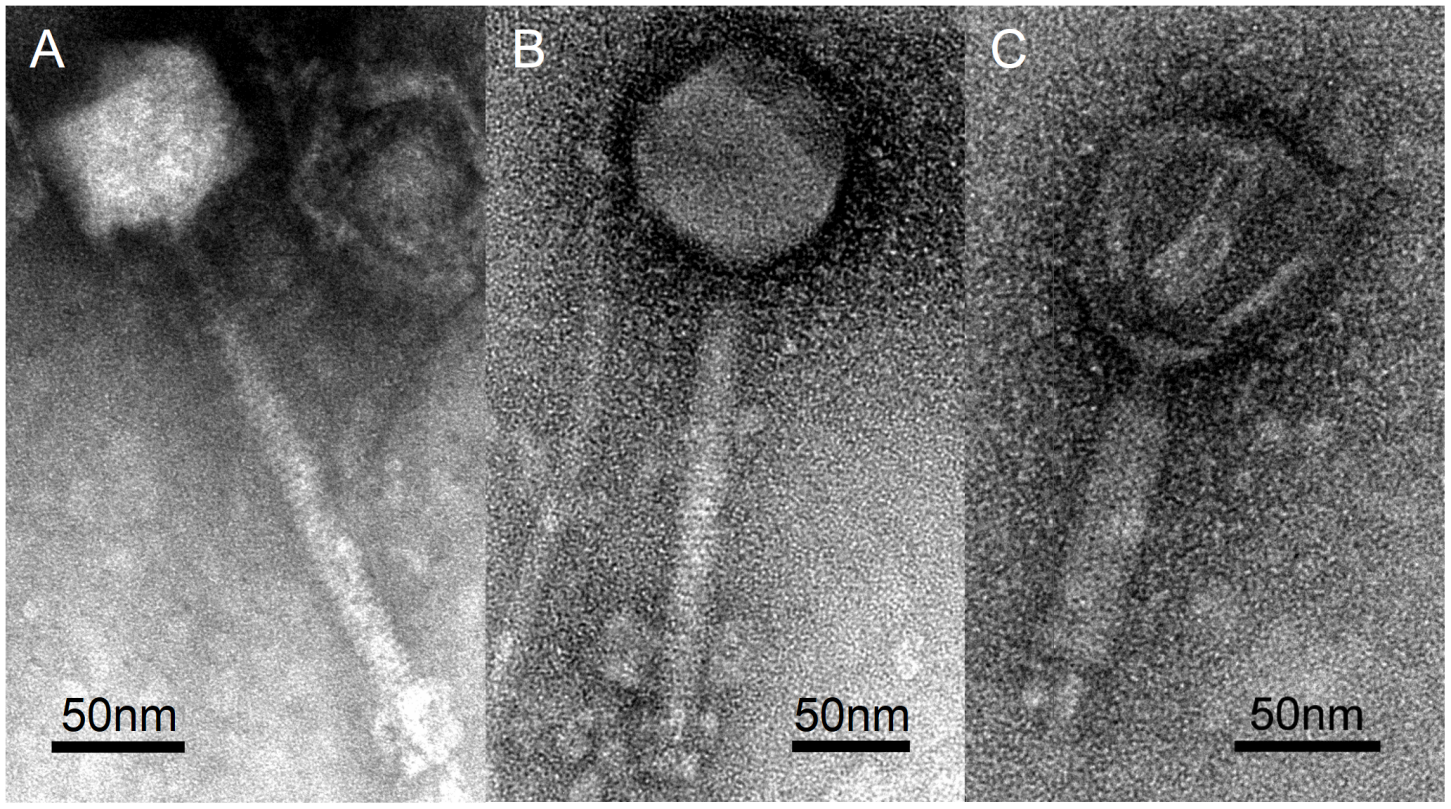
Transmission electron micrographs of phiAGATE virions. Panels A and B show typical morphologies observed under the electron microscope. Panel C depicts a phage particle with contracted tail.

TEM analysis revealed that virions of phiAGATE display typical binary symmetry characteristic of the order *Caudovirales*. The head is roughly icosahedral, with diameter of 91.16 (±3.71) nm. The length of the tail is 165.41 (±8.67) nm, however, some virions had altered tail morphology that clearly suggested a contraction. We therefore assumed that the phage is a member of *Myoviridae* family (Figure 1).

### Phage Adsorption and Replication Characteristics

The adsorption process turned out to be very rapid, with the titer dropping below 10% within two minutes of the start of the experiment. The adsorption rate constant calculated for this period was 6.44×10^−9 ^ml/min.

**Figure 2:**
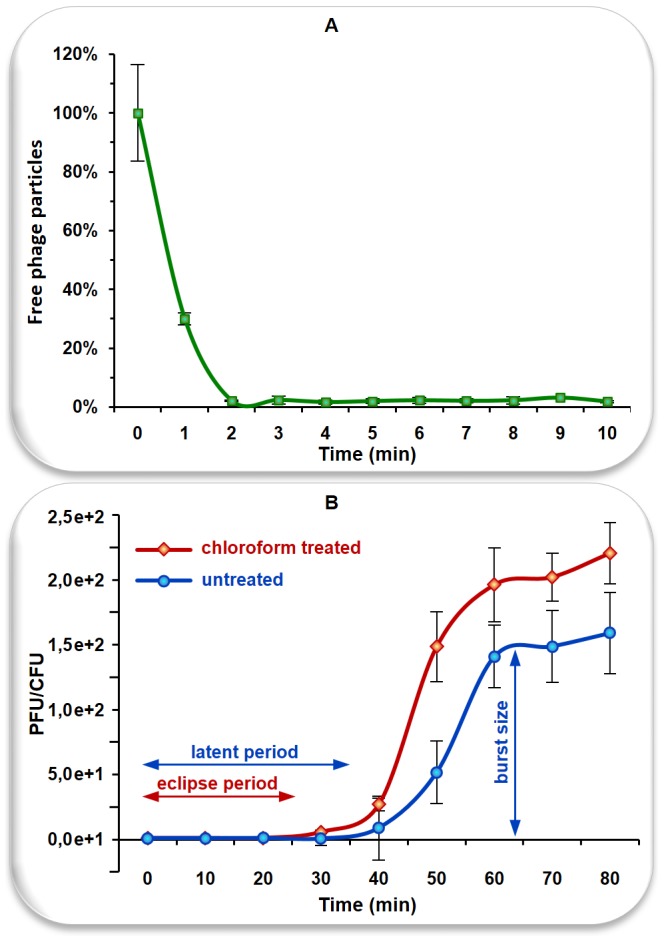
Replication and adsorption dynamics of phiAGATE. Panel A shows an adsorption curve and panel B one-step growth curve. Error bars represent standard deviation.

The lengths of eclipse and latent periods inferred from the results of the one-step growth experiment were ∼25 and ∼35 min, respectively. The phage reached a burst size of 153 PFU per infected cell during the first 60–65 min of the experiment (for details, see Figure 2).

### Analysis of the Phage Genome

All Myoviruses have a linear dsDNA genomes. However, initial assembly of the phiAGATE genome appeared to be circular, presumably due to presence of long, overlapping terminal repeats. This presumption was confirmed by the analysis of reads arrangement. We found 2669 bp long segment with mean coverage 1.9× higher than the rest of the sequence. It probably corresponds to overlapping LTRs. Moreover, the region is flanked by two tandem repeats (a 7-nt repeat on positions 145983–146061 and an 8-nt repeat on positions 2685–2735) that may be connected with formation of physical ends of DNA molecule. Bearing these findings in mind we set the starting point of the sequence to the first nucleotide of the mentioned segment. If our suppositions are true, complete genome of phage phiAGATE is 148844 bp long.

Its unique sequence consists of 147175 bp and has a GC content of 41.0%. Detailed analysis of this sequence resulted in prediction of 204 different CDSs (32 on the forward strand and 172 on the reverse strand, additionally five of them are repeated in LTR), three tRNA genes (for Asn, Met, Phe), and a sequence identified by tRNAscan as a Glu pseudogene. The most frequently recognized start codon is ATG (80.9%; TTG and GTG accounted for 9.8% and 9.3%, respectively), while the stop codon is TAA (63.7%, TAG –20.1%, TGA –16.2%). 108 of 204 predicted CDSs are similar to known sequences (with a BLASTp e-value of 1e-10 as a cut-off). Putative functions were assigned to 53 of them, and ontological terms to 49 (Table S3).

**Figure 3:**
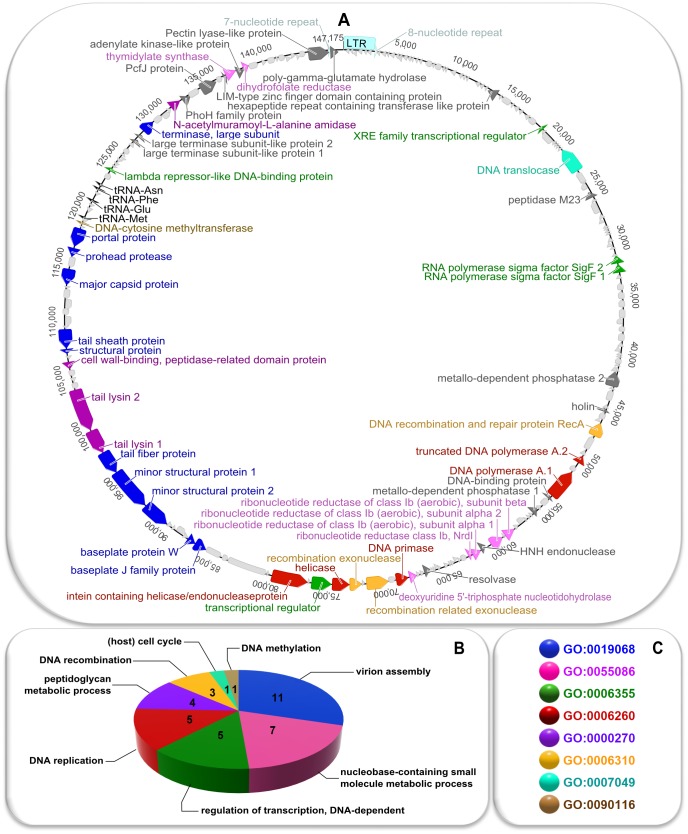
Genome organization of phiAGATE. Panel A shows a circular map of the genome unique sequence (right LTR not shown). The predicted CDSs, tRNA genes, and repeated regions are marked with arrows. Panel B shows distribution of Biological Process GO terms among the predicted protein products. Panel C explains color code in terms of GO identifiers of ontological terms. Colors are consistent in all sections of the figure: blue – GO:0019068– virion assembly, pink – GO:0055086– nucleobase-containing small molecule metabolic processes, green – GO:0006355– regulation of transcription, DNA-dependent, red – GO:0006260– DNA replication, mauve – GO:0000270– peptidoglycan metabolic processes, yellow – GO:0006310– DNA recombination, teal – GO:0007049– (host) cell cycle, light brown – GO:0090116– DNA methylation, dark grey – no GO term assigned, light grey – no function predicted, black – tRNA genes, azure – tandem repeats.

The genome has a modular structure typical for spounaviruses. Two groups of genes associated with DNA replication and recombination, together with a cluster of CDSs connected with nucleotides biosynthesis, form the replication module (located between ∼46 and ∼80 kb). Genes for structural proteins constitute the morphogenesis module (from ∼85 to ∼118 kb), which can further be divided into parts encoding the head and tail proteins. The latter include, among others, three CDSs that resemble known genes for enzymes involved in degradation of cell wall components: tail lysin 1 (containing a peptidase domain), tail lysin 2 (similar to known endo-beta-N-acetylglucosaminidases), and a 3D domain-containing protein (that is likely another peptidase). Surprisingly, the CDS encoding endolysin (N-acetylmuramoyl-L-alanine amidase) was found in the vicinity of a gene for a large terminase subunit, but not the one for the holin (located ∼60 kb away and orientated in the opposite direction). Of note, two proteins resembling known exopolymer-degrading depolymerases (the poly-γ-glutamate hydrolase and the pectin lyase-like protein) are also encoded in phiAGATE genome. The complete annotated sequence of this genome is available in GenBank under accession number JX238501.2. For detailed information about predicted CDSs and genome arrangement, see Figure 3 and Table S3.

### Phylogenetic Analyses

EM studies indicate that phiAGATE is a member of the *Myoviridae* family. Generally, the 20 top-scoring hits during BLASTp analysis of marker proteins originated either from members of the *Spounavirinae* subfamily, or from unclassified bacteriophages (see Table S1). Only the three lowest-scoring hits for the helicase matched proteins of *Tevenvirinae* phages. An analogous BLAST analysis of the mentioned unclassified viruses (namely *Bacillus* phages B4, B5S, BCP78, BCU4, BPS13, W.Ph., and staphylococcal phage JD007) yielded very similar results (although several markers showed slight similarity to bacterial sequences). We therefore concluded that phiAGATE, along with these phages, belong to the *Spounavirinae* subfamily. This was confirmed by clustering their genomes and genomes of 202 other myoviruses (retrived from RefSeq database) based on sequence similarity (*P* values of BLASTn HSPs) using CLANS. The bacteriophages in question grouped with known spounaviruses, while almost all other phages clustered according to their taxonomic affiliation (with the exception of the *Haemophilus* phage SuMu, that had little similarity to any other bacteriophage, though it is classified as a Mulikevirus; see Figure S1 and File S1).

**Figure 4:**
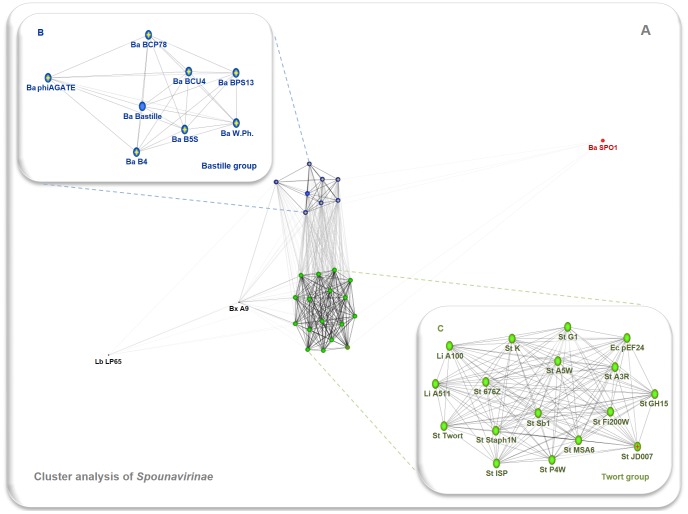
Results of clustering of Spounavirinae phages based on genomic similarity. Panel A shows result of cluster analysis of the whole group of studied phages. Panel B is a close-up showing layout of the Bastille group and panel C is a similar close-up depicting the Twort group. Edge weights were calculated from the *P* values of BLASTn high-scoring segment pairs (e-value cut-off equals 1e-5), and the resulting network was visualized in the CLANS software package (10000 layout rounds). Nodes are colored by the proposed in-subfamily clustering: blue – Bastille group, green – Twort group, red – Bacillus phage SPO1. Abbreviations include the name of host taxon (Ba – *Bacillus*, Bx – *Brochothrix*, En – *Enterococcus*, Lb – *Lactobacillus*, Li – *Listeria*, St – *Staphylococcus*) and the bacteriophage name. All analyzed sequences are listed in Table S2.

With this observation in mind we undertook a similar analysis at the subfamily level. The results (shown in Figure 4) indicate that *Spounavirinae* phages can be divided into two distinct groups. In the first cluster, all the ICTV-recognized Twortlikeviruses group with the remaining staphylococcal phages (some unclassified or described as SPO1-like viruses) and the *Enterococcus* phage phiEF24C. The second contain almost all the analyzed *Bacillus* bacteriophages (Bastille, B4, B5S, BCP78, BCU4, BPS13, W.Ph. and phiAGATE). For the purposes of the study, we coined provisional names for both clusters: Twort group and Bastille group, respectively. Surprisingly *Bacillus* phage SPO1 (a type species of genus *Spounalikevirus*) was a member of neither. Rather, it appeared as a distant singleton (compare Figure 4 and File S2).

**Figure 5:**
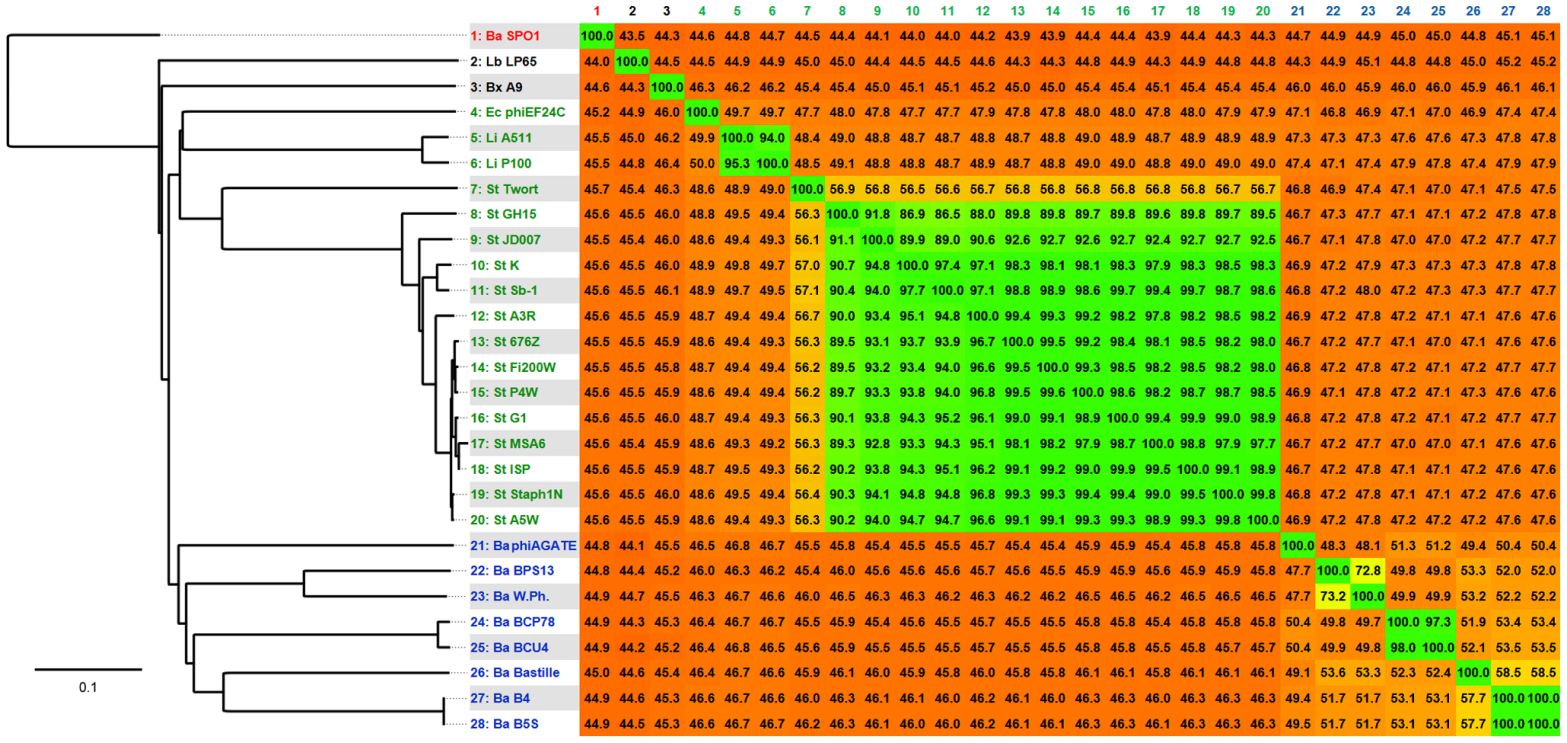
Heatmap showing the results of genome comparison of the studied spounaviruses, and the resulting phylogenomic tree. The similarity values were calculated using Gegenees software based on pairwise translated comparison of the analyzed sequences (tBLASTx method, fragment size –50, step size –25). The heat plot colors reflect this similarity, ranging from low (red) to high (green). The heatmap is asymmetric because the variable contents of genomes differ in sizes and a similarity is calculated as a fraction of similar sequences in each genome. The tree was constructed with SplitsTree using the neighbor joining method. The scale bar represents a 10% difference in average tBLASTx score. Leaves of the tree are colored by proposed in-subfamily clustering: blue – Bastille group, green – Twort group, red – Bacillus phage SPO1. Abbreviations include name of host taxon (Ba – *Bacillus*, Bx – *Brochothrix*, En – *Enterococcus*, Lb – *Lactobacillus*, Li – *Listeria*, St – *Staphylococcus*) and the bacteriophage name. All analyzed sequences are listed in Table S2.

Further evidence for the distinct status of the proposed clusters came from the phylogenomic tree based on translated comparison of whole phage genomes. We found that viruses from the Twort and Bastille groups form separate branches with a comparable distance to phage SPO1 (see Figure 5).

**Figure 6:**
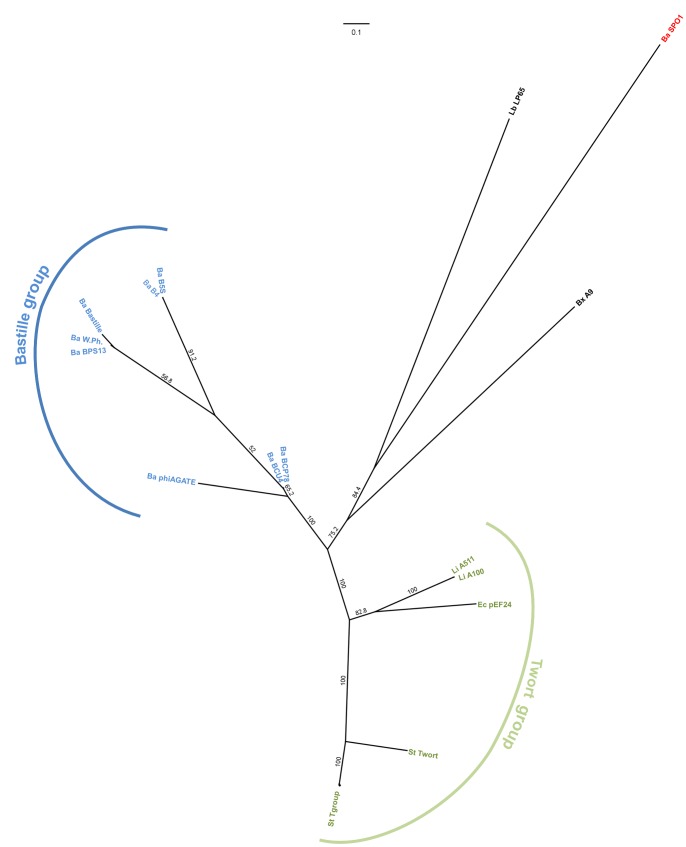
Majority consensus maximum likelihood tree (250 bootstrap replicates) based on comparison of all analyzed protein markers, showing the relationships between the analyzed spounaviruses. Branch labels indicate their percent bootstrap support. Leaves are colored by proposed in-subfamily clustering: blue – Bastille group, green – Twort group, red – Bacillus phage SPO1. Abbreviations include name of host taxon (Ba – *Bacillus*, Bx – *Brochothrix*, En – *Enterococcus*, Lb – *Lactobacillus*, Li – *Listeria*, St – *Staphylococcus*) and the bacteriophage name. All analyzed sequences are listed in Table S1.

To reconfirm our findings, we generated maximum likelihood trees based on the sequences of selected protein markers. In all final trees, distinct branches corresponding to the proposed groups could be observed (see Figure S2). Moreover, the mean patristic distance (calculated from branch lengths) between phages from the Bastille group and SPO1 or the Twort-group viruses exceeded the greatest in-group distance (see Table S4). Tests of congruence revealed that all single-marker trees were significantly more similar than would be expected by chance (I_cong_ ≥2.20; P-value of null hypothesis ≤9.19e-09; Table S5). Finally, when we prepared a condensed tree from all marker sequences, we obtained topology similar to that observed in the phylogenomic tree (see Figure 6) and the similarity of these trees was also confirmed by congruence analysis (I_cong_ = ∼2.07, P-value = ∼7.08e−08).

### Comparative Genomics

**Figure 7:**
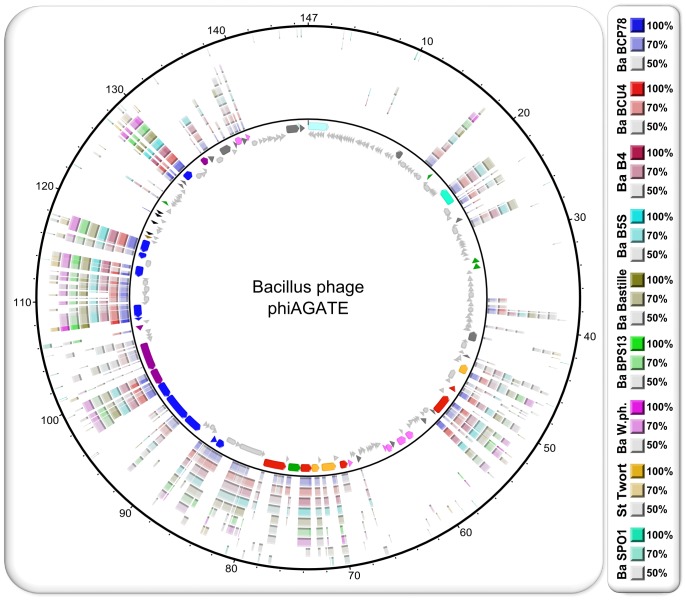
Genome comparison of phage phiAGATE, other members of the Bastille group, phage SPO1, and phage Twort, visualized with BRIG. The central ring is a circular map of the reference genome, in this case genome of phiAGATE. Each further ring represents a genome of another bacteriophage. Their order (starting from center) and colors are explained at the right side. The map color scheme is the same as in Figure 3 and should not be confused with the BRIG color code included in this figure (CDSs are colored according to assigned Biological Process GO terms: blue – virion assembly, pink - nucleobase-containing small molecule metabolic processes, green – regulation of transcription, DNA-dependent, red – DNA replication, mauve - peptidoglycan metabolic processes, yellow – DNA recombination, teal – (host) cell cycle, light brown – DNA methylation, dark grey – no GO term assigned, light grey – no function predicted, and tRNA genes are marked with black arrows while tandem repeats with azure ones).

**Figure 8:**
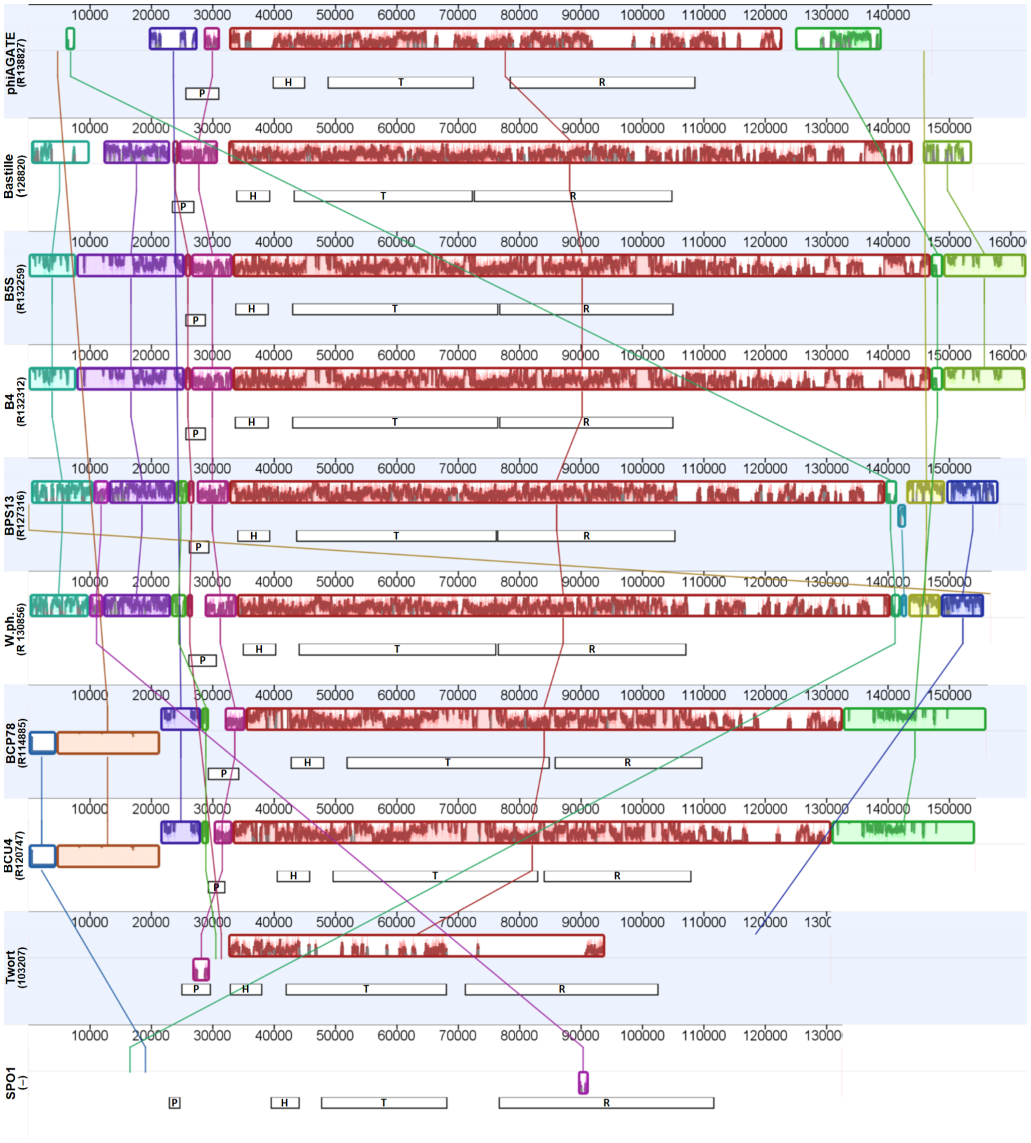
Whole genome comparisons between Bastille-group phages and type species of ICTV-recognized genera in the Spounavirinae subfamily. The genomes were compared using the progressive Mauve algorithm and visualized with the MAUVE plugin in the Geneious software suite. Each colored box represents a region that aligned to part of another genome (locally collinear block – LCB). Similarity inside the block is indicated by the height of the colored bars. The placement of a block below the axis indicates inversion, while the line connecting blocks represents a match between the regions. The putative functional modules (inferred from the annotation of the analyzed records) are shown as black and white boxes beneath LCBs (lowered position of the box indicates inverted orientation of genes in the module, symbols inside the boxes indicate: P – packaging module, H – head morphogenesis module, T – tail morphogenesis module, R – DNA replication module). All sequences (except the one of phage SPO1, which displays only negligible similarity to all others) were colinearized at the position indicated in brackets (based on the arrangement of the modules and pre-computed locally collinear blocks) to simplify the visualization. ‘R’ indicates that the genome was reversed prior to linearization. All analyzed sequences are listed in Table S2.

The modular organization of all the studied Bastille-group phages is very similar. Although insertions or deletions occasionally disrupt the gene order, the core modules tend to form large synthetic blocks arranged in the identical manner in every analyzed genome. Genes are transcribed in the same direction in the whole region containing the head and tail morphogenesis and replication modules. This large cluster of syntenic sequences is often preceded by another, containing CDSs encoding the large terminase subunit and endolysin (proximity of these sequences suggests connection between DNA packaging and lysis of the host cell), but in some cases these regions are split by several tRNAs genes (see Figure 7 and Figure 8).

We found somewhat similar arrangement of core modules in genomes of the phage Twort and (to a lesser extent) SPO1. However, in the case of these viruses, the similarity is weaker, many coding sequences diverged beyond recognition and the packaging modules appear to be reversed compared to the Bastille group (compare Figures 7, 8, and Figure S3).

The sequences localized outside the core regions seem to be less well conserved. While some similarities were observed, they were often restricted to certain Bastille-group subsets. The phiAGATE genome contain a large region with an extremely mosaic structure, in which only a few CDSs share significant similarity with other known *Spounavirinae* sequences. Some resemble genes from other, mostly unclassified phages (e.g. CDSs for pectin lyase-like protein, poly-gamma-glutamate hydrolase, or XRE family transcriptional regulator similar to those of phage phiNIT1), while other seem to be homologous to bacterial sequences (sometimes from organisms surprisingly distant from the phage host, such as *Haemophilus paraphrohaemolyticus* or *Pasteurella pneumotropica*). The remaining have no significant similarity with known sequences.

## Discussion

To isolate phages involved in the functioning of an ecosystem we used a strain known to be abundant in the search site (*Bacillus pumilus* GL1) as a candidate host. The approach turned out to be successful and a novel phage was discovered.

The virus features a vast genome densely covered by CDSs (occupying 87.5% of the sequence), only a fraction of which could be described in terms of putative function. The GC-content of phage genetic material (41.0%) is similar to that observed in genomes of *B. pumilus* (41.3–41.7%) [14], while the genome size, head diameter, and tail length fall within the ranges specified for *Spounavirinae* (127–157 kb, 75–100 nm, and 140–220 nm, respectively) [1]. Together with the presented results, this allows us to designate phage phiAGATE as a candidate species for this subfamily.

While affiliation of the virus with the *Spounavirinae* subfamily seems clear, there were difficulties in classifying it to the genus. Regardless of the chosen method, the bacteriophage failed to cluster with either phage SPO1 or Twort (type species of the only ICTV-accepted genera within the subfamily). Rather, it formed a distinct cluster (named the “Bastille group”) or branch of a phylogenetic tree together with the *Bacillus* bacteriophages B4, B5S, Bastille, BCP78, BCU4, BPS13, and W.Ph.

The modular arrangement of the phiAGATE genome appears typical of the Bastille group. Despite low sequence similarity, the synteny in the core sections of the analyzed genomes is apparent. Such a phenomenon has been observed in many groups of phages, but there remains controversy regarding its explanation. The order of genes can be retained in mosaic genomes when recombination events occurring during their evolution are: mainly legitimate, semi-legitimate (if there are conserved “joints” facilitating recombination on the boundaries of genes or modules) or illegitimate, but most non-homologous recombinants are eliminated by natural selection [84]. In turn, the selective pressure might be connected with the mechanism of genome packaging (which is sensitive to changes in the amount of genomic DNA) or an operon-based mode of transcription. However, the latter possibility seems unlikely under discussed circumstances; the syntenic regions are dozens of kb long and the genes of spounaviruses appear to be transcribed from more than 50 different promoters [7], [72]. It is therefore likely that at least several independent and non-overlapping transcriptional units cover single syntenic region. If the continuity of operons was the only factor limiting recombination, rearrangements between units would not be suppressed. Nevertheless, a hypothesis postulating that selective pressure retains structure of core sections of genomes seems to be very compelling, especially assuming that many of genes in the replication, morphogenesis, and DNA packaging modules evolved together. This assumption is (at least partially) supported by the high congruence of phylogenies based on protein markers derived from these modules. The mosaic evolution commonly described in phages might be restricted to other areas of the genome, e.g. regions containing phage homologues of the bacterial sequences, also known as morons (however, this term is, in its strictest sense, reserved for DNA elements inserted between a pair of phage genes when the genes of this same pair are adjacent in genomes of related bacteriophages [85]). While such genes may be conserved among certain groups of phages, their functions cannot usually be explained in terms of the direct need for their products during the phage replication cycle [86].

The CDS for the phiAGATE PhoH family protein (similar to bacterial phosphate starvation-related proteins) seems to be a good example. While many phages carry homologues of PhoH (including members of the Bastille group, see Figure 6), the function of these sequences is unknown [87].

Gene cluster containing CDSs for two sigma factors and DNA translocase might be another example. This region seem to be conserved among related *Bacillus* phages (see Figure 6) [66], [88]–[92] so, it likely provide some kind of selective advantage. At least two genes in the cluster have something in common: their products share significant similarity with proteins involved in sporulation. Translocase resembles the SpoIIIE proteins that transport DNA across the septum during endospore formation [93], while one of the sigma factors is most similar to the sporulation-related ones (see Table S3). It is possible that products of these genes somehow interfere with host control over endospore formation. A similar phenomenon has previously been observed for phages PMB1 and PMJ1 (along with a group of undescribed phage isolates infecting *B. pumilus*), which were able to restore sporulation ability to defective mutants during pseudolysogenic infection [35]. Although the effect could not be explained at the time, similar defective strains of *B. subtilis* carry mutations within the gene coding the polymerase β subunit or a locus known as spoCM-1 (and could also be converted to spore formers by a phage) [94]. The benefits to the virus from control of host sporulation remain unclear (perhaps infected endospores are a means of phage dispersal or long-term survival).

The genes encoding proteins that resemble known poly-gamma-glutamate hydrolase and pectin lyase-like proteins also raise a question about their possible function. Similar CDSs can be found in the genomes of several *Bacillus* bacteriophages (such as SP01, SP10, and SPP1 [7], [95], [96]). In the case of the phage phiNIT1, they form a region arranged in a manner nearly identical to that of phiAGATE [97]. The hydrolase substrate is probably poly-gamma-glutamic acid (γPGA), an anionic polymer that forms a protective capsule around the cells of numerous *Bacillus* species and facilitates their adhesion to certain surfaces [97]–[99]. The substrate of the pectin lyase-like protein remains unknown, however, some reports indicate that similar enzymes may disrupt biofilms by degrading matrix polysaccharides [100], [101]. We therefore hypothesize that both proteins are involved in either freeing phage progeny from the exopolymeric matrix of the host or clearing the phage path to the cell surface.

Most bacteriophages in the proposed Bastille group remain unclassified. Only the phage Bastille has been described as “SPO1-related” (Klumpp and Loessner, unpublished data) [14]. However, this assignment has been challenged by group led by Klumpp himself when they discovered similarities between the Twort and Bastille phages and included the latter in a cluster of “Twort-like” *Bacillus* bacteriophages (along with W.Ph.) [1].

Here we demonstrate that while the Bastille group is comparable to the genus *Twortlikevirus* in terms of diversity and genetic distance from SPO1, it is a separate evolutionary lineage that displays limited similarity to other spounaviruses. The unambiguous classification of Bastille group phages certainly requires further detailed studies. However, our results suggest that the current taxonomic arrangement of the *Spounavirinae* subfamily may eventually prove to be insufficient, and a revision (perhaps including a *Bastillelikevirus* genus) might be seriously considered.

## Supporting Information

Figure S1 **Results of clustering of Myoviridae phages based on genome similarity.** Edge weights were calculated from the *P* values of BLASTn high scoring segment pairs (e-value cut-off equals 1e-2) and the resulting network was visualized using CLANS (10000 layout rounds). Nodes are colored by taxonomic affiliation (retrieved from the RefSeq records or ICTV Virus Taxonomy database and explained in the Figure). Studied phages (*Bacillus* phages B4, B5S, BCP78, BCU4, BPS13, W.Ph. and staphylococcal phage JD007) are additionally marked with yellow stars. All analyzed sequences are listed in Table S2.
(TIF)

Figure S2 **Majority consensus maximum likelihood trees (250 bootstrap replicates) obtained by analysis of sequences of different protein markers.** Panel A shows tree based on comparison of DNA polymerases, B – DNA helicases, C – major capsid proteins, D – portal proteins, E – tail sheath proteins, and F – terminase large subunits. Leaves are colored by proposed in-subfamily clustering: blue – Bastille group, green – Twort group, red – Bacillus phage SPO1. Abbreviations include name of host taxon (Ba – *Bacillus*, Bx – *Brochothrix*, En – *Enterococcus*, Lb – *Lactobacillus*, Li – *Listeria*, St – *Staphylococcus*) and the bacteriophaFge name. All analyzed sequences are listed in Table S1.
(TIF)

Figure S3 **Genome comparisons of phage SPO1, phage Twort, phage Bastille, and other members of the Bastille group, visualized with BRIG.** The central circle of each comparison represents a reference genome (SPO1 in panel A, Twort in section B, Bastille in section C). Each further ring represents a genome of a different phage. Their order and colors are explained in panel D.
(TIFF)

Table S1 **Protein markers and the results of their BLAST analysis.** Sheet 1 includes a description of all marker protein sequences used in phylogenetic analyses. Sheets 2–8 list 20 top scoring hits obtained for each marker from every analyzed phage during BLASTp searches against the non-redundant protein database. The taxonomic position of each BLAST hit is highlighted by the following colors: green (Spounavirinae), blue (Myoviruses with no subfamily or genus affiliation), red (Tevenvirinae), light brown (unclassified phages) or yellow (Bacteria). Records marked with “*” contain ambiguous annotations (in-record classification: “unclassified ssRNA viruses” inconsistent with provided literature data) and were marked as unclassified and excluded from further analyses.
(XLS)

Table S2 **List of reference genomic sequences.** Section A includes sequences of the analyzed phages, section B includes genomes of the remaining spounaviruses, section C comprises a full list of all other *Myoviridae* sequences used in the cluster analysis, while section D lists the references for all genomes. The abbreviation d.s. (direct submission) indicates that there are no references available in the record.
(PDF)

Table S3 **Complete list of all CDSs predicted in the phiAGATE genome.** Section A shows CDSs with predicted function, section B lists CDSs with no function assigned that are similar to other sequences from non-redundant protein database and section C includes CDSs that don’t share significant similarity with any known sequences (BLASTp e-value of 1e-10 was used as a cut-off).
(PDF)

Table S4 **Table of patristic distances between analyzed spounaviruses and the results of translated comparison of their genomes.** Worksheets 1–7 show patristic distances (calculated from branch lengths) in trees based on protein marker analyses. Sheet 8 presents corresponding distances for the phylogenomic tree. Sheet 9 shows results of translated comparison (performed with Gegenees) while sheet 10 summarizes all these data for proposed in-subfamily groups.
(XLS)

Table S5 **Results of congruence analysis.**
(XLS)

File S1 **Resulting file of 3D clustering of** ***Myoviridae*** **phages performed using the CLANS software package.** The file is compressed in the rar format. After unpacking it may be opened using CLANS (which can be downloaded from http://bioinfoserver.rsbs.anu.edu.au/programs/clans; the blast binaries are not required). The file is fully editable and searchable. The taxonomic assignment of the nodes can be retrieved using the Windows/Edit Groups functionality in CLANS. Settings are the same as described in the legend of Figure S1 (BLASTn as a comparison algorithm, the e-value cut-off equal to 1e-2, 10000 layout rounds)
(RAR)

File S2 **Resulting file of 3D clustering of** ***Spounavirinae*** **phages performed using the CLANS software package.** The file is compressed in the rar format. After unpacking it may be opened using CLANS (which can be downloaded from http://bioinfoserver.rsbs.anu.edu.au/programs/clans; the blast binaries are not required). The file is fully editable and searchable. The taxonomic assignment of the nodes can be retrieved using the Windows/Edit Groups functionality in CLANS. Settings are the same as described in legend of Figure 4 ((BLASTn as a comparison algorithm, the e-value cut-off equal to 1E-5, 10000 layout rounds)
(RAR)
