# Supplementary figures and images for: The Discovery of phiAGATE, A Novel Phage Infecting Bacillus pumilus, Leads to New Insights into the Phylogeny of the Subfamily Spounavirinae
Source: PLoS One. 2014 Jan 23;9(1):e86632. doi: 10.1371/journal.pone.0086632 (PMC3900605; doi:10.1371/journal.pone.0086632)

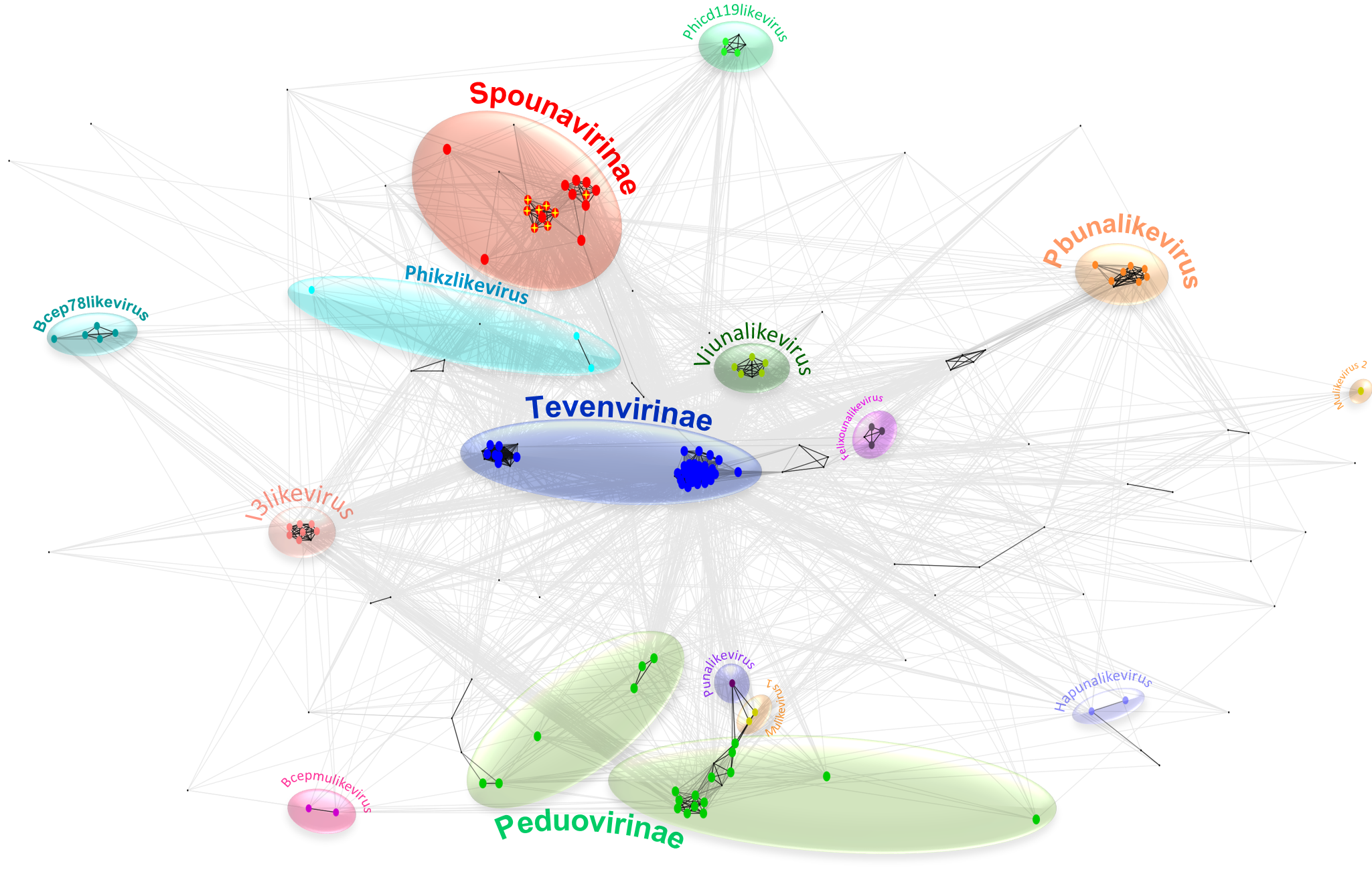

Supplement: Figure S1 — Results of clustering of Myoviridae phages based on genome similarity. Edge weights were calculated from the P values of BLASTn high scoring segment pairs (e-value cut-off equals 1e-2) and the resulting network was visualized using CLANS (10000 layout rounds). Nodes are colored by taxonomic affiliation (retrieved from the RefSeq records or ICTV Virus Taxonomy database and explained in the Figure). Studied phages (Bacillus phages B4, B5S, BCP78, BCU4, BPS13, W.Ph. and staphylococcal phage JD007) are additionally marked with yellow stars. All analyzed sequences are listed in Table S2. (TIF) [file pone.0086632.s001.tif]

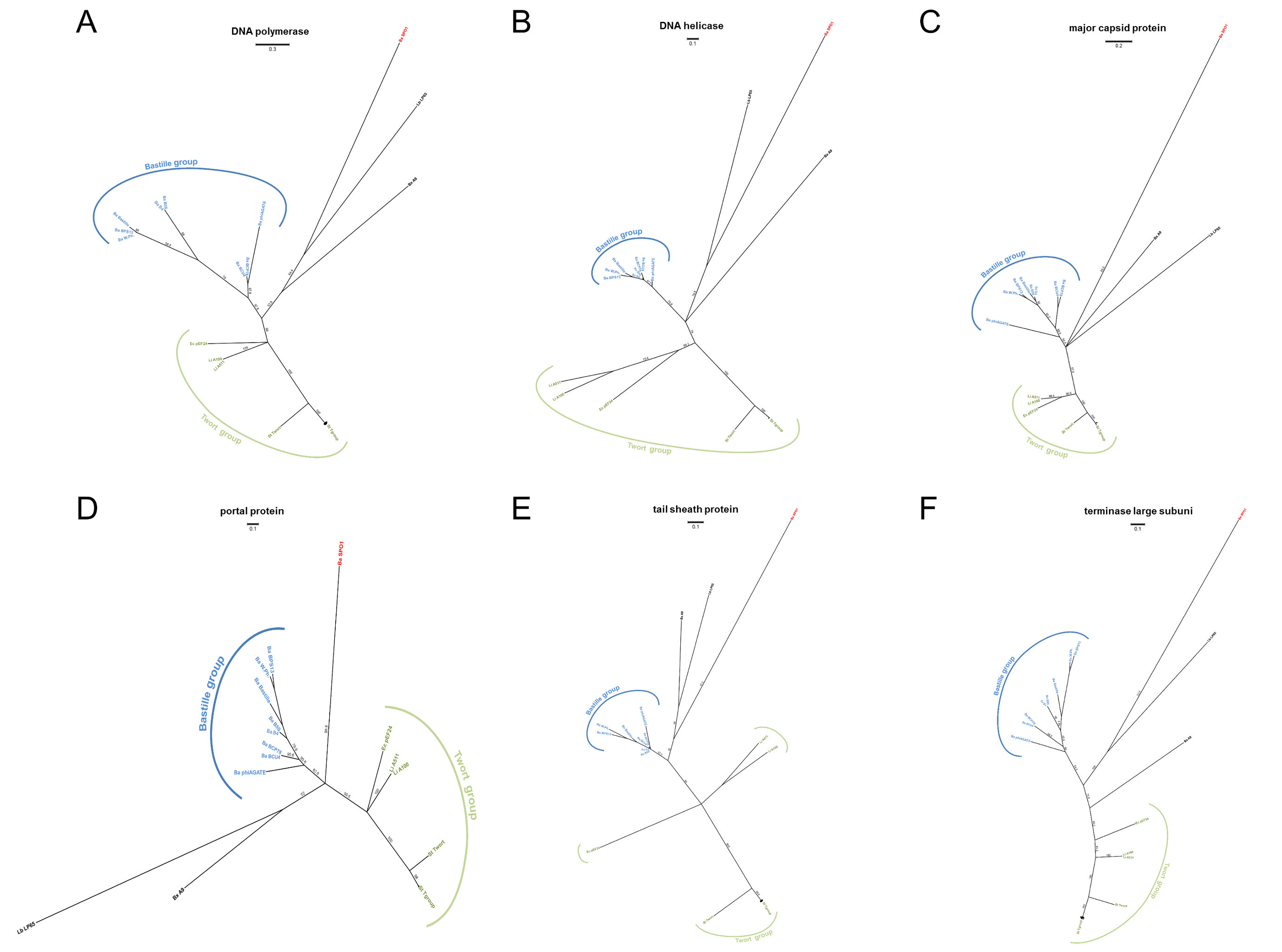

Supplement: Figure S2 — Majority consensus maximum likelihood trees (250 bootstrap replicates) obtained by analysis of sequences of different protein markers. Panel A shows tree based on comparison of DNA polymerases, B – DNA helicases, C – major capsid proteins, D – portal proteins, E – tail sheath proteins, and F – terminase large subunits. Leaves are colored by proposed in-subfamily clustering: blue – Bastille group, green – Twort group, red – Bacillus phage SPO1. Abbreviations include name of host taxon (Ba – Bacillus, Bx – Brochothrix, En – Enterococcus, Lb – Lactobacillus, Li – Listeria, St – Staphylococcus) and the bacteriophaFge name. All analyzed sequences are listed in Table S1. (TIF) [file pone.0086632.s002.tif]

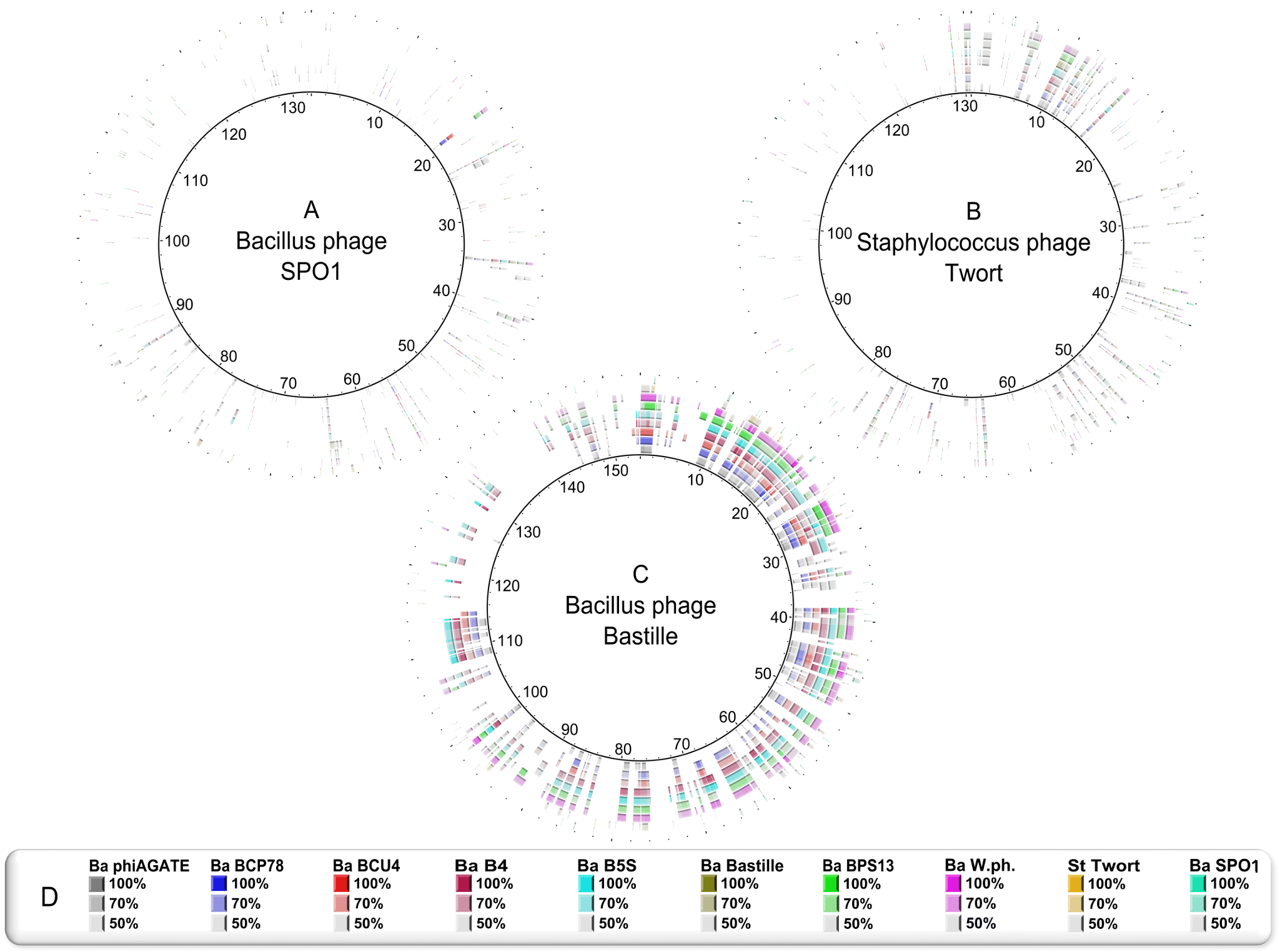

Supplement: Figure S3 — Genome comparisons of phage SPO1, phage Twort, phage Bastille, and other members of the Bastille group, visualized with BRIG. The central circle of each comparison represents a reference genome (SPO1 in panel A, Twort in section B, Bastille in section C). Each further ring represents a genome of a different phage. Their order and colors are explained in panel D. (TIFF) [file pone.0086632.s003.tiff]
