# Supplementary material for: The Discovery of phiAGATE, A Novel Phage Infecting Bacillus pumilus, Leads to New Insights into the Phylogeny of the Subfamily Spounavirinae
Source: PLoS One. 2014 Jan 23;9(1):e86632. doi: 10.1371/journal.pone.0086632 (PMC3900605; doi:10.1371/journal.pone.0086632)
Supplement: Table S2 — List of reference genomic sequences. Section A includes sequences of the analyzed phages, section B includes genomes of the remaining spounaviruses, section C comprises a full list of all other Myoviridae sequences used in the cluster analysis, while section D lists the references for all genomes. The abbreviation d.s. (direct submission) indicates that there are no references available in the record. (PDF) [file pone.0086632.s005.pdf]

A. Analyzed phages

Phage name:	Current NCBI Taxonomy	Seq. Lenght	Accesion	Reference
**Bacillus phage B4**	...Myoviridae	162596	NC_018863.1	[d.s.]
**Bacillus phage B5S**	...Myoviridae	162598	JN797796.1	[d.s.]
**Bacillus phage BCP78**	...Myoviridae	156176	NC_018860.1	[S1]
**Bacillus phage BCU4**	...Myoviridae	154371	JN797798.1	[d.s.]
**Bacillus phage BPS13**	...Myoviridae	158305	NC_018857.1	[S2]
**Bacillus phage phiAGATE**	...Caudovirales	147175	NC_020081.1	[d.s.]
**Bacillus phage W.Ph.**	...Myoviridae	156897	NC_016563.1	[d.s.]
**Staphylococcus phage JD007**	...Myoviridae	141836	NC_019726.1	[S3]

B. Other *Spounavirinae*

Phage name:	Current NCBI Taxonomy	Seq. Lenght	Accesion	Reference
**Bacillus phage Bastille**	…Spounavirinae; SPO1-like viruses; unclassified SPO1-like viruses	153962	NC_018856.1	[d.s.]
**Bacillus phage SPO1**	...Spounavirinae; SPO1-like viruses	132562	NC_011421.1	[d.s.]
**Brochothrix phage A9**	...Spounavirinae; unclassified Spounavirinae	127065	NC_015253.1	[S4]
**Enterococcus phage phiEF24C**	...Spounavirinae; unassigned Spounavirinae	142072	NC_009904.1	[S5]
**Lactobacillus phage LP65**	...Spounavirinae; unassigned Spounavirinae	131522	NC_006565.1	[S6]
**Listeria phage A511**	...Spounavirinae; Twortlikevirus	137619	NC_009811.2	[S7]
**Listeria phage P100**	...Spounavirinae; Twortlikevirus	131384	DQ004855.1	[S8]
**Staphylococcus phage 676Z**	...Spounavirinae; Twortlikevirus; unclassified Twortlikevirus	140115	JX080302.1	[S9]
**Staphylococcus phage A3R**	...Spounavirinae; Twortlikevirus; unclassified Twortlikevirus	132712	JX080301.1	[S9]
**Staphylococcus phage A5W**	...Spounavirinae; Twortlikevirus	137083	EU418428.1	[d.s.]
**Staphylococcus phage Fi200W**	...Spounavirinae; unclassified Spounavirinae	140079	JX080303.1	[S9]
**Staphylococcus phage G1**	...Spounavirinae; Twortlikevirus	138715	NC_007066.1	[S10]
**Staphylococcus phage GH15**	...Spounavirinae; Twortlikevirus; unclassified Twortlikevirus	139806	NC_019448.1	[S11]
**Staphylococcus phage ISP**	...Spounavirinae; Twortlikevirus	138339	FR852584.1	[S12]
**Staphylococcus phage K**	...Spounavirinae; Twortlikevirus	127395	NC_005880.1	[S13]
**Staphylococcus phage MSA6**	...Spounavirinae; unclassified Spounavirinae	140194	JX080304.1	[S9]
**Staphylococcus phage P4W**	...Spounavirinae; unclassified Spounavirinae	139173	JX080305.1	[S9]
**Staphylococcus phage Sb-1**	...Spounavirinae; SPO1-like viruses; unclassified SPO1-like viruses	127188	HQ163896.1	[S14]
**Staphylococcus phage Staph1N**	...Spounavirinae; unclassified Spounavirinae	137192	JX080300.1	[S9]
**Staphylococcus phage Twort**	...Spounavirinae; Twortlikevirus	130706	NC_007021.1	[S10]

C. Other *Myoviridae*

Phage name:	Current NCBI Taxonomy	Seq. Lenght	Accesion	Reference
**Acinetobacter phage 133**	...Tevenvirinae; T4likevirus	159801	NC_015250.1	[S15]
**Acinetobacter phage Ac42**	...Tevenvirinae; T4likevirus	167716	NC_014660.1	[S15]
**Acinetobacter phage Acj9**	...Tevenvirinae; T4likevirus	169947	NC_014663.1	[S15]
**Acinetobacter phage Acj61**	...Tevenvirinae; T4likevirus	164093	NC_014661.1	[S15]
**Acinetobacter phage ZZ1**	...Myoviridae	166682	NC_018087.1	[S16]
**Aeromonas phage 25**	...Tevenvirinae; T4likevirus	161475	NC_008208.1	[S17]
**Aeromonas phage 31**	...Tevenvirinae; T4likevirus	172963	NC_007022.1	[S17]
**Aeromonas phage 44RR2.8t**	...Tevenvirinae; T4likevirus	173591	NC_005135.1	[S18]
**Aeromonas phage 65**	...Tevenvirinae; T4likevirus	235229	NC_015251.1	[S15]
**Aeromonas phage Aeh1**	...Tevenvirinae; T4likevirus	233234	NC_005260.1	[S18]
**Aeromonas phage Aes508**	...Tevenvirinae; T4likevirus	160646	NC_019543.1	[d.s.]
**Aeromonas phage CC2**	...Tevenvirinae; T4likevirus	231743	NC_019538.1	[S19]
**Aeromonas phage phiAS4**	...Tevenvirinae; T4likevirus	163875	NC_014635.1	[S20]
**Aeromonas phage phiAS5**	...Tevenvirinae; T4likevirus	225268	NC_014636.1	[S21]
**Aeromonas phage phiO18P**	...Peduovirinae; Hpunalikevirus	33985	NC_009542.2	[d.s.]
**Aeromonas phage vB_AsaM-56**	...Myoviridae	43551	NC_019527.1	[S22]
**Aggregatibacter phage S1249**	...Myoviridae	43970	NC_013597.1	[S23]
**Agrobacterium phage 7-7-1**	...Myoviridae	69391	NC_019519.1	[d.s.]
**Bacillus phage 0305phi8-36**	...Myoviridae	218948	NC_009760.1	[S24] [S25]
**Bacillus phage BCD7**	...Myoviridae	93839	NC_019515.1	[d.s.]
**Bacillus phage SP10**	...Myoviridae	143986	NC_019487.1	[S26]
**Bdellovibrio phage phi1402**	...Myoviridae	23931	NC_015721.1	[S27]
**Bdellovibrio phage phi1422**	...Myoviridae	45354	NC_019525.1	[S22]
**Burkholderia phage Bcep1**	...Myoviridae	48177	NC_005263.2	[S28]
**Burkholderia phage Bcep43**	...Myoviridae	48024	NC_005342.2	[S28]
**Burkholderia phage Bcep781**	...Myoviridae	48247	NC_004333.2	[S28]
**Burkholderia phage BcepB1A**	...Myoviridae	47399	NC_005886.2	[S28]
**Burkholderia phage BcepF1**	...Myoviridae	72415	NC_009015.1	[d.s.]
**Burkholderia phage BcepMu**	...Myoviridae	36748	NC_005882.1	[S29]
**Burkholderia phage BcepNY3**	...Myoviridae	47382	NC_009604.1	[d.s.]
**Burkholderia phage KL3**	...Peduovirinae; P2likeviruses	40555	NC_015266.1	[S30]
**Burkholderia phage KS5**	...Peduovirinae; P2likeviruses	37236	NC_015265.1	[S30]
**Burkholderia phage KS10**	...Myoviridae	37635	NC_011216.1	[S31]
**Burkholderia phage KS14**	...Peduovirinae; P2likeviruses	32317	NC_015273.1	[S30]
**Burkholderia phage phi52237**	...Peduovirinae; P2likeviruses	37639	NC_007145.2	[d.s.]
**Burkholderia phage phiE12-2**	...Peduovirinae; P2likeviruses	36690	NC_009236.1	[d.s.]
**Burkholderia phage phiE202**	...Peduovirinae; P2likeviruses	35741	NC_009234.1	[d.s.]
**Burkholderia phage phiE255**	...Myoviridae	37446	NC_009237.1	[d.s.]
**Campylobacter phage CP21**	...Myoviridae	182833	NC_019507.1	[S32]
**Campylobacter phage CPX**	...Myoviridae	132662	NC_016562.1	[d.s.]
**Campylobacter phage NCTC12673**	...Myoviridae	135041	NC_015464.1	[S33]
**Clostridium phage c-st**	...Myoviridae	185683	NC_007581.1	[S34]
**Clostridium phage phiC2**	...Myoviridae	56538	NC_009231.1	[S35]
**Clostridium phage phiCD27**	...Myoviridae	50930	NC_011398.1	[S36]
**Clostridium phage phiCD119**	...Myoviridae	53325	NC_007917.1	[S37]
**Clostridium phage phiMMP02**	...Myoviridae	48396	NC_019421.1	[S38]
**Clostridium phage phiMMP04**	...Myoviridae	31674	NC_019422.1	[S38]
**Cronobacter phage CR3**	...Myoviridae	149273	NC_017974.1	[S39]
**Cronobacter phage ENT47670**	...Myoviridae	47611	NC_019927.1	[d.s.]
**Cronobacter phage vB_CsaM_GAP31**	...Myoviridae	147940	NC_019400.1	[S40]
**Cronobacter phage vB_CsaM_GAP32**	...Myoviridae	358663	NC_019401.1	[d.s.]
**Cronobacter phage vB_CsaM_GAP161**	...Tevenvirinae; T4likevirus	178193	NC_019398.1	[S41]
**Cyanophage S-TIM5**	...Myoviridae	161440	NC_019516.1	[S42]
**Deftia phage phiW-14**	...Myoviridae	157486	NC_013697.1	[d.s.]
**Enterobacteria phage Bp7**	...Tevenvirinae; T4likevirus	168066	NC_019500.1	[S43]
**Enterobacteria phage CC31**	...Tevenvirinae; T4likevirus	165540	NC_014662.1	[S15]
**Enterobacteria phage HX01**	...Tevenvirinae; T4likevirus	169158	NC_018855.1	[S44]
**Enterobacteria phage IME08**	...Tevenvirinae; T4likevirus	172253	NC_014260.1	[S45] [S46]
**Enterobacteria phage ime09**	...Tevenvirinae; T4likevirus	166499	NC_019503.1	[d.s.]
**Enterobacteria phage JS10**	...Tevenvirinae; T4likevirus	171451	NC_012741.1	[S47]
**Enterobacteria phage JS98**	...Tevenvirinae; T4likevirus	170523	NC_010105.1	[S48] [S49]
**Enterobacteria phage JSE**	...Tevenvirinae; T4likevirus	166418	NC_012740.1	[S47]
**Enterobacteria phage Mu**	...Mulikevirus	36717	NC_000929.1	[S50]

C. Other *Myoviridae* (continued)

Phage name:	Current NCBI Taxonomy	Seq. Lenght	Accesion	Reference
**Enterobacteria phage P1**	...Punalikevirus	94800	NC_005856.1	[S51]
**Enterobacteria phage P2**	...Peduovirinae; P2likeviruses	33593	NC_001895.1	[S52] - [S74]
**Enterobacteria phage P4**	...Peduovirinae; P2likeviruses	11624	NC_001609.1	[S58] [S75] [S76]
**Enterobacteria phage Phi1**	...Tevenvirinae; T4likevirus	164270	NC_009821.1	[S77]
**Enterobacteria phage phiEcoM-GJ1**	...Myoviridae	52975	NC_010106.1	[S78]
**Enterobacteria phage phiP27**	...Myoviridae	42575	NC_003356.1	[S79]
**Enterobacteria phage PsP3**	...Peduovirinae; P2likeviruses	30636	NC_005340.1	[S80] [S81]
**Enterobacteria phage RB14**	...Tevenvirinae; T4likevirus	165429	NC_012638.1	[d.s.]
**Enterobacteria phage RB16**	...Tevenvirinae; T4likevirus	176788	NC_014467.1	[S15]
**Enterobacteria phage RB32**	...Tevenvirinae; T4likevirus	165890	NC_008515.1	[d.s.]
**Enterobacteria phage RB43**	...Tevenvirinae; T4likevirus	180500	NC_007023.1	[d.s.]
**Enterobacteria phage RB49**	...Tevenvirinae; T4likevirus	164018	NC_005066.1	[S18] [S82] [S83] [S84] [S85] [S86]
**Enterobacteria phage RB51**	...Tevenvirinae; T4likevirus	168394	NC_012635.1	[d.s.]
**Enterobacteria phage RB69**	...Tevenvirinae; T4likevirus	167560	NC_004928.1	[S18] [S87] [S88] [S89]
**Enterobacteria phage SfV**	...Myoviridae	37074	NC_003444.1	[S90] [S91] [S92]
**Enterobacteria phage T4**	...Tevenvirinae; T4likevirus	168903	NC_000866.4	[S93] - [S184]
**Enterobacteria phage vB_EcoM_ACG-C40**	...Tevenvirinae; T4likevirus	167396	NC_019399.1	[d.s.]
**Enterobacteria phage vB_EcoM-FV3**	...Myoviridae	136947	NC_019517.1	[S185]
**Enterobacteria phage vB_EcoM-VR7**	...Tevenvirinae; T4likevirus	169285	NC_014792.1	[S186] [S187]
**Enterobacteria phage vB_KleM-RaK2**	...Myoviridae	345809	NC_019526.1	[S188]
**Erwinia phage ENT90**	...Myoviridae	29564	NC_019932.1	[d.s.]
**Erwinia phage phiEa21-4**	...Myoviridae	84576	NC_011811.1	[S189]
**Erwinia phage phiEa104**	...Myoviridae	84565	NC_015292.1	[S190]
**Erwinia phage phiEt88**	...Myoviridae	47279	NC_015295.1	[S190]
**Erwinia phage vB_EamM-Y2**	...Myoviridae	56621	NC_019504.1	[S191]
**Escherichia phage Cba120**	...Myoviridae	157304	NC_016570.1	[d.s.]
**Escherichia phage D108**	...Mulikevirus; unclassified Mu-like viruses	37235	NC_013594.1	[d.s.]
**Escherichia phage PhaxI**	...Myoviridae	156628	NC_019452.1	[d.s.]
**Escherichia phage wV7**	...Tevenvirinae; T4likevirus	166452	NC_019505.1	[S192]
**Escherichia phage wV8**	...Myoviridae	88487	NC_012749.1	[S193]
**Haemophilus phage Aaphi23**	...Myoviridae	43033	NC_004827.1	[S194]
**Haemophilus phage HP1**	...Peduovirinae; Hpunalikevirus	32355	NC_001697.1	[S195] [S196] [S197] [S198] [S199] [S200]
**Haemophilus phage HP2**	...Peduovirinae; Hpunalikevirus	31508	NC_003315.1	[S201]
**Haemophilus phage SuMu**	...Mulikevirus; unclassified Mu-like viruses	37151	NC_019455.1	[S202] [S203]
**Halocynthia phage JM-2012**	...Myoviridae	167292	NC_017975.1	[d.s.]
**Halomonas phage phiHAP-1**	...Myoviridae	39245	NC_010342.1	[S204]
**Iodobacteriophage phiPLPE**	...Myoviridae	47453	NC_011142.1	[S205]
**Klebsiella phage KP15**	...Tevenvirinae; T4likevirus	174436	NC_014036.1	[d.s.]
**Lactobacillus phage KC5a**	...Myoviridae	38239	NC_007924.1	[d.s.]
**Lactobacillus phage Lb338-1**	...Myoviridae	142111	NC_012530.1	[S206]
**Mannheimia phage phiMHaA1**	...Peduovirinae; P2likeviruses	34525	NC_008201.1	[S207]
**Microcystis aeruginosa phage Ma-LMM01**	...Myoviridae	162109	NC_008562.1	[S208]
**Mycobacterium phage Bxz1**	...I3likevirus; unclassified I3-like viruses	156102	NC_004687.1	[S209]
**Mycobacterium phage Cali**	...I3likevirus; unclassified I3-like viruses	155372	NC_011271.1	[d.s.]
**Mycobacterium phage Catera**	...I3likevirus; unclassified I3-like viruses	153766	NC_008207.1	[S210]
**Mycobacterium phage ET08**	...I3likevirus; unclassified I3-like viruses	155445	NC_013650.1	[S211]
**Mycobacterium phage Myrna**	...Myoviridae	164602	NC_011273.1	[d.s.]
**Mycobacterium phage Rizal**	...I3likevirus; unclassified I3-like viruses	153894	NC_011272.1	[d.s.]
**Mycobacterium phage ScottMcG**	...I3likevirus; unclassified I3-like viruses	154017	NC_011269.1	[d.s.]
**Mycobacterium phage Spud**	...I3likevirus; unclassified I3-like viruses	154906	NC_011270.1	[d.s.]
**Natrialba phage PhiCh1**	...Myoviridae	58498	NC_004084.1	[S212] [S213] [S214]
**Pasteurella phage F108**	...Peduovirinae; Hpunalikevirus	30505	NC_008193.1	[S215]
**Pectobacterium phage ZF40**	...Myoviridae	48454	NC_019522.1	[S22]
**Prochlorococcus phage P-HM1**	...Myoviridae	181044	NC_015280.1	[S216]
**Prochlorococcus phage P-HM2**	...Myoviridae	183806	NC_015284.1	[S216]
**Prochlorococcus phage P-RSM4**	...Myoviridae	176428	NC_015283.1	[S216]
**Prochlorococcus phage P-SSM2**	...Tevenvirinae; T4likevirus	252401	NC_006883.2	[S216] [S217]
**Prochlorococcus phage P-SSM4**	...Tevenvirinae; T4likevirus	178249	NC_006884.2	[S216] [S217]
**Prochlorococcus phage P-SSM7**	...Myoviridae	182180	NC_015290.1	[S216]
**Pseudomonas phage 14-1**	...Myoviridae	66235	NC_011703.1	[S218]
**Pseudomonas phage 201phi2-1**	...Phikzlikevirus; unclassified phiKZ-like viruses	316674	NC_010821.1	[S219] [S220]
**Pseudomonas phage EL**	...Phikzlikevirus	211215	NC_007623.1	[S221] [S222] [S223] [S224]
**Pseudomonas phage F8**	...Myoviridae	66015	NC_007810.1	[S225]

C. Other *Myoviridae* (continued)

Phage name:	Current NCBI Taxonomy	Seq. Lenght	Accesion	Reference
**Pseudomonas phage JG004**	...Myoviridae	93017	NC_019450.1	[S226]
**Pseudomonas phage JG024**	...Myoviridae	66275	NC_017674.1	[S227]
**Pseudomonas phage KPP10**	...Myoviridae	88322	NC_015272.1	[S228]
**Pseudomonas phage KPP12**	...Myoviridae	64144	NC_019935.1	[S229]
**Pseudomonas phage LBL3**	...Myoviridae	64427	NC_011165.1	[S218] [S230]
**Pseudomonas phage LMA2**	...Myoviridae	66530	NC_011166.1	[S218] [S230]
**Pseudomonas phage Lu11**	...Myoviridae	280538	NC_017972.1	[S231]
**Pseudomonas phage NH-4**	...Myoviridae	66116	NC_019451.1	[S232]
**Pseudomonas phage OBP**	...Myoviridae	284757	NC_016571.1	[S233]
**Pseudomonas phage PAK_P1**	...Myoviridae	93398	NC_015294.1	[S234]
**Pseudomonas phage PaP1**	...Myoviridae	91715	NC_019913.1	[d.s.]
**Pseudomonas phage PB1**	...Myoviridae	65764	NC_011810.1	[S218]
**Pseudomonas phage phiCTX**	...Peduovirinae; P2likeviruses	35580	NC_003278.1	[S235] [S236] [S237]
**Pseudomonas phage phiKZ**	...Phikzlikevirus	280334	NC_004629.1	[S238] [S239]
**Pseudomonas phage SN**	...Myoviridae	66390	NC_011756.1	[S218]
**Pseudomonas phage vB_PaeM_C2-10_Ab1**	...Myoviridae	92777	NC_019918.1	[d.s.]
**Ralstonia phage RSA1**	...Peduovirinae; P2likeviruses	38760	NC_009382.1	[S240]
**Ralstonia phage RSL1**	...Myoviridae	231255	NC_010811.2	[S241]
**Rhodothermus phage RM378**	...Myoviridae	129908	NC_004735.1	[S242]
**Salmonella phage FelixO1**	...Myoviridae	86155	NC_005282.1	[d.s.]
**Salmonella phage Fels-2**	...Peduovirinae; P2likeviruses	33693	NC_010463.1	[S243]
**Salmonella phage PhiSH19**	...Myoviridae	157785	NC_019530.1	[S244]
**Salmonella phage PVP-SE1**	...Myoviridae	145964	NC_016071.1	[S245]
**Salmonella phage RE-2010**	...Myoviridae	34117	NC_019488.1	[S246]
**Salmonella phage SFP10**	...Myoviridae	157950	NC_016073.1	[S247]
**Salmonella phage SKML-39**	...Myoviridae	159624	NC_019910.1	[d.s.]
**Salmonella phage ViI**	...Myoviridae	157061	NC_015296.1	[S248]
**Shigella phage Ag3**	...Myoviridae	158006	NC_013693.1	[d.s.]
**Shigella phage Shfl2**	...Tevenvirinae; T4likevirus	165919	NC_015457.1	[d.s.]
**Shigella phage SP18**	...Tevenvirinae; T4likevirus	170605	NC_014595.1	[S249]
**Sphingomonas phage PAU**	...Myoviridae	219372	NC_019521.1	[d.s.]
**Streptococcus phage EJ-1**	...Myoviridae	42935	NC_005294.1	[S250] [S251]
**Synechococcus phage metaG-MbCM1**	...Tevenvirinae; T4likevirus	172879	NC_019443.1	[d.s.]
**Synechococcus phage S-CRM01**	...Myoviridae	178563	NC_015569.1	[S252]
**Synechococcus phage S-MbCM6**	...Tevenvirinae; T4likevirus	176043	NC_019444.1	[d.s.]
**Synechococcus phage S-PM2**	...Tevenvirinae; T4likevirus	196280	NC_006820.1	[S253] [S254]
**Synechococcus phage S-RSM4**	...Tevenvirinae; T4likevirus	194454	NC_013085.1	[S255]
**Synechococcus phage S-ShM2**	...Myoviridae	179563	NC_015281.1	[S216]
**Synechococcus phage S-SM1**	...Myoviridae	174079	NC_015282.1	[S216]
**Synechococcus phage S-SM2**	...Myoviridae	190789	NC_015279.1	[S216]
**Synechococcus phage S-SSM5**	...Myoviridae	176184	NC_015289.1	[S216]
**Synechococcus phage S-SSM7**	...Myoviridae	232878	NC_015287.1	[S216]
**Synechococcus phage syn9**	...Tevenvirinae; T4likevirus	177300	NC_008296.2	[S256]
**Synechococcus phage Syn19**	...Myoviridae	175230	NC_015286.1	[S216]
**Thermus phage phiYS40**	...Myoviridae	152372	NC_008584.1	[S257]
**Thermus phage TMA**	...Myoviridae	151483	NC_015937.1	[S258]
**Vibrio phage CP-T1**	...Myoviridae	44492	NC_019457.1	[S22]
**Vibrio phage ICP1**	...Myoviridae	125956	NC_015157.1	[S259]
**Vibrio phage K139**	...Peduovirinae; Hpunalikevirus	33106	NC_003313.1	[S260] [S261] [S262]
**Vibrio phage kappa**	...Peduovirinae; Hpunalikevirus	33134	NC_010275.1	[d.s.]
**Vibrio phage KVP40**	...Tevenvirinae; Schizot4likevirus	244834	NC_005083.2	[S263]
**Vibrio phage vB_VchM-138**	...Myoviridae	44485	NC_019518.1	[S22]
**Vibrio phage vB_VpaM_MAR**	...Myoviridae	41351	NC_019722.1	[S264]
**Vibrio phage vB_VpaS_MAR10**	...Myoviridae	78751	NC_019713.1	[S265]
**Vibrio phage VHML**	...VHML-like phages	43198	NC_004456.1	[S266]
**Vibrio phage VP882**	...Myoviridae	38197	NC_009016.1	[S267]
**Xanthomonas phage OP2**	...Myoviridae	46643	NC_007710.1	[S268]
**Xanthomonas phage vB_XveM_DIBBI**	...Myoviridae	49981	NC_017981.1	[d.s.]
**Yersinia phage L-413C**	...Peduovirinae; P2likeviruses	30728	NC_004745.1	[d.s.]
**Yersinia phage phiR1-37**	...Myoviridae	262391	NC_016163.1	[S269]
**Yersinia phage phiR1-RT**	...Myoviridae	168809	NC_019909.1	[d.s.]

D. References

S1. Lee JH, Shin H, Son B, Ryu S (2012) Complete Genome Sequence of Bacillus cereus Bacteriophage BCP78J Virol 86 (1):637-638

S2. Park J, Yun J, Lim JA, Kang DH, Ryu S (2012) Characterization of an endolysin, LysBPS13, from a Bacillus cereus bacteriophage FEMS Microbiol Lett 332 (1):76-83

S3. Cui Z, Song Z, Wang Y, Zeng L, Shen W, Wang Z, Li Q, He P, Qin J, Guo X (2012) Complete Genome Sequence of Wide-Host-Range Staphylococcus aureus Phage JD007J. Virol. 86 (24):13880-13881

S4. Kilcher S, Loessner MJ, Klumpp J (2010) Brochothrix thermosphacta bacteriophages feature heterogeneous and highly mosaic genomes and utilize unique prophage insertion sitesJ. Bacteriol. 192 (20):5441-5453

S5. Uchiyama J, Rashel M, Takemura I, Wakiguchi H, Matsuzaki S (2008) In silico and in vivo evaluation of bacteriophage phiEF24C, a candidate for treatment of Enterococcus faecalis infectionsAppl. Environ. Microbiol. 74 (13):4149-4163

S6. Chibani-Chennoufi S, Dillmann ML, Marvin-Guy L, Rami-Shojaei S, Br (2004) Lactobacillus plantarum Bacteriophage LP65: a New Member of the SPO1-Like Genus of the Family MyoviridaeJ. Bacteriol. 186 (21):7069-7083

S7. Klumpp J, Dorscht J, Lurz R, Bielmann R, Wieland M, Zimmer M, Calendar R, Loessner MJ (2008) The terminally redundant, nonpermuted genome of Listeria bacteriophage A511: a model for the SPO1-like myoviruses of gram-positive bacteriaJ. Bacteriol. 190 (17):5753-5765

S8. Carlton RM, Noordman WH, Biswas B, de Meester ED, Loessner MJ (2005) Bacteriophage P100 for control of Listeria monocytogenes in foods: genome sequence, bioinformatic analyses, oral toxicity study, and applicationRegul. Toxicol. Pharmacol. 43 (3):301-312

S9. Lobocka M, Hejnowicz MS, Dabrowski K, Gozdek A, Kosakowski J, Witkowska M, Ulatowska MI, Weber-Dabrowska B, Kwiatek M, Parasion S, Gawor J, Kosowska H, Glowacka A (2012) Genomics of Staphylococcal Twort-like Phages - Potential Therapeutics of the Post-Antibiotic EraAdv. Virus Res. 83:143-216

S10. Kwan T, Liu J, DuBow M, Gros P, Pelletier J (2005) The complete genomes and proteomes of 27 Staphylococcus aureus bacteriophagesProc. Natl. Acad. Sci. U.S.A. 102 (14):5174-5179

S11. Gu J, Liu X, Lu R, Li Y, Song J, Lei L, Sun C, Feng X, Du C, Yu H, Yang Y, Han W (2012) Complete Genome Sequence of Staphylococcus aureus Bacteriophage GH15J. Virol. 86 (16):8914-8915

S12. Vandersteegen K, Mattheus W, Ceyssens PJ, Bilocq F, De Vos D, Pirnay JP, Noben JP, Merabishvili M, Lipinska U, Hermans K, Lavigne R (2011) Microbiological and Molecular Assessment of Bacteriophage ISP for the Control of Staphylococcus aureusPLoS ONE 6 (9):E24418

S13. O'Flaherty S, Coffey A, Edwards R, Meaney W, Fitzgerald GF, Ross RP (2004) Genome of staphylococcal phage K: a new lineage of Myoviridae infecting gram-positive bacteria with a low G+C contentJ. Bacteriol. 186 (9):2862-2871

S14. Leila K, Nana B, Tamila M, Ekaterine T, Natia S, Tamila P, Revaz A, Temur T, Elizabeth K, Christine R, Mzia K (2011) Evaluation of lytic activity of staphylococcal bacteriophage Sb-1 against freshly isolated clinical pathogensMicrob Biotechnol. 4(5):643-50

S15. Petrov VM, Ratnayaka S, Nolan JM, Miller ES, Karam JD (2010) Genomes of the T4-related bacteriophages as windows on microbial genome evolution Virol. J. 7 (1):292

S16. Jin J, Li ZJ, Wang SW, Wang SM, Huang DH, Li YH, Ma YY, Wang J, Liu F, Chen XD, Li GX, Wang XT, Wang ZQ, Zhao GQ (2012) Isolation and characterization of ZZ1, a novel lytic phage that infects Acinetobacter baumannii clinical isolates BMC Microbiol. 12 (1):156

S17. Petrov VM, Nolan JM, Bertrand C, Levy D, Desplats C, Krisch HM, Karam JD (2006) Plasticity of the gene functions for DNA replication in the T4-like phages J. Mol. Biol. 361 (1):46-68

S18. Tetart F, Desplats C, Kutateladze M, Monod C, Ackermann HW, Krisch HM (2001) Phylogeny of the major head and tail genes of the wide-ranging T4-type bacteriophages J. Bacteriol. 183 (1):358-366

S19. Shen CJ, Liu YJ, Lu CP (2012) Complete Genome Sequence of Aeromonas hydrophila Phage CC2 J. Virol. 86 (19):10900

S20. Kim JH, Son JS, Choi YJ, Choresca CH, Shin SP, Han JE, Jun JW, Park SC (2012) Complete genomic sequence of a T4-like bacteriophage, phiAS4, infecting Aeromonas salmonicida subsp. salmonicida Arch. Virol. 157 (2):391-395

S21. Kim JH, Son JS, Choi YJ, Choresca CH, Jr, Shin SP, Han JE, Jun JW, Park SC (2012) Complete genome sequence and characterization of a broad-host range T4-like bacteriophage phiAS5 infecting Aeromonas salmonicida subsp. salmonicida Vet. Microbiol. 157 (1-2):164-171

S22. Comeau AM, Tremblay D, Moineau S, Rattei T, Kushkina AI, Tovkach FI, Krisch HM, Ackermann HW (2012) Phage morphology recapitulates phylogeny: the comparative genomics of a new group of myoviruses PLoS ONE 7 (7):E40102

S23. Chen C, Kittichotirat W, Si Y, Bumgarner R (2009) Genome sequence of Aggregatibacter actinomycetemcomitans serotype c strain D11S-1 J. Bacteriol. 191 (23):7378-7379

S24. Hardies SC, Thomas JA, Serwer P (2007) Comparative genomics of Bacillus thuringiensis phage 0305phi8-36: defining patterns of descent in a novel ancient phage lineage Virol. J. 4 97

S25. Thomas JA, Hardies SC, Rolando M, Hayes SJ, Lieman K, Carroll CA, Weintraub ST, Serwer P (2007) Complete genomic sequence and mass spectrometric analysis of highly diverse, atypical Bacillus thuringiensis phage 0305phi8-36 Virology 368 (2):405-421

S26. Yee LM, Matsumoto T, Yano K, Matsuoka S, Sadaie Y, Yoshikawa H, Asai K (2011) The Genome of Bacillus subtilis Phage SP10: A Comparative Analysis with Phage SPO1 Biosci. Biotechnol. Biochem. 75 (5):944-952

S27. Ackermann H-W, Krisch HM, Comeau AM (2011) Morphology and genome sequence of phage phi1402: A dwarf myovirus of the predatory bacterium Bdellovibrio bacteriovorus Bacteriophage 1 (3):05-sty

S28. Summer EJ, Gonzalez CF, Bomer M, Carlile T, Embry A, Kucherka AM, Lee J, Mebane L, Morrison WC, Mark L, King MD, LiPuma JJ, Vidaver AK, Young R (2006) Divergence and mosaicism among virulent soil phages of the Burkholderia cepacia complex J. Bacteriol. 188 (1):255-268

S29. Summer EJ, Gonzalez CF, Carlisle T, Mebane LM, Cass AM, Savva CG, LiPuma J, Young R (2004) Burkholderia cenocepacia phage BcepMu and a family of Mu-like phages encoding potential pathogenesis factors J. Mol. Biol. 340 (1):49-65

S30. Lynch KH, Stothard P, Dennis JJ (2010) Genomic analysis and relatedness of P2-like phages of the Burkholderia cepacia complex BMC Genomics 11 (1):599

S31. Goudie AD, Lynch KH, Seed KD, Stothard P, Shrivastava S, Wishart DS, Dennis JJ (2008) Genomic sequence and activity of KS10, a transposable phage of the Burkholderia cepacia complex BMC Genomics 9 615

S32. Hammerl JA, Jackel C, Reetz J, Hertwig S (2012) The Complete Genome Sequence of Bacteriophage CP21 Reveals Modular Shuffling in Campylobacter Group II Phages J. Virol. 86 (16):8896

S33. Kropinski AM, Arutyunov D, Foss M, Cunningham A, Ding W, Singh A, Pavlov AR, Henry M, Evoy S, Kelly J, Szymanski CM (2011) Genome and Proteome of Campylobacter jejuni Bacteriophage NCTC 12673 Appl. Environ. Microbiol. 77 (23):8265-8271

S34. Sakaguchi Y, Hayashi T, Kurokawa K, Nakayama K, Oshima K, Fujinaga Y, Ohnishi M, Ohtsubo E, Hattori M, Oguma K (2005) The genome sequence of Clostridium botulinum type C neurotoxin-converting phage and the molecular mechanisms of unstable lysogeny Proc. Natl. Acad. Sci. U.S.A. 102 (48):17472-17477

S35. Goh S, Ong PF, Song KP, Riley TV, Chang BJ (2007) The complete genome sequence of Clostridium difficile phage {phi}C2 and comparisons to {phi}CD119 and inducible prophages of CD630 Microbiology (Reading, Engl.) 153 (PT 3), 676-685

S36. Mayer MJ, Narbad A, Gasson MJ (2008) Molecular characterization of a Clostridium difficile bacteriophage and its cloned biologically active endolysin J. Bacteriol. 190 (20):6734-6740

S37. Govind R, Fralick JA, Rolfe RD (2006) Genomic Organization and Molecular Characterization of Clostridium difficile Bacteriophage {Phi}CD119 J. Bacteriol. 188 (7):2568-2577

S38. Meessen-Pinard M, Sekulovic O, Fortier LC (2012) Evidence of In Vivo Prophage Induction during Clostridium difficile Infection Appl. Environ. Microbiol. 78 (21):7662-7670

S39. Shin H, Lee JH, Kim Y, Ryu S (2012) Complete Genome Sequence of Cronobacter sakazakii Bacteriophage CR3 J. Virol. 86 (11):6367-6368

S40. Abbasifar R, Kropinski AM, Sabour PM, Ackermann HW, Alanis, Villa A, Abbasifar A, Griffiths MW (2012) Genome Sequence of Cronobacter sakazakii Myovirus vB_CsaM_GAP31 J. Virol. 86 (24):13830-13831

S41. Abbasifar R, Kropinski AM, Sabour PM, Ackermann HW, Lingohr EJ, Griffiths MW (2012) Complete Genome Sequence of Cronobacter sakazakii Bacteriophage vB_CsaM_GAP161 J. Virol. 86 (24):13806-13807

S42. Sabehi G, Shaulov L, Silver DH, Yanai I, Harel A, Lindell D (2012) A novel lineage of myoviruses infecting cyanobacteria is widespread in the oceans Proc. Natl. Acad. Sci. U.S.A. 109 (6):2037-2042

S43. Liu X, Ren H, Liu W, Wen J, Zou L, Liu C (2010) Morphological observation on lytic cycle of bacteriophag Bp7 Dian Zi Xian Wei Xue Bao 29 (2):182-184

S44. Tang F, Li Y, Zhang W, Lu C (2012) Complete Genome Sequence of T4-Like Escherichia coli Bacteriophage HX01 J. Virol. 86 (24):13871

S45. Jiang H, Jiang X, Wang S, Li C, Chen B, An X, Mi Z, Chen J, Tong Y (2011) The complete genome sequence of a novel T4-like bacteriophage, IME08 Arch. Virol. 156 (8):1489-1492

S46. Jiang X, Jiang H, Li C, Wang S, Mi Z, An X, Chen J, Tong Y (2011) Sequence characteristics of T4-like bacteriophage IME08 benome termini revealed by high throughput sequencing Virol. J. 8 194

S47. Denou E, Bruttin A, Barretto C, Ngom-Bru C, Brussow H, Zuber S (2009) T4 phages against Escherichia coli diarrhea: potential and problems Virology 388 (1):21-30

S48. Chibani-Chennoufi S, Canchaya C, Bruttin A, Brussow H (2004) Comparative genomics of the T4-Like Escherichia coli phage JS98: implications for the evolution of T4 phages J. Bacteriol. 186 (24):8276-8286

S49. Zuber S, Ngom-Bru C, Barretto C, Bruttin A, Brussow H, Denou E (2007) Genome analysis of phage JS98 defines a fourth major subgroup of T4-like phages in Escherichia coli J. Bacteriol. 189 (22):8206-8214

S50. Morgan GJ, Hatfull GF, Casjens S, Hendrix RW (2002) Bacteriophage Mu genome sequence: analysis and comparison with Mu-like prophages in Haemophilus, Neisseria and Deinococcus J. Mol. Biol. 317 (3):337-359

S51. Lobocka MB, Rose DJ, Plunkett G, III, Rusin M, Samojedny A, Lehnherr H, Yarmolinsky MB, Blattner FR (2004) Genome of bacteriophage P1 J. Bacteriol. 186 (21):7032-7068

S52. Birkeland NK, Lindquist BH (1986) Coliphage P2 late control gene ogr. DNA sequence and product identification J. Mol. Biol. 188 (3):487-490

S53. Calendar R, Yu S, Myung H, Barreiro V, Odegrip R, Bertani LE, Carlson K, Davenport L, Mosig G, Christie GE, Haggard-Ljungquist E (1998) The lysogenic conversion genes of coliphage P2 have unusually high AT content (in) Syvanen,M. and Kado,C. (Eds.); HORIZONTAL GENE TRANSFER; Chapman and Hall London

S54. Christie GE, Calendar R (1985) Bacteriophage P2 late promoters. II. Comparison of the four late promoter sequences J. Mol. Biol. 181 (3):373-382

S55. Christie GE, Calendar R (1983) Bacteriophage P2 late promoters. Transcription initiation sites for two late mRNAs J. Mol. Biol. 167 (4):773-790

S56. Christie GE, Haggard-Ljungquist E, Feiwell R, Calendar R (1986) Regulation of bacteriophage P2 late-gene expression: the ogr gene Proc. Natl. Acad. Sci. U.S.A. 83 (10):3238-3242

S57. Christie GE, Temple LM, Bartlett BA, Goodwin TS (2002) Programmed translational frameshift in the bacteriophage P2 FETUD tail gene operon J. Bacteriol. 184 (23):6522-6531

S58. Haggard-Liungquist E, Jacobsen E, Rishovd S, Six WS, Nilssen O, Sunshine MG, Lindqvist BH, Kim KJ, Barreiro V, Koonin EV, Calendar R (1995) Bacteriophage P2: genes involved in baseplate assembly Virology 213 (1):109-121

S59. Haggard-Ljungquist E, Barreiro V, Calendar R, Kurnit DM, Cheng H (1989) The P2 phage old gene: sequence, transcription and translational control Gene 85 (1):25-33

S60. Haggard-Ljungquist E, Halling C, Calendar R (1992) DNA sequences of the tail fiber genes of bacteriophage P2: evidence for horizontal transfer of tail fiber genes among unrelated bacteriophages J. Bacteriol. 174 (5):1462-1477

S61. Haggard-Ljungquist E, Kockum K, Bertani LE (1987) DNA sequences of bacteriophage P2 early genes cox and B and their regulatory sites Mol. Gen. Genet. 208 (1-2):52-56

S62. Linderoth NA, Julien B, Flick KE, Calendar R, Christie GE (1994) Molecular cloning and characterization of bacteriophage P2 genes R and S involved in tail completion Virology 200 (2):347-359

S63. Linderoth NA, Ziermann R, Haggard-Ljungquist E, Christie GE, Calendar R (1991) Nucleotide sequence of the DNA packaging and capsid synthesis genes of bacteriophage P2 Nucleic Acids Res. 19 (25):7207-7214

S64. Lindqvist BH (1981) Recombination between satellite phage P4 and its helper P2. I. In vivo and in vitro construction of P4: :P2 hybrid satellite phage Gene 14 (4):231-241

S65. Liu Y, Saha S, Haggard-Ljungquist E (1993) Studies of bacteriophage P2 DNA replication. The DNA sequence of the cis-acting gene A and ori region and construction of a P2 mini-chromosome J. Mol. Biol. 231 (2):361-374

S66. Ljungquist E, Kockum K, Bertani LE (1984) DNA sequences of the repressor gene and operator region of bacteriophage P2 Proc. Natl. Acad. Sci. U.S.A. 81 (13):3988-3992

S67. Murray K, Isaksson-Forsen AG, Challberg M, Englund PT (1977) Symmetrical nucleotide sequences in the recognition sites for the ter function of bacteriophages P2, 299 and 186 J. Mol. Biol. 112 (3):471-489

S68. Murray K, Murray NE (1973) Terminal nucleotide sequences of DNA from temperate coliphages Nature New Biol. 243 (126):134-139

S69. Saha S, Haggard-Ljungquist E, Nordstrom K (1987) The cox protein of bacteriophage P2 inhibits the formation of the repressor protein and autoregulates the early operon EMBO J. 6 (10):3191-3199

S70. Six EW, Sunshine MG, Williams J, Haggard-Ljungquist E, Lindqvist BH (1991) Morphopoietic switch mutations of bacteriophage P2 Virology 182 (1):34-46

S71. Temple LM, Forsburg SL, Calendar R, Christie GE (1991) Nucleotide sequence of the genes encoding the major tail sheath and tail tube proteins of bacteriophage P2 Virology 181 (1):353-358

S72. Yu A, Bertani LE, Haggard-Ljungquist E (1989) Control of prophage integration and excision in bacteriophage P2: nucleotide sequences of the int gene and att sites Gene 80 (1):11-sty

S73. Ziermann R, Bartlett B, Calendar R, Christie GE (1994) Functions involved in bacteriophage P2-induced host cell lysis and identification of a new tail gene J. Bacteriol. 176 (16):4974-4984

S74. Ziermann R, Calendar R (1990) Characterization of the cos sites of bacteriophages P2 and P4 Gene 96 (1):15-wrz

S75. Halling C, Calendar R, Christie GE, Dale EC, Deho G, Finkel S, Flensburg J, Ghisotti D, Kahn ML, Lane KB, Lin CS, Lindqvist BH, Pierson LS, Six EW, Sunshine MG, Ziermann R (1990) DNA sequence of satellite bacteriophage P4 Nucleic Acids Res. 18 (6):1649

S76. Van, Bokkelen GB, Dale EC, Halling C, Calendar R (1991) Mutational analysis of a bacteriophage P4 late promoter J. Bacteriol. 173 (1):37-45

S77. Arbiol C, Comeau AM, Kutateladze M, Adamia R, Krisch HM (2010) Mobile regulatory cassettes mediate modular shuffling in t4-type phage genomes Genome Biol Evol 2010 140-152

S78. Jamalludeen N, Kropinski AM, Johnson RP, Lingohr E, Harel J, Gyles CL (2008) Complete genomic sequence of bacteriophage phiEcoM-GJ1, a novel phage that has myovirus morphology and a podovirus-like RNA polymerase Appl. Environ. Microbiol. 74 (2):516-525

S79. Recktenwald J, Schmidt H (2002) The nucleotide sequence of Shiga toxin (Stx) 2e-encoding phage phiP27 is not related to other Stx phage genomes, but the modular genetic structure is conserved Infect. Immun. 70 (4):1896-1908

S80. Bullas LR, Mostaghimi AR, Arensdorf JJ, Rajadas PT, Zuccarelli AJ (1991) Salmonella phage PSP3, another member of the P2-like phage group Virology 185 (2):918-921

S81. Julien B, Calendar R (1996) Bacteriophage PSP3 and phiR73 activator proteins: analysis of promoter specificities J. Bacteriol. 178 (19):5668-5675

S82. Ang D, Keppel F, Klein G, Richardson A, Georgopoulos C (2000) Genetic analysis of bacteriophage-encoded cochaperonins Annu. Rev. Genet. 34 439-456

S83. Ang D, Richardson A, Mayer MP, Keppel F, Krisch H, Georgopoulos C (2001) Pseudo-T-even bacteriophage RB49 encodes CocO, a cochaperonin for GroEL, which can substitute for Escherichia coli's GroES and Bacteriophage T4's Gp31 J. Biol. Chem. 276 (12):8720-8726

S84. Desplats C, Dez C, Tetart F, Eleaume H, Krisch HM (2002) Snapshot of the genome of the pseudo-T-even bacteriophage RB49 J. Bacteriol. 184 (10):2789-2804

S85. Desplats C, Krisch HM (2003) The diversity and evolution of the T4-type bacteriophages Res. Microbiol. 154 (4):259-267

S86. Monod C, Repoila F, Kutateladze M, Tetart F, Krisch HM (1997) The genome of the pseudo T-even bacteriophages, a diverse group that resembles T4 J. Mol. Biol. 267 (2):237-249

S87. Wang CC, Yeh LS, Karam JD (1995) Modular organization of T4 DNA polymerase. Evidence from phylogenetics J. Biol. Chem. 270 (44):26558-26564

S88. Wang J, Sattar AK, Wang CC, Karam JD, Konigsberg WH, Steitz TA (1997) Crystal structure of a pol alpha family replication DNA polymerase from bacteriophage RB69 Cell 89 (7):1087-1099

S89. Yeh LS, Hsu T, Karam JD (1998) Divergence of a DNA replication gene cluster in the T4-related bacteriophage RB69 J. Bacteriol. 180 (8):2005-2013

S90. Allison GE, Angeles D, Tran-Dinh N, Verma NK (2002) Complete genomic sequence of SfV, a serotype-converting temperate bacteriophage of Shigella flexneri J. Bacteriol. 184 (7):1974-1987

S91. Huan PT, Bastin DA, Whittle BL, Lindberg AA, Verma NK (1997) Molecular characterization of the genes involved in O-antigen modification, attachment, integration and excision in Shigella flexneri bacteriophage SfV Gene 195 (2):217-227

S92. Huan PT, Whittle BL, Bastin DA, Lindberg AA, Verma NK (1997) Shigella flexneri type-specific antigen V: cloning, sequencing and characterization of the glucosyl transferase gene of temperate bacteriophage SfV Gene 195 (2):207-216

S93. Arisaka F, Ishimoto L, Kassavetis G, Kumazaki T, Ishii S (1988) Nucleotide sequence of the tail tube structural gene of bacteriophage T4 J. Virol. 62 (3):882-886

S94. Arisaka F, Nakako T, Takahashi H, Ishii S (1988) Nucleotide sequence of the tail sheath gene of bacteriophage T4 and amino acid sequence of its product J. Virol. 62 (4):1186-1193

S95. Armstrong J, Brown RS, Tsugita A (1983) Primary structure and genetic organization of phage T4 DNA ligase Nucleic Acids Res. 11 (20):7145-7156

S96. Belle A, Landthaler M, Shub DA (2002) Intronless homing: site-specific endonuclease SegF of bacteriophage T4 mediates localized marker exclusion analogous to homing endonucleases of group I introns Genes Dev. 16 (3):351-362

S97. Bouet JY, Woszczyk J, Repoila F, Francois V, Louarn JM, Krisch HM (1994) Direct PCR sequencing of the ndd gene of bacteriophage T4: identification of a product involved in bacterial nucleoid disruption Gene 141 (1):16-wrz

S98. Bova R, Cascino A, Cipollaro M, Gargano S, Grau O, Micheli MR, Santoro M, Scarlato V, Storlazzi A (1995) Bacteriophage T4 gene 28 DNA Seq. 5 (3):199-201

S99. Bova R, Cascino A, Cipollaro M, Grau O, Micheli MR, Santoro M, Storlazzi A, Scarlato V, Gargano S (1990) Bacteriophage T4 gene 27 Nucleic Acids Res. 18 (10):3046

S100. Broida J, Abelson J (1985) Sequence organization and control of transcription in the bacteriophage T4 tRNA region J. Mol. Biol. 185 (3):545-563

S101. Carles-Kinch K, George JW, Kreuzer KN (1997) Bacteriophage T4 UvsW protein is a helicase involved in recombination, repair and the regulation of DNA replication origins EMBO J. 16 (13):4142-4151

S102. Chu FK, Maley GF, Wang AM, Maley F (1987) Localization of the T4 phage ribonucleotide reductase B1 subunit gene and the nucleotide sequence of its upstream and 5' coding regions Gene 57 (1):143-148

S103. Chu FK, Maley GF, West DK, Belfort M, Maley F (1986) Characterization of the intron in the phage T4 thymidylate synthase gene and evidence for its self-excision from the primary transcript Cell 45 (2):157-166

S104. Daegelen P, Brody E (1990) The rIIA gene of bacteriophage T4. I. Its DNA sequence and discovery of a new open reading frame between genes 60 and rIIA Genetics 125 (2):237-248

S105. Efimov VP, Prilipov AG, Mesyanzhinov VV (1990) Nucleotide sequences of bacteriophage T4 genes 6, 7 and 8 Nucleic Acids Res. 18 (17):5313

S106. Franklin JL, Haseltine D, Davenport L, Mosig G (1998) The largest (70 kDa) product of the bacteriophage T4 DNA terminase gene 17 binds to single-stranded DNA segments and digests them towards junctions with double-stranded DNA J. Mol. Biol. 277 (3):541-557

S107. Franklin JL, Mosig G (1996) Expression of the bacteriophage T4 DNA terminase genes 16 and 17 yields multiple proteins Gene 177 (1-2):179-189

S108. Frazier MW, Mosig G (1990) The bacteriophage T4 gene mrh whose product inhibits late T4 gene expression in an Escherichia coli rpoH (sigma 32) mutant Gene 88 (1):14-lip

S109. Fujisawa H, Yonesaki T, Minagawa T (1985) Sequence of the T4 recombination gene, uvsX, and its comparison with that of the recA gene of Escherichia coli Nucleic Acids Res. 13 (20):7473-7481

S110. Gauss P, Gayle M, Winter RB, Gold L (1987) The bacteriophage T4 dexA gene: sequence and analysis of a gene conditionally required for DNA replication Mol. Gen. Genet. 206 (1):24-34

S111. Gram H, Ruger W (1985) Genes 55, alpha gt, 47 and 46 of bacteriophage T4: the genomic organization as deduced by sequence analysis EMBO J. 4 (1):257-264

S112. Gruidl ME, Chen TC, Gargano S, Storlazzi A, Cascino A, Mosig G (1991) Two bacteriophage T4 base plate genes (25 and 26) and the DNA repair gene uvsY belong to spatially and temporally overlapping transcription units Virology 184 (1):359-369

S113. Hacker KJ, Alberts BM (1992) Overexpression, purification, sequence analysis, and characterization of the T4 bacteriophage dda DNA helicase J. Biol. Chem. 267 (29):20674-20681

S114. Hahn S, Ruger W (1989) Organization of the bacteriophage T4 genome between map positions 150.745 and 145.824 Nucleic Acids Res. 17 (16):6729

S115. Hilse D, Koch T, Ruger W (1989) Nucleotide sequence of the alt gene of bacteriophage T4 Nucleic Acids Res. 17 (16):6731

S116. Hinton DM (1989) Altered expression of the bacteriophage T4 gene 41 (primase-helicase) in an Escherichia coli rho mutant J. Biol. Chem. 264 (24):14440-14446

S117. Ho CK, Shuman S (2002) Bacteriophage T4 RNA ligase 2 (gp24.1) exemplifies a family of RNA ligases found in all phylogenetic domains Proc. Natl. Acad. Sci. U.S.A. 99 (20):12709-12714

S118. Hsu T, Wei RX, Dawson M, Karam JD (1987) Identification of two new bacteriophage T4 genes that may have roles in transcription and DNA replication J. Virol. 61 (2):366-374

S119. Huang WM (1986) Nucleotide sequence of a type II DNA topoisomerase gene. Bacteriophage T4 gene 39 Nucleic Acids Res. 14 (19):7751-7765

S120. Huang WM (1986) The 52-protein subunit of T4 DNA topoisomerase is homologous to the gyrA-protein of gyrase Nucleic Acids Res. 14 (18):7379-7390

S121. Huang WM, Ao SZ, Casjens S, Orlandi R, Zeikus R, Weiss R, Winge D, Fang M (1988) A persistent untranslated sequence within bacteriophage T4 DNA topoisomerase gene 60 Science 239 (4843):1005-1012

S122. Ishimoto LK, Ishimoto KS, Cascino A, Cipollaro M, Eiserling FA (1988) The structure of three bacteriophage T4 genes required for tail-tube assembly Virology 164 (1):81-90

S123. Kadyrov FA, Shlyapnikov MG, Kryukov VM (1997) A phage T4 site-specific endonuclease, SegE, is responsible for a non-reciprocal genetic exchange between T-even-related phages FEBS Lett. 415 (1):75-80

S124. Kai T, Selick HE, Yonesaki T (1996) Destabilization of bacteriophage T4 mRNAs by a mutation of gene 61.5 Genetics 144 (1):14-lip

S125. Kaliman AV, Khasanova MA, Kryukov VM, Tanyashin VI, Bayev AA (1990) The nucleotide sequence of the region of bacteriophage T4 inh(lip)-hoc genes Nucleic Acids Res. 18 (14):4277

S126. Keller B, Bickle TA (1986) The nucleotide sequence of gene 21 of bacteriophage T4 coding for the prohead protease Gene 49 (2):245-251

S127. Keller B, Sengstag C, Kellenberger E, Bickle TA (1984) Gene 68, a new bacteriophage T4 gene which codes for the 17K prohead core protein is involved in head size determination J. Mol. Biol. 179 (3):415-430

S128. Kim BC, Kim K, Park EH, Lim CJ (1997) Nucleotide sequence and revised map location of the arn gene from bacteriophage T4 Mol. Cells 7 (5):694-696

S129. Koch T, Lamm N, Ruger W (1989) Sequencing, cloning and overexpression of genes of bacteriophage T4 between map positions 74.325 and 77.184 Nucleic Acids Res. 17 (11):4392

S130. Koch T, Ruger W (1994) The ADP-ribosyltransferases (gpAlt) of bacteriophages T2, T4, and T6: sequencing of the genes and comparison of their products Virology 203 (2):294-298

S131. Krisch HM, Allet B (1982) Nucleotide sequences involved in bacteriophage T4 gene 32 translational self-regulation Proc. Natl. Acad. Sci. U.S.A. 79 (16):4937-4941

S132. Kutter E, Drivdahl R, Rand K (1984) Identification and characterization of the alc gene product of bacteriophage T4 Genetics 108 (2):291-304

S133. Lamm N, Wang Y, Mathews CK, Ruger W (1988) Deoxycytidylate hydroxymethylase gene of bacteriophage T4. Nucleotide sequence determination and over-expression of the gene Eur. J. Biochem. 172 (3):553-563

S134. Lu MJ, Henning U (1989) The immunity (imm) gene of Escherichia coli bacteriophage T4 J. Virol. 63 (8):3472-3478

S135. Macdonald PM, Mosig G (1984) Regulation of a new bacteriophage T4 gene, 69, that spans an origin of DNA replication EMBO J. 3 (12):2863-2871

S136. Maley GF, Duceman BW, Wang AM, Martinez J, Maley F (1990) Cloning, sequence analysis, and expression of the bacteriophage T4 cd gene J. Biol. Chem. 265 (1):47-51

S137. Marusich EI, Mesyanzhinov VV (1989) Nucleotide and deduced amino acid sequences of bacteriophage T4 gene 20 Nucleic Acids Res. 17 (18):7514

S138. Marusich EI, Mesyanzhinov VV (1989) Nucleotide and deduced amino acid sequences of bacteriophage T4 gene 22 Nucleic Acids Res. 17 (21):8865

S139. Midgley CA, Murray NE (1985) T4 polynucleotide kinase; cloning of the gene (pseT) and amplification of its product EMBO J. 4 (10):2695-2703

S140. Miller ES, Kutter E, Mosig G, Arisaka F, Kunisawa T, Ruger W (2003) Bacteriophage T4 genome Microbiol. Mol. Biol. Rev. 67 (1):86-156

S141. Montag D, Degen M, Henning U (1987) Nucleotide sequence of gene t (lysis gene) of the E. coli phage T4 Nucleic Acids Res. 15 (16):6736

S142. Montag D, Riede I, Eschbach ML, Degen M, Henning U (1987) Receptor-recognizing proteins of T-even type bacteriophages. Constant and hypervariable regions and an unusual case of evolution J. Mol. Biol. 196 (1):165-174

S143. Mosig G, Colowick NE, Pietz BC (1998) Several new bacteriophage T4 genes, mapped by sequencing deletion endpoints between genes 56 (dCTPase) and dda (a DNA-dependent ATPase-helicase) modulate transcription Gene 223 (1-2):143-155

S144. Mosig G, Lin GW, Franklin J, Fan WH (1989) Functional relationships and structural determinants of two bacteriophage T4 lysozymes: a soluble (gene e) and a baseplate-associated (gene 5) protein New Biol. 1 (2):171-179

S145. Nivinskas R, Black LW (1988) Cloning, sequence, and expression of the temperature-dependent phage T4 capsid assembly gene 31 Gene 73 (1):251-257

S146. Nivinskas R, Zajanckauskaite A, Raudonikiene A, Viteniene I (1992) The nucleotide sequence between genes 31 and 30 of bacteriophage T4 DNA Seq. 2 (6):405-409

S147. Oliver DB, Crowther RA (1981) DNA sequence of the tail fibre genes 36 and 37 of bacteriophage T4 J. Mol. Biol. 153 (3):545-568

S148. Orsini G, Ouhammouch M, Le, Caer JP, Brody EN (1993) The asiA gene of bacteriophage T4 codes for the anti-sigma 70 protein J. Bacteriol. 175 (1):85-93

S149. Owen JE, Schultz DW, Taylor A, Smith GR (1983) Nucleotide sequence of the lysozyme gene of bacteriophage T4. Analysis of mutations involving repeated sequences J. Mol. Biol. 165 (2):229-S248

S150. Paddison P, Abedon ST, Dressman HK, Gailbreath K, Tracy J, Mosser E, Neitzel J, Guttman B, Kutter E (1998) The roles of the bacteriophage T4 r genes in lysis inhibition and fine-structure genetics: a new perspective Genetics 148 (4):1539-1550

S151. Parker ML, Christensen AC, Boosman A, Stockard J, Young ET, Doermann AH (1984) Nucleotide sequence of bacteriophage T4 gene 23 and the amino acid sequence of its product J. Mol. Biol. 180 (3):399-416

S152. Penner M, Morad I, Snyder L, Kaufmann G (1995) Phage T4-coded Stp: double-edged effector of coupled DNA and tRNA-restriction systems J. Mol. Biol. 249 (5):857-868

S153. Powell D, Franklin J, Arisaka F, Mosig G (1990) Bacteriophage T4 DNA packaging genes 16 and 17 Nucleic Acids Res. 18 (13):4005

S154. Pribnow D, Sigurdson DC, Gold L, Singer BS, Napoli C, Brosius J, Dull TJ, Noller HF (1981) rII cistrons of bacteriophage T4. DNA sequence around the intercistronic divide and positions of genetic landmarks J. Mol. Biol. 149 (3):337-376

S155. Prilipov AG, Selivanov NA, Efimov VP, Marusich EI, Mesyanzhinov VV (1989) Nucleotide sequences of bacteriophage T4 genes 9, 10 and 11 Nucleic Acids Res. 17 (8):3303

S156. Prilipov AG, Selivanov NA, Nikolaeva LI, Mesyanzhinov VV (1988) Nucleotide and deduced amino acid sequence of bacteriophage T4 gene wac Nucleic Acids Res. 16 (21):10361

S157. Purohit S, Mathews CK (1984) Nucleotide sequence reveals overlap between T4 phage genes encoding dihydrofolate reductase and thymidylate synthase J. Biol. Chem. 259 (10):6261-6266

S158. Ramanculov E, Young R (2001) An ancient player unmasked: T4 rI encodes a t-specific antiholin Mol. Microbiol. 41 (3):575-583

S159. Rand KN, Gait MJ (1984) Sequence and cloning of bacteriophage T4 gene 63 encoding RNA ligase and tail fibre attachment activities EMBO J. 3 (2):397-402

S160. Raudonikiene A, Nivinskas R (1992) Gene rIII is the nearest downstream neighbour of bacteriophage T4 gene 31 Gene 114 (1):85-90

S161. Sanson B, Uzan M (1992) Sequence and characterization of the bacteriophage T4 comC alpha gene product, a possible transcription antitermination factor J. Bacteriol. 174 (20):6539-6547

S162. Scarlato V, Storlazzi A, Gargano S, Cascino A (1989) Bacteriophage T4 late gene expression: overlapping promoters direct divergent transcription of the base plate gene cluster Virology 171 (2):475-483

S163. Selick HE, Stormo GD, Dyson RL, Alberts BM (1993) Analysis of five presumptive protein-coding sequences clustered between the primosome genes, 41 and 61, of bacteriophages T4, T2, and T6 J. Virol. 67 (4):2305-2316

S164. Selivanov NA, Prilipov AG, Mesyanzhinov VV (1988) Nucleotide and deduced amino acid sequence of bacteriophage T4 gene 12 Nucleic Acids Res. 16 (5):2334

S165. Selivanov NA, Prilipov AG, Mesyanzhinov VV (1989) Nucleotide sequences of bacteriophage T4 genes 13, 14 and 15 Nucleic Acids Res. 17 (9):3583

S166. Sharma M, Ellis RL, Hinton DM (1992) Identification of a family of bacteriophage T4 genes encoding proteins similar to those present in group I introns of fungi and phage Proc. Natl. Acad. Sci. U.S.A. 89 (14):6658-6662

S167. Shcherbakov V, Granovsky I, Plugina L, Shcherbakova T, Sizova S, Pyatkov K, Shlyapnikov M, Shubina O (2002) Focused genetic recombination of bacteriophage t4 initiated by double-strand breaks Genetics 162 (2):543-556

S168. Sjoberg BM, Hahne S, Mathews CZ, Mathews CK, Rand KN, Gait MJ (1986) The bacteriophage T4 gene for the small subunit of ribonucleotide reductase contains an intron EMBO J. 5 (8):2031-2036

S169. Spicer EK, Konigsberg WH (1983) Organization and Structure of Four T4 Genes Coding for DNA Replication Proteins in) Mathews,C.K., Kutter,E.M., Mosig,G. and Berget,P.B. (Eds.); BACTERIOPHAGE T4: 291-301; American Society for Microbiology, Washington, DC

S170. Spicer EK, Rush J, Fung C, Reha-Krantz LJ, Karam JD, Konigsberg WH (1988) Primary structure of T4 DNA polymerase. Evolutionary relatedness to eucaryotic and other procaryotic DNA polymerases J. Biol. Chem. 263 (16):7478-7486

S171. Stehr M, Schneider G, Aslund F, Holmgren A, Lindqvist Y (2001) Structural basis for the thioredoxin-like activity profile of the glutaredoxin-like NrdH-redoxin from Escherichia coli J. Biol. Chem. 276 (38):35836-35841

S172. Tomaschewski J, Gram H, Crabb JW, Ruger W (1985) T4-induced alpha- and beta-glucosyltransferase: cloning of the genes and a comparison of their products based on sequencing data Nucleic Acids Res. 13 (21):7551-7568

S173. Tomaschewski J, Ruger W (1987) Nucleotide sequence and primary structures of gene products coded for by the T4 genome between map positions 48.266 kb and 39.166 kb Nucleic Acids Res. 15 (8):3632-3633

S174. Trojanowska M, Miller ES, Karam J, Stormo G, Gold L (1984) The bacteriophage T4 regA gene: primary sequence of a translational repressor Nucleic Acids Res. 12 (15):5979-5993

S175. Truncaite L, Zajanckauskaite A, Nivinskas R (2002) Identification of two middle promoters upstream DNA ligase gene 30 of bacteriophage T4 J. Mol. Biol. 317 (2):179-190

S176. Tseng MJ, Hilfinger JM, Walsh A, Greenberg GR (1988) Total sequence, flanking regions, and transcripts of bacteriophage T4 nrdA gene, coding for alpha chain of ribonucleoside diphosphate reductase J. Biol. Chem. 263 (31):16242-16251

S177. Uzan M, Brody E, Favre R (1990) Nucleotide sequence and control of transcription of the bacteriophage T4 motA regulatory gene Mol. Microbiol. 4 (9):1487-1496

S178. Vaiskunaite R, Miller A, Davenport L, Mosig G (1999) Two new early bacteriophage T4 genes, repEA and repEB, that are important for DNA replication initiated from origin E J. Bacteriol. 181 (22):7115-7125

S179. Valerie K, Stevens J, Lynch M, Henderson EE, de, Riel JK (1986) Nucleotide sequence and analysis of the 58.3 to 65.5-kb early region of bacteriophage T4 Nucleic Acids Res. 14 (21):8637-8654

S180. Volker TA, Gafner J, Bickle TA, Showe MK (1982) Gene 67, a new, essential bacteriophage T4 head gene codes for a prehead core component, PIP. I. Genetic mapping and DNA sequence J. Mol. Biol. 161 (4):479-489

S181. Wang FJ, Ripley LS (1998) The spectrum of acridine resistant mutants of bacteriophage T4 reveals cryptic effects of the tsL141 DNA polymerase allele on spontaneous mutagenesis Genetics 148 (4):1655-1665

S182. Xu W, Gauss P, Shen J, Dunn CA, Bessman MJ (2002) The gene e.1 (nudE.1) of T4 bacteriophage designates a new member of the Nudix hydrolase superfamily active on flavin adenine dinucleotide, adenosine 5'-triphospho-5'-adenosine, and ADP-ribose J. Biol. Chem. 277 (26):23181-23185

S183. Young P, Ohman M, Sjoberg BM (1994) Bacteriophage T4 gene 55.9 encodes an activity required for anaerobic ribonucleotide reduction J. Biol. Chem. 269 (45):27815-27818

S184. Zajanckauskaite A, Malys N, Nivinskas R (1997) A rare type of overlapping genes in bacteriophage T4: gene 30.3' is completely embedded within gene 30.3 by one position downstream Gene 194 (2):157-162

S185. Truncaite L, Simoliunas E, Zajanckauskaite A, Kaliniene L, Mankeviciute R, Staniulis J, Klausa V, Meskys R (2012) Bacteriophage vB_EcoM_FV3: a new member of 'rV5-like viruses' Arch. Virol. 157 (12):2431-2435

S186. Kaliniene L, Klausa V, Truncaite L (2010) Low-temperature T4-like coliphages vB_EcoM-VR5, vB_EcoM-VR7 and vB_EcoM-VR20 Arch. Virol. 155 (6):871-880

S187. Kaliniene L, Klausa V, Zajanckauskaite A, Nivinskas R, Truncaite L (2011) Genome of low-temperature T4-related bacteriophage vB_EcoM-VR7 Arch. Virol. 156 (10):1913-1916

S188. Simoliunas E, Kaliniene L, Truncaite L, Klausa V, Zajanckauskaite A, Meskys R (2012) Genome of Klebsiella sp.-Infecting Bacteriophage vB_KleM_RaK2 J. Virol. 86 (9):5406

S189. Lehman SM, Kropinski AM, Castle AJ, Svircev AM (2009) Complete genome of the broad-host-range Erwinia amylovora phage phiEa21-4 and its relationship to Salmonella phage felix O1 Appl. Environ. Microbiol. 75 (7):2139-2147

S190. Muller I, Kube M, Reinhardt R, Jelkmann W, Geider K (2011) Complete Genome Sequences of Three Erwinia amylovora Phages Isolated in North America and a Bacteriophage Induced from an Erwinia tasmaniensis Strain J. Bacteriol. 193 (3):795-796

S191. Born Y, Fieseler L, Marazzi J, Lurz R, Duffy B, Loessner MJ (2011) Novel Virulent and Broad-Host-Range Erwinia amylovora Bacteriophages Reveal a High Degree of Mosaicism and a Relationship to Enterobacteriaceae Phages Appl. Environ. Microbiol. 77 (17):5945-5954

S192. Kropinski AM, Lingohr EJ, Moyles DM, Chibeu A, Mazzocco A, Franklin K, Villegas A, Ahmed R, She YM, Johnson RP (2012) Escherichia coli O157:H7 Typing Phage V7 Is a T4-Like Virus J. Virol. 86 (18):10246

S193. Villegas A, She YM, Kropinski AM, Lingohr EJ, Mazzocco A, Ojha S, Waddell TE, Ackermann HW, Moyles DM, Ahmed R, Johnson RP (2009) The genome and proteome of a virulent Escherichia coli O157:H7 bacteriophage closely resembling Salmonella phage Felix O1 Virol. J. 6 41

S194. Resch G, Kulik EM, Dietrich FS, Meyer J (2004) Complete genomic nucleotide sequence of the temperate bacteriophage Aa Phi 23 of Actinobacillus actinomycetemcomitans J. Bacteriol. 186 (16):5523-5528

S195. Benjamin RC, Fitzmaurice WP, Huang PC, Scocca JJ (1984) Nucleotide sequence of cloned DNA segments of the Haemophilus influenzae bacteriophage HP1c1 Gene 31 (1-3):173-185

S196. Esposito D, Fitzmaurice WP, Benjamin RC, Goodman SD, Waldman AS, Scocca JJ (1996) The complete nucleotide sequence of bacteriophage HP1 DNA Nucleic Acids Res. 24 (12):2360-2368

S197. Esposito D, Scocca JJ (1994) Identification of an HP1 phage protein required for site-specific excision Mol. Microbiol. 13 (4):685-695

S198. Fitzmaurice WP, Waldman AS, Benjamin RC, Huang PC, Scocca JJ (1984) Nucleotide sequence and properties of the cohesive DNA termini from bacteriophage HP1c1 of Haemophilus influenzae Rd Gene 31 (1-3):197-203

S199. Goodman SD, Scocca JJ (1989) Nucleotide sequence and expression of the gene for the site-specific integration protein from bacteriophage HP1 of Haemophilus influenzae J. Bacteriol. 171 (8):4232-4240

S200. Waldman AS, Goodman SD, Scocca JJ (1987) Nucleotide sequences and properties of the sites involved in lysogenic insertion of the bacteriophage HP1c1 genome into the Haemophilus influenzae chromosome J. Bacteriol. 169 (1):238-246

S201. Williams BJ, Golomb M, Phillips T, Brownlee J, Olson MV, Smith AL (2002) Bacteriophage HP2 of Haemophilus influenzae J. Bacteriol. 184 (24):6893-6905

S202. Zehr ES, Tabatabai LB (2011) Detection of a bacteriophage gene encoding a Mu-like portal protein in Haemophilus parasuis reference strains and field isolates by nested polymerase chain reaction J. Vet. Diagn. Invest. 23 (3):538-542

S203. Zehr ES, Tabatabai LB, Bayles DO (2012) Genomic and proteomic characterization of SuMu, a Mu-like bacteriophage infecting Haemophilus parasuis BMC Genomics 13 331

S204. Mobberley JM, Authement RN, Segall AM, Paul JH (2008) The temperate marine phage PhiHAP-1 of Halomonas aquamarina possesses a linear plasmid-like prophage genome J. Virol. 82 (13):6618-6630

S205. Leblanc C, Caumont-Sarcos A, Comeau AM, Krisch HM (2009) Isolation and genomic characterization of the first phage infecting Iodobacteria: phiPLPE, a myovirus having a novel set of features Environ Microbiol Rep 1 (6):499-509

S206. Alemayehu D, Ross RP, O'Sullivan O, Coffey A, Stanton C, Fitzgerald GF, McAuliffe O (2009) Genome of a virulent bacteriophage Lb338-1 that lyses the probiotic Lactobacillus paracasei cheese strain Gene 448 (1):29-39

S207. Highlander SK, Weissenberger S, Alvarez LE, Weinstock GM, Berget PB (2006) Complete nucleotide sequence of a P2 family lysogenic bacteriophage, varphiMhaA1-PHL101, from Mannheimia haemolytica serotype A1 Virology 350 (1):79-89

S208. Yoshida T, Nagasaki K, Takashima Y, Shirai Y, Tomaru Y, Takao Y, Sakamoto S, Hiroishi S, Ogata H (2008) Ma-LMM01 infecting toxic Microcystis aeruginosa illuminates diverse cyanophage genome strategies J. Bacteriol. 190 (5):1762-1772

S209. Pedulla ML, Ford ME, Houtz JM, Karthikeyan T, Wadsworth C, Lewis JA, Jacobs-Sera D, Falbo J, Gross J, Pannunzio NR, Brucker W, Kumar V, Kandasamy J, Keenan L, Bardarov S, Kriakov J, Lawrence JG, Jacobs WR, Jr, Hendrix RW, Hatfull GF (2003) Origins of highly mosaic mycobacteriophage genomes Cell 113 (2):171-182

S210. Hatfull GF, Pedulla ML, Jacobs-Sera D, Cichon PM, Foley A, Ford ME, Gonda RM, Houtz JM, Hryckowian AJ, Kelchner VA, Namburi S, Pajcini KV, Popovich MG, Schleicher DT, Simanek BZ, Smith AL, Zdanowicz GM, Kumar V, Peebles CL, Jacobs WR, Jr, Lawrence JG, Hendrix RW (2006) Exploring the mycobacteriophage metaproteome: phage genomics as an educational platform PLoS Genet. 2 (6):E92

S211. Pope WH, Jacobs-Sera D, Russell DA, Peebles CL, Al-Atrache Z, Alcoser TA, Alexander LM, Alfano MB, Alford ST, Amy NE, Anderson MD, Anderson AG, Ang AA, Ares M, Jr, Barber AJ, Barker LP, Barrett JM, Barshop WD, Bauerle CM, Bayles IM, Belfield KL, Best AA, Borjon A, Jr, Bowman CA, Boyer CA, Bradley KW, Bradley VA, Broadway LN, Budwal K, Busby KN, Campbell IW, Campbell AM, Carey A, Caruso SM, Chew RD, Cockburn CL, Cohen LB, Corajod JM, Cresawn SG, Davis KR, Deng L, Denver DR, Dixon BR, Ekram S, Elgin SC, Engelsen AE, English BE, Erb ML, Estrada C, Filliger LZ, Findley AM, Forbes L, Forsyth MH, Fox TM, Fritz MJ, Garcia R, George ZD, Georges AE, Gissendanner CR, Goff S, Goldstein R, Gordon KC, Green RD, Guerra SL, Guiney-Olsen KR, Guiza BG, Haghighat L, Hagopian GV, Harmon CJ, Harmson JS, Hartzog GA, Harvey SE, He S, He KJ, Healy KE, Higinbotham ER, Hildebrandt EN, Ho JH, Hogan GM, Hohenstein VG, Holz NA, Huang VJ, Hufford EL, Hynes PM, Jackson AS, Jansen EC, Jarvik J, Jasinto PG, Jordan TC, Kasza T, Katelyn MA, Kelsey JS, Kerrigan LA, Khaw D, Kim J, Knutter JZ, Ko CC, Larkin GV, Laroche JR, Latif A, Leuba KD, Leuba SI, Lewis LO, Loesser-Casey KE, Long CA, Lopez AJ, Lowery N, Lu TQ, Mac V, Masters IR, McCloud JJ, McDonough MJ, Medenbach AJ, Menon A, Miller R, Morgan BK, Ng PC, Nguyen E, Nguyen KT, Nguyen ET, Nicholson KM, Parnell LA, Peirce CE, Perz AM, Peterson LJ, Pferdehirt RE, Philip SV, Pogliano K, Pogliano J, Polley T, Puopolo EJ, Rabinowitz HS, Resiss MJ, Rhyan CN, Robinson YM, Rodriguez LL, Rose AC, Rubin JD, Ruby JA, Saha MS, Sandoz JW, Savitskaya J, Schipper DJ, Schnitzler CE, Schott AR, Segal JB, Shaffer CD, Sheldon KE, Shepard EM, Shepardson JW, Shroff MK, Simmons JM, Simms EF, Simpson BM, Sinclair KM, Sjoholm RL, Slette IJ, Spaulding BC, Straub CL, Stukey J, Sughrue T, Tang TY, Tatyana LM, Taylor SB, Taylor BJ, Temple LM, Thompson JV, Tokarz MP, Trapani SE, Troum AP, Tsay J, Tubbs AT, Walton JM, Wang DH, Wang H, Warner JR, Weisser EG, Wendler SC, Weston-Hafer KA, Whelan HM, Williamson KE, Willis AN, Wirtshafter HS, Wong TW, Wu P, Yang Y, Yee BC, Zaidins DA, Zhang B, Zuniga MY, Hendrix RW, Hatfull GF (2011) Expanding the diversity of mycobacteriophages: insights into genome architecture and evolution PLoS ONE 6 (1):E16329

S212. Baranyi U, Klein R, Lubitz W, Kruger DH, Witte A (2000) The archaeal halophilic virus-encoded Dam-like methyltransferase M. phiCh1-I methylates adenine residues and complements dam mutants in the low salt environment of Escherichia coli Mol. Microbiol. 35 (5):1168-1179

S213. Klein R, Baranyi U, Rossler N, Greineder B, Scholz H, Witte A (2002) Natrialba magadii virus phiCh1: first complete nucleotide sequence and functional organization of a virus infecting a haloalkaliphilic archaeon Mol. Microbiol. 45 (3):851-863

S214. Klein R, Greineder B, Baranyi U, Witte A (2000) The structural protein E of the archaeal virus phiCh1: evidence for processing in Natrialba magadii during virus maturation Virology 276 (2):376-387

S215. Campoy S, Aranda J, Alvarez G, Barbe J, Llagostera M (2006) Isolation and sequencing of a temperate transducing phage for Pasteurella multocida Appl. Environ. Microbiol. 72 (5):3154-3160

S216. Sullivan MB, Huang KH, Ignacio-Espinoza JC, Berlin AM, Kelly L, Weigele PR, DeFrancesco AS, Kern SE, Thompson LR, Young S, Yandava C, Fu R, Krastins B, Chase M, Sarracino D, Osburne MS, Henn MR, Chisholm SW (2010) Genomic analysis of oceanic cyanobacterial myoviruses compared with T4-like myoviruses from diverse hosts and environments Environ. Microbiol. 12 (11):3035-3056

S217. Sullivan MB, Coleman ML, Weigele P, Rohwer F, Chisholm SW (2005) Three Prochlorococcus cyanophage genomes: signature features and ecological interpretations PLoS Biol. 3 (5):E144

S218. Ceyssens PJ, Miroshnikov K, Mattheus W, Krylov V, Robben J, Noben JP, Vanderschraeghe S, Sykilinda N, Kropinski AM, Volckaert G, Mesyanzhinov V, Lavigne R (2009) Comparative analysis of the widespread and conserved PB1-like viruses infecting Pseudomonas aeruginosa Environ. Microbiol. 11 (11):2874-2883

S219. Serwer P, Hayes SJ, Zaman S, Lieman K, Rolando M, Hardies SC (2004) Improved isolation of undersampled bacteriophages: finding of distant terminase genes Virology 329 (2):412-424

S220. Thomas JA, Rolando MR, Carroll CA, Shen PS, Belnap DM, Weintraub ST, Serwer P, Hardies SC (2008) Characterization of Pseudomonas chlororaphis myovirus 201varphi2-1 via genomic Ssequencing, mass spectrometry, and electron microscopy Virology 376 (2):330-338

S221. Burkal'tseva MV, Krylov VN, Pleteneva EA, Shaburova OV, Krylov SV, Volkart G, Sykilinda NN, Kurochkina LP, Mesianzhinov VV (2002) [Phenogenetic characterization of a group of giant Phi KZ-like bacteriophages of Pseudomonas aeruginosa] Genetika 38 (11):1470-1479

S222. Hertveldt K, Lavigne R, Pleteneva E, Sernova N, Kurochkina L, Korchevskii R, Robben J, Mesyanzhinov V, Krylov VN, Volckaert G (2005) Genome comparison of Pseudomonas aeruginosa large phages J. Mol. Biol. 354 (3):536-545

S223. Krylov V, Pleteneva E, Bourkaltseva M, Shaburova O, Volckaert G, Sykilinda N, Kurochkina L, Mesyanzhinov V (2003) Myoviridae bacteriophages of Pseudomonas aeruginosa: a long and complex evolutionary pathway Res. Microbiol. 154 (4):269-275

S224. Lavigne R, Sun WD, Volckaert G (2004) PHIRE, a deterministic approach to reveal regulatory elements in bacteriophage genomes Bioinformatics 20 (5):629-635

S225. Kwan T, Liu J, Dubow M, Gros P, Pelletier J (2006) Comparative Genomic Analysis of 18 Pseudomonas aeruginosa Bacteriophages J. Bacteriol. 188 (3):1184-1187

S226. Garbe J, Bunk B, Rohde M, Schobert M (2011) Sequencing and Characterization of Pseudomonas aeruginosa phage JG004 BMC Microbiol. 11 (1):102

S227. Garbe J, Wesche A, Bunk B, Kazmierczak M, Selezska K, Rohde C, Sikorski J, Rohde M, Jahn D, Schobert M (2010) Characterization of JG024, a Pseudomonas aeruginosa PB1- like broad host range phage under simulated infection conditions BMC Microbiol. 10 (1):301

S228. Uchiyama J, Maeda Y, Takemura I, Chess-Williams R, Wakiguchi H, Matsuzaki S (2009) Blood kinetics of four intraperitoneally administered therapeutic candidate bacteriophages in healthy and neutropenic mice Microbiol. Immunol. 53 (5):301-304

S229. Fukuda K, Ishida W, Uchiyama J, Rashel M, Kato S, Morita T, Muraoka A, Sumi T, Matsuzaki S, Daibata M, Fukushima A (2012) Pseudomonas aeruginosa Keratitis in Mice: Effects of Topical Bacteriophage KPP12 Administration PLoS ONE 7 (10)

230. Vanderschaeghe S (2008) Genome and proteome analysis of newly isolated Pseudomonas aeruginosa phages Thesis, Department of Biosystems, K.U.Leuven, Leuven, Belgium

231. Adriaenssens EM, Mattheus W, Cornelissen A, Shaburova O, Krylov VN, Kropinski AM, Lavigne R (2012) Complete genome sequence of the giant pseudomonas phage lu11 J. Virol. 86 (11):6369-S6370

S232. Alemayehu D, Casey PG, McAuliffe O, Guinane CM, Martin JG, Shanahan F, Coffey A, Ross RP, Hill C (2012) Bacteriophages PhiMR299-2 and PhiNH-4 Can Eliminate Pseudomonas aeruginosa in the Murine Lung and on Cystic Fibrosis Lung Airway Cells MBio 3 (2):E00029-12

S233. Cornelissen A, Hardies SC, Shaburova OV, Krylov VN, Mattheus W, Kropinski AM, Lavigne R (2012) Complete Genome Sequence of the Giant Virus OBP and Comparative Genome Analysis of the Diverse phiKZ-Related Phages J. Virol. 86 (3):1844-1852

S234. Debarbieux L, Leduc D, Maura D, Morello E, Criscuolo A, Grossi O, Balloy V, Touqui L (2010) Bacteriophages can treat and prevent Pseudomonas aeruginosa lung infections J. Infect. Dis. 201 (7):1096-1104

S235. Hayashi T, Kamio Y, Hishinuma F, Usami Y, Titani K, Terawaki Y (1989) Pseudomonas aeruginosa cytotoxin: the nucleotide sequence of the gene and the mechanism of activation of the protoxin Mol. Microbiol. 3 (7):861-868

S236. Hayashi T, Matsumoto H, Ohnishi M, Terawaki Y (1993) Molecular analysis of a cytotoxin-converting phage, phi CTX, of Pseudomonas aeruginosa: structure of the attP-cos-ctx region and integration into the serine tRNA gene Mol. Microbiol. 7 (5):657-667

S237. Nakayama K, Kanaya S, Ohnishi M, Terawaki Y, Hayashi T (1999) The complete nucleotide sequence of phi CTX, a cytotoxin-converting phage of Pseudomonas aeruginosa: implications for phage evolution and horizontal gene transfer via bacteriophages Mol. Microbiol. 31 (2):399-419

S238. Briers Y, Volckaert G, Cornelissen A, Lagaert S, Michiels CW, Hertveldt K, Lavigne R (2007) Muralytic activity and modular structure of the endolysins of Pseudomonas aeruginosa bacteriophages phiKZ and EL Mol. Microbiol. 65 (5):1334-1344

S239. Mesyanzhinov VV, Robben J, Grymonprez B, Kostyuchenko VA, Bourkaltseva MV, Sykilinda NN, Krylov VN, Volckaert G (2002) The genome of bacteriophage phiKZ of Pseudomonas aeruginosa J. Mol. Biol. 317 (1):19-sty

S240. Fujiwara A, Kawasaki T, Usami S, Fujie M, Yamada T (2008) Genomic characterization of Ralstonia solanacearum phage phiRSA1 and its related prophage (phiRSX) in strain GMI1000 J. Bacteriol. 190 (1):143-156

S241. Yamada T, Satoh S, Ishikawa H, Fujiwara A, Kawasaki T, Fujie M, Ogata H (2010) A jumbo phage infecting the phytopathogen Ralstonia solanacearum defines a new lineage of the Myoviridae family Virology 398 (1):135-147

S242. Hjorleifsdottir S, Hreggvidsson GO, Fridjonsson OH, Aevarsson A, Kristjansson JK (2000) Bacteriophage rm 378 of a thermophilic host organism Patent: WO 0075335-A 14-DEC-2000; Decode Genetics EHF. (IS)

S243. McClelland M, Sanderson KE, Spieth J, Clifton SW, Latreille P, Courtney L, Porwollik S, Ali J, Dante M, Du F, Hou S, Layman D, Leonard S, Nguyen C, Scott K, Holmes A, Grewal N, Mulvaney E, Ryan E, Sun H, Florea L, Miller W, Stoneking T, Nhan M, Waterston R, Wilson RK (2001) Complete genome sequence of Salmonella enterica serovar Typhimurium LT2 Nature 413 (6858):852-856

S244. Hooton SP, Timms AR, Rowsell J, Wilson R, Connerton IF (2011) Salmonella Typhimurium-specific bacteriophage PhiSH19 and the origins of species specificity in the Vi01-like phage family Virol. J. 8 (1):498

S245. Santos SB, Kropinski AM, Ceyssens PJ, Ackermann HW, Villegas A, Lavigne R, Krylov VN, Carvalho CM, Ferreira EC, Azeredo J (2011) Genomic and Proteomic Characterization of the Broad-Host-Range Salmonella Phage PVP-SE1: Creation of a New Phage Genus J. Virol. 85 (21):11265-11273

S246. Hanna LF, Matthews TD, Dinsdale EA, Hasty D, Edwards RA (2012) Characterization of the ELPhiS Prophage from Salmonella enterica Serovar Enteritidis Strain LK5 Appl. Environ. Microbiol. 78 (6):1785-1793

S247. Park M, Lee JH, Shin H, Kim M, Choi J, Kang DH, Heu S, Ryu S (2012) Characterization and Comparative Genomic Analysis of a Novel Bacteriophage, SFP10, Simultaneously Inhibiting both Salmonella enterica and Escherichia coli O157:H7 Appl. Environ. Microbiol. 78 (1):58-69

S248. Pickard D, Toribio AL, Petty NK, van, Tonder A, Yu L, Goulding D, Barrell B, Rance R, Harris D, Wetter M, Wain J, Choudhary J, Thomson N, Dougan G (2010) A conserved acetyl esterase domain targets diverse bacteriophages to the Vi capsular receptor of Salmonella enterica serovar Typhi J. Bacteriol. 192 (21):5746-5754

S249. Kim KH, Chang HW, Nam YD, Roh SW, Bae JW (2010) Phenotypic characterization and genomic analysis of the Shigella sonnei bacteriophage SP18 J. Microbiol. 48 (2):213-222

S250. Diaz E, Lopez R, Garcia JL (1992) EJ-1, a temperate bacteriophage of Streptococcus pneumoniae with a Myoviridae morphotype J. Bacteriol. 174 (17):5516-5525

S251. Romero P, Lopez R, Garcia E (2004) Genomic organization and molecular analysis of the inducible prophage EJ-1, a mosaic myovirus from an atypical pneumococcus Virology 322 (2):239-252

S252. Dreher TW, Brown N, Bozarth CS, Schwartz AD, Riscoe E, Thrash C, Bennett SE, Tzeng SC, Maier CS (2011) A freshwater cyanophage whose genome indicates close relationships to photosynthetic marine cyanomyophages Environ. Microbiol. 13 (7):1858-1874

S253. Mann NH, Clokie MR, Millard A, Cook A, Wilson WH, Wheatley PJ, Letarov A, Krisch HM (2005) The genome of S-PM2, a 'photosynthetic' T4-type bacteriophage that infects marine Synechococcus strains J. Bacteriol. 187 (9):3188-3200

S254. Millard A, Clokie MR, Shub DA, Mann NH (2004) Genetic organization of the psbAD region in phages infecting marine Synechococcus strains Proc. Natl. Acad. Sci. U.S.A. 101 (30):11007-11012

S255. Millard AD, Zwirglmaier K, Downey MJ, Mann NH, Scanlan DJ (2009) Comparative genomics of marine cyanomyoviruses reveals the widespread occurrence of Synechococcus host genes localized to a hyperplastic region: implications for mechanisms of cyanophage evolution Environ. Microbiol. 11 (9):2370-2387

S256. Weigele PR, Pope WH, Pedulla ML, Houtz JM, Smith AL, Conway JF, King J, Hatfull GF, Lawrence JG, Hendrix RW (2007) Genomic and structural analysis of Syn9, a cyanophage infecting marine Prochlorococcus and Synechococcus Environ. Microbiol. 9 (7):1675-1695

S257. Naryshkina T, Liu J, Florens L, Swanson SK, Pavlov AR, Pavlova NV, Inman R, Minakhin L, Kozyavkin SA, Washburn M, Mushegian A, Severinov K (2006) Thermus thermophilus bacteriophage phiYS40 genome and proteomic characterization of virions J. Mol. Biol. 364 (4):667-677

S258. Tamakoshi M, Murakami A, Sugisawa M, Tsuneizumi K, Takeda S, Saheki T, Izumi T, Akiba T, Mitsuoka K, Toh H, Yamashita A, Arisaka F, Hattori M, Oshima T, Yamagishi A (2011) Genomic and proteomic characterization of the large Myoviridae bacteriophage varphiTMA of the extreme thermophile Thermus thermophilus Bacteriophage 1 (3):152-164

S259. Seed KD, Bodi KL, Kropinski AM, Ackermann HW, Calderwood SB, Qadri F, Camilli A (2011) Evidence of a dominant lineage of Vibrio cholerae-specific lytic bacteriophages shed by cholera patients over a 10-year period in Dhaka, Bangladesh MBio 2 (1):E00334-E00310

S260. Kapfhammer D, Blass J, Evers S, Reidl J (2002) Vibrio cholerae phage K139: complete genome sequence and comparative genomics of related phages J. Bacteriol. 184 (23):6592-6601

S261. Nesper J, Blass J, Fountoulakis M, Reidl J (1999) Characterization of the major control region of Vibrio cholerae bacteriophage K139: immunity, exclusion, and integration J. Bacteriol. 181 (9):2902-2913

S262. Reidl J, Mekalanos JJ (1995) Characterization of Vibrio cholerae bacteriophage K139 and use of a novel mini-transposon to identify a phage-encoded virulence factor Mol. Microbiol. 18 (4):685-701

S263. Miller ES, Heidelberg JF, Eisen JA, Nelson WC, Durkin AS, Ciecko A, Feldblyum TV, White O, Paulsen IT, Nierman WC, Lee J, Szczypinski B, Fraser CM (2003) Complete genome sequence of the broad-host-range vibriophage KVP40: comparative genomics of a T4-related bacteriophage J. Bacteriol. 185 (17):5220-5233

S264. Alanis, Villa A, Kropinski AM, Abbasifar R, Griffiths MW (2012) Complete Genome Sequence of Vibrio parahaemolyticus Bacteriophage vB_VpaM_MAR J. Virol. 86 (23):13138-13139

S265. Alanis, Villa A, Kropinski AM, Abbasifar R, Abbasifar A, Griffiths MW (2012) Genome Sequence of Temperate Vibrio parahaemolyticus Bacteriophage vB_VpaS_MAR10 J. Virol. 86 (24):13851-13852

S266. Oakey HJ, Cullen BR, Owens L (2002) The complete nucleotide sequence of the Vibrio harveyi bacteriophage VHML J. Appl. Microbiol. 93 (6):1089-1098

S267. Lan SF, Huang CH, Chang CH, Liao WC, Lin IH, Jian WN, Wu YG, Chen SY, Wong HC (2009) Characterization of a new plasmid-like prophage in a pandemic Vibrio parahaemolyticus O3:K6 strain Appl. Environ. Microbiol. 75 (9):2659-2667

S268. Inoue Y, Matsuura T, Ohara T, Azegami K (2006) Sequence analysis of the genome of OP2, a lytic bacteriophage of Xanthomonas oryzae pv. oryzae J. Gen. Plant Pathol. 72 104-110

S269. Kiljunen S, Hakala K, Pinta E, Huttunen S, Pluta P, Gador A, Lonnberg H, Skurnik M (2005) Yersiniophage phiR1-37 is a tailed bacteriophage having a 270 kb DNA genome with thymidine replaced by deoxyuridine Microbiology (Reading, Engl.) 151 (PT 12), 4093-4102
