# Supplementary material for: The Discovery of phiAGATE, A Novel Phage Infecting Bacillus pumilus, Leads to New Insights into the Phylogeny of the Subfamily Spounavirinae
Source: PLoS One. 2014 Jan 23;9(1):e86632. doi: 10.1371/journal.pone.0086632 (PMC3900605; doi:10.1371/journal.pone.0086632)
Supplement: Table S3 — Complete list of all CDSs predicted in the phiAGATE genome. Section A shows CDSs with predicted function, section B lists CDSs with no function assigned that are similar to other sequences from non-redundant protein database and section C includes CDSs that don’t share significant similarity with any known sequences (BLASTp e-value of 1e-10 was used as a cut-off). (PDF) [file pone.0086632.s006.pdf]

A. CDSs with predicted function

gp#	Product name	Length	Strand	CDS coordinates	Accession	Similar to	Accession	E Value	InterPro domains	Ontological terms (GO IDs)	EC number
002	putative baseplate protein W	738	-	86784←87521	AGB62787.1	putative baseplate protein [Bacillus phage BCP78]	YP_006908029	7.81e-103	G3DSA:3.10.450.40	0019068, 0005507, 0048038, 0009308,	-
003	putative baseplate J family protein	1047	-	85724←86770	AGB62786.1	baseplate J family protein [Bacillus phage BCP78]	YP_006908028	6.68e-166	PF04865	0019068	-
008	putative intein containing helicase/endonuclease protein	3057	-	76677←79733	AGB62781.1	ORF006 [Staphylococcus phage Twort]	YP_238583	0	PF00271; SM00490; PS51194; PTHR24031; PTHR24031:SF128; G3DSA:3.40.50.300; PF04851; TIGR01443; PS50818; SM00305; G3DSA:2.170.16.10; G3DSA:3.10.28.10; PS50819; SSF55608; PR00379; PS50817; SSF51294; SSF52540	0006260, 0004386, 0003677, 0005524, 0004519, 0016539	-
010	putative transcriptional regulator	1701	-	74660←76360	AGB62779.1	putative transcriptional regulator [Bacillus phage BCP78]	YP_006908022	0	SM00419; PF13545; G3DSA:1.10.10.10; SSF46785	0006355, 0003700, 0005622	-
011	putative helicase	1473	-	73145←74617	AGB62778.1	putative helicase 1 [Bacillus phage B4]	YP_006908462	0	SM00382; PF03796; G3DSA:3.40.50.300; PS51199; SSF52540; PTHR13682	0006260, 0003678, 0005524, 0006260, 0005657	-
012	putative recombination exonuclease	1056	-	72088←73143	AGB62777.1	putative nuclease SbcCD D subunit [Bacillus phage BCP78]	YP_006908019	2.91e-113	PF12850; G3DSA:3.60.21.10; PTHR30337; PTHR30337:SF0; SSF56300	0006310, 0004527, 0006259, 0016787, 0006260, 0006310	-
014	putative recombination related exonuclease	1905	-	69842←71746	AGB62775.1	putative exonuclease [Bacillus phage BCP78]	YP_006908018	0	PF13558; PTHR32114; PTHR32114:SF0; G3DSA:1.10.287.510; PF13476; SSF52540; G3DSA:3.40.50.300	0006310, 0005488, 0004518	-
016	putative DNA primase	1071	-	68145←69215	AGB62773.1	putative DNA primase [Bacillus phage BCP78]	YP_006908016	8.33e-125	SSF56731; SM00400; PF01807; PTHR30313; PTHR30313:SF0; G3DSA:3.90.580.10; SSF57783	0006260, 0003677, 0003887, 0006260, 0008408, 0042575	EC:2.7.7.7
017	putative deoxyuridine 5'-triphosphate nucleotidohydrolase	618	-	67456←68073	AGB62772.1	putative deoxyuridine 5'-triphosphate nucleotidohydrolase [Bacillus phage BCP78]	YP_006908013	1.01e-55	PTHR11241; PTHR11241:SF0; G3DSA:2.70.40.10; PF00692; SSF51283	0055086, 0046080, 0004170, 0006206	EC:3.6.1.23
020	putative resolvase	582	-	66178←66759	AGB62769.1	hypothetical protein BCB4_0220 [Bacillus phage B4]	YP_006908449	6.47e-55	PD927567	0003676, 0090305, 0004518	-
031	putative ribonucleotide reductase class Ib NrdI	372	-	62388←62759	AGB62758.1	ribonucleotide reductase stimulatory protein [Bacillus subtilis subsp. subtilis str. 168]	ZP_03591761	6.52e-45	PF07972; TIGR00333; G3DSA:3.40.50.360; PIRSF005087; SSF52218	0055086, 0005488, 0008152	-
032	putative ribonucleotide reductase of class Ib (aerobic) subunit alpha 1	771	-	61628←62398	AGB62757.1	bsu nrdEB intein containing protein [Bacillus sp. BT1B_CT2]	ZP_07999929	3.53e-151	SSF51998; PF02867; G3DSA:3.20.70.20; PF00317; PF08343; SSF48168; PTHR11573; PTHR11573:SF4	0055086, 0005971, 0006260, 0016539, 0004519, 0009265, 0005524, 0004748, 0055114, 0006144, 0006206, 0009186	EC:1.17.4.1
033	putative HNH endonuclease	528	-	61058←61585	AGB62756.1	HNH endonuclease family protein [Bacillus amyloliquefaciens LL3]	YP_005546010	4.65e-62	PF13392; SM00507; SSF54060	0004518	-
034	putative ribonucleotide reductase of class Ib (aerobic) subunit alpha 2	1155	-	59479←60633	AGB62755.1	ribonucleotide-diphosphate reductase subunit alpha [Bacillus sp. HYC-10]	ZP_11161285	0	G3DSA:3.20.70.20; PS00089; PR01183; PF02867; TIGR02506; SSF51998; PTHR11573; PTHR11573:SF4	0055086, 0005971, 0006260, 0016539, 0004519, 0009265, 0005524, 0004748, 0055114, 0006144, 0006206, 0009186	EC:1.17.4.1
036	putative ribonucleotide reductase of class Ib (aerobic) subunit beta	978	-	58250←59227	AGB62753.1	hypothetical protein BSSC8_22480 [Bacillus subtilis subsp. subtilis str. SC-8]	ZP_12671304	0	PS00368; PF00268; G3DSA:1.10.620.20; PTHR23409; PTHR23409:SF12; TIGR04171; SSF47240; PIRSF000355	0055086, 0005971, 0006260, 0009265, 0046914, 0009186, 0004748, 0055114, 0006144, 0006206	EC:1.17.4.1
042	putative metallo-dependent phosphatase 1	588	-	55575←56162	AGB62747.1	phosphoesterase [Lactobacillus fermentum IFO 3956]	YP_001843367	7.09e-41	G3DSA:3.60.21.10; SSF56300	0016787	-
045	putative DNA-binding protein	309	-	54024←54332	AGB62744.1	putative DNA-binding protein 3 [Bacillus phage B4]	YP_006908437	2.68e-16	-	0006310, 0003697, 0005524, 0006281, 0009432, 0008094	-
046	putative DNA polymerase A.1	2652	-	51284←53935	AGB62743.1	putative DNA polymerase 2 [Bacillus phage BCP78]	YP_006907997	0	G3DSA:1.10.150.20; SM00482; PR00868; G3DSA:3.30.70.370; PF00476; SSF56672; PF01612; SSF53098; G3DSA:3.30.420.10; PTHR10133; PTHR10133:SF20	0003677	-
048	truncated putative DNA polymerase A.2	669	-	49604←50272	AGB62741.1	putative DNA polymerase 2 [Bacillus phage BCP78]	YP_006907997	5e-98	G3DSA:3.30.70.370; PR00868; PTHR10133; G3DSA:1.10.150.20; PF00476; SM00482; SSF56672	0090116, 0003886, 0003677, 0090116, 0006555	-
051	putative DNA recombination and repair protein RecA	1212	-	46377←47588	AGB62738.1	recombination repair protein [Bacillus phage Bcp1]	ACU27400	2.92e-157	PS50163; PR00142; tmhmm; PTHR22942:SF1; PTHR22942; PS50162; PF00154; G3DSA:3.40.50.300; SSF52540	0006260, 0003887, 0006260, 0003677, 0008408, 0042575	EC:2.7.7.7
054	putative holin	312	-	44995←45306	AGB62735.1	putative holin protein [Bacillus phage BCP78]	YP_006907987	3.74e-16	PF04688; tmhmm	-	-
058	putative metallo-dependent phosphatase 2	1341	-	41630←42970	AGB62731.1	gp148 [Listeria phage A511]	YP_001468528	1.25e-119	PF12850; SSF56300; G3DSA:3.60.21.10	0016787	-
075	putative RNA polymerase sigma factor SigF 1	735	-	32128←32862	AGB62714.1	sporulation sigma-G factor SigG [Thermacetogenium phaeum DSM 12270]	YP_006920306	4.38e-21	PF04545; G3DSA:1.10.10.10; SSF88659; PF04542; TIGR02937; G3DSA:1.10.1740.10; SSF88946; PTHR30385; PTHR30385:SF0	0006355, 0009987, 0003677, 0003700, 0016987	-
076	putative RNA polymerase sigma factor SigF 2	681	-	31381←32061	AGB62713.1	putative RNA polymerase sigma-B factor [Anaerococcus hydrogenalis ACS-025-V-Sch4]	ZP_08169712	3.99e-15	PF04545; G3DSA:1.10.10.10; SSF88659; PR00046; PTHR30385; PTHR30385:SF0; PF04542; TIGR02937; G3DSA:1.10.601.10; SSF88946	0006355, 0003677, 0003700, 0016987	-
089	putative peptidase M23	549	-	25348←25896	AGB62700.1	metalloendopeptidase-like membrane protein [Thermoanaerobacter tengcongensis MB4]	NP_623660	2.77e-20	PTHR21666; PTHR21666:SF7; PF01551; G3DSA:2.70.70.10; SSF51261	0016998, 0008933, 0016787, 0008152, 0016798, 0016020, 0000270, 0005576	-
093	putative DNA translocase	2229	-	20992←23220	AGB62696.1	putative DNA translocase stage III sporulation protein [Bacillus phage BCP78]	YP_006907935	0	SM00382; PS50901; G3DSA:3.40.50.300; PF01580; SSF52540; PTHR22683; PTHR22683:SF1	0006260, 0003677, 0003896, 0008270, 0005657, 0005730, 0006269, 0006351	-
100	putative XRE family transcriptional regulator	231	-	18309←18539	AGB62689.1	XRE family transcriptional regulator [Desulfotomaculum acetoxidans DSM 771]	YP_003193213	1.40e-15	PS50943; PF13443; SM00530; G3DSA:1.10.260.40; SSF47413	0006355, 0043565	-
108	putative hexapeptide repeat containing transferase like protein	855	-	12791←13645	AGB62681.1	Polymer-forming cytoskeletal [Haemophilus paraphrohaemolyticus HK411]	ZP_10075299	2.99e-60	G3DSA:2.160.10.10; SSF51161	0016740	-
139	putative poly-gamma-glutamate hydrolase	675	+	146087→146761	AGB62650.1	poly-gamma-glutamate hydrolase [Bacillus phage phiNIT1]	BAC65290	4.36e-54	PF05908	0046872, 0016787, 0016021, 0016020, 0005886	-
140	putative pectin lyase-like protein	1773	+	144213→145985	AGB62649.1	hypothetical protein [Bacillus phage phiNIT1]	BAI49175	0	PF05048; SM00710; PF13229; SSF51126; G3DSA:2.160.20.10	0016829	-
150	putative dihydrofolate reductase	486	+	138583→139068	AGB62639.1	hypothetical protein [Bacillus phage phiNIT1]	BAI49173	8.20e-56	PR00070; PTHR11549; PF00186; PS51330; G3DSA:3.40.430.10; PIRSF000194; SSF53597	0055086, 0004146, 0006545, 0009165, 0055114, 0050661, 0006761, 0046656	EC:1.5.1.3
151	putative adenylate kinase-like protein	636	+	137933→138568	AGB62638.1	putative adenylate kinase-like protein [Bacillus phage BCP78]	YP_006907858	5.33e-48		0016301, 0044237	-
152	putative thymidylate synthase	918	+	136994→137911	AGB62637.1	hypothetical protein [Bacillus phage phiNIT1]	BAI49171	3.65e-131	PR00108; TIGR03284; PF00303; MF_00008; G3DSA:3.30.572.10; PTHR11549; SSF55831	0055086, 0004799, 0006231	EC:2.1.1.45
153	putative LIM-type zinc finger domain containing protein	234	+	136747→136980	AGB62636.1	hypothetical protein BCP78_0021 [Bacillus phage BCP78]	YP_006907856	8.57e-19	PS00478; G3DSA:2.10.110.10	0008270	-
155	putative PcfJ protein	1479	+	134527→136005	AGB62634.1	hypothetical protein BCB4_0013 [Bacillus phage B4]	YP_006908242	2.32e-106	PF14284	-	-
157	putative PhoH family protein	729	+	132710→133438	AGB62632.1	PhoH family protein [Bacillus phage B4]	YP_006908240	3.69e-104	G3DSA:3.40.50.300; PTHR30473; PF02562; SSF52540	0005524	-
158	putative N-acetylmuramoyl-L-alanine amidase	936	+	131586→132521	AGB62631.1	putative N-acetylmuramoyl-L-alanine amidase [Bacillus macauensis ZFHKF-1]	ZP_10322331	3.10e-59	PF12200; G3DSA:3.40.80.10; PF01510; SM00644; SSF55846	0000270, 0008745, 0009253, 0009252	EC:3.5.1.28
162	putative terminase large subunit	1146	+	128452→129597	AGB62627.1	large terminase [Bacillus phage 1102phi1-3]	AAS45252	0	PF05876	0019068	-
163	putative large terminase subunit-like protein 2	306	+	127788→128093	AGB62626.1	terminase large subunit [Bacillus phage BCP78]	YP_006907847	1.46e-45	-	-	-
164	putative large terminase subunit-like protein 1	375	+	127175→127549	AGB62625.1	putative terminase large subunit [Bacillus phage BCU4]	AEW47511	2.29e-64	-	-	-
172	putative lambda repressor-like DNA-binding protein	321	+	123993→124313	AGB62617.1	putative transcriptional regulator 3 [Bacillus phage B4]	YP_006908501	9.22e-23	PS50943; SM00530; G3DSA:1.10.260.40; PF12844; SSF47413	0006355, 0043565	-
178	putative DNA-cytosine methyltransferase	123	-	118749←118871	AGB62611.1	DNA-cytosine methyltransferase, partial [Pelosinus fermentans B3]	ZP_15523797	9.50e-13	PF00145; SSF53335; G3DSA:3.40.50.150	0007049, 0003677, 0005524, 0007049, 0007059, 0016021, 0051301, 0017111	EC:3.6.1.15
180	putative portal protein	1659	-	116669←118327	AGB62609.1	putative portal protein [Bacillus phage BCP78]	YP_006908060	0	PF04860	0019068	-
181	putative prohead protease	792	-	115761←116552	AGB62608.1	putative prohead protease [Bacillus phage BCP78]	YP_006908058	4.52e-103	PF04586	0019068, 0006508, 0008233	-
183	putative major capsid protein	1425	-	113243←114667	AGB62606.1	putative major capsid protein [Bacillus phage BCP78]	YP_006908056	0	tmhmm	0019068, 0033644, 0019012, 0016021, 0016020	-
190	putative tail sheath protein	1713	-	107664←109376	AGB62599.1	putative tail sheath protein [Bacillus phage BCP78]	YP_006908049	0	PF04984	0019068, 0019012	-
191	putative structural protein	426	-	107187←107612	AGB62598.1	hypothetical protein BCP78_0213 [Bacillus phage BCP78]	YP_006908048	3.66e-88	-	0019068, 0019012	-
193	putative cell wall-binding peptidase-related domain protein	609	-	105851←106459	AGB62596.1	3D domain protein [Bacillus phage B4]	YP_006908482	2.10e-53	PF06725; G3DSA:2.40.40.10; PTHR21666; PTHR21666:SF27	0000270, 0009254, 0004553, 0005576, 0005618, 0019867, 0016998	EC:3.2.1.0
197	putative tail lysin 2	3753	-	100226←103978	AGB62592.1	putative endo-beta-N-acetylglucosaminidase [Bacillus phage Bastille]	YP_006907320	0	tmhmm	0000270, 0016787	-
198	putative tail lysin 1	2145	-	97980←100124	AGB62591.1	putative tail lysin 1 [Bacillus phage BCU4]	AEW47700	0	PF00877; PTHR21666; PTHR21666:SF26; G3DSA:3.90.1720.10; SSF54001	0000270, 0009253, 0004040, 0004553, 0009254, 0019867, 0019064, 0019031, 0046789, 0016787, 0005618, 0007047, 0005576	-
199	putative tail fiber protein	2028	-	95937←97964	AGB62590.1	putative tail fiber 2 [Bacillus phage BCU4]	AEW47699	0	PS50835	0019068, 0005515	-
200	putative minor structural protein 1	3756	-	92168←95923	AGB62589.1	putative tail fiber 1 [Bacillus phage BCU4]	AEW47696	0		0019068, 0016798	-
201	putative minor structural protein 2	2403	-	89686←92088	AGB62588.1	putative minor structural protein [Enterococcus phage phiEF24C]	YP_001504141	4.53e-91	PF05895	0019068	-

B. CDSs with no predicted function similar to known sequences

gp#	Product name	Length	Strand	CDS coordinates	Accession	Similar to	Accession	E Value	InterPro domains	Ontological terms (GO IDs)	EC number
001	hypothetical protein	504	-	87521←88024	AGB62788.1	hypothetical protein BCP78_0195 [Bacillus phage BCP78]	YP_006908030	1.50e-51	-	n/a	n/a
004	hypothetical protein	1389	-	84320←85708	AGB62785.1	gp35 [Listeria phage P100]	AAY53338	1.74e-84	-	n/a	n/a
005	hypothetical protein	531	-	83685←84215	AGB62784.1	gp105 [Listeria phage A511]	YP_001468485	6.60e-54	-	n/a	n/a
006	hypothetical protein	3486	-	80185←83670	AGB62783.1	putative adsorption associated tail protein [Bacillus phage BCP78]	YP_006908026	0	-	n/a	n/a
013	hypothetical protein	258	-	71834←72091	AGB62776.1	hypothetical protein SpyM50665 [Streptococcus pyogenes str. Manfredo]	YP_001128234	8.79e-17	-	n/a	n/a
030	hypothetical protein	549	-	62901←63449	AGB62759.1	hypothetical protein BTF1_31497 [Bacillus thuringiensis HD-789]	YP_006613885	1e-36	-	n/a	n/a
038	hypothetical protein	591	-	57046←57636	AGB62751.1	hypothetical protein YBT020_29026 [Bacillus thuringiensis serovar finitimus YBT-020]	YP_005563595	3.27e-57	-	n/a	n/a
039	hypothetical protein	237	-	56820←57056	AGB62750.1	hypothetical protein PelgB_21842 [Paenibacillus elgii B69]	ZP_09077123	2.17e-21	-	n/a	n/a
040	hypothetical protein	264	-	56463←56726	AGB62749.1	hypothetical protein IIK_05685 [Bacillus cereus VD102]	ZP_17584997	2.95e-12	-	n/a	n/a
041	hypothetical protein	276	-	56172←56447	AGB62748.1	hypothetical protein IIK_05685 [Bacillus cereus VD102]	ZP_17584997	1.68e-13	-	n/a	n/a
044	hypothetical protein	756	-	54316←55071	AGB62745.1	hypothetical protein BCP78_0165 [Bacillus phage BCP78]	YP_006908000	5.40e-133	-	n/a	n/a
047	hypothetical protein	531	-	50673←51203	AGB62742.1	hypothetical protein [Staphylococcus phage SA11]	YP_007005563	8.61e-22	-	n/a	n/a
049	hypothetical protein	486	-	49020←49505	AGB62740.1	hypothetical protein BCP78_0161 [Bacillus phage BCP78]	YP_006907996	9.57e-53	-	n/a	n/a
050	hypothetical protein	1251	-	47670←48920	AGB62739.1	hypothetical protein BCP78_0158 [Bacillus phage BCP78]	YP_006907993	8.63e-82	-	n/a	n/a
052	hypothetical protein	357	-	45974←46330	AGB62737.1	hypothetical protein BCP78_0154 [Bacillus phage BCP78]	YP_006907989	1.02e-40	-	n/a	n/a
053	hypothetical protein	633	-	45358←45990	AGB62736.1	putative RNA polymerase sigma factor [Bacillus phage B4]	YP_006908410	5.42e-78	-	n/a	n/a
055	hypothetical protein	366	-	44610←44975	AGB62734.1	hypothetical protein BCP78_0151 [Bacillus phage BCP78]	YP_006907986	1.11e-11	-	n/a	n/a
056	hypothetical protein	948	-	43314←44261	AGB62733.1	hypothetical protein BCP78_0150 [Bacillus phage BCP78]	YP_006907985	1.51e-89	-	n/a	n/a
062	hypothetical protein	615	-	40358←40972	AGB62727.1	hypothetical protein BCP78_0134 [Bacillus phage BCP78]	YP_006907969	3.24e-49	-	n/a	n/a
067	hypothetical protein	723	-	38188←38910	AGB62722.1	hypothetical protein BCP78_0126 [Bacillus phage BCP78]	YP_006907961	1.37e-119	-	n/a	n/a
068	hypothetical protein	555	-	37529←38083	AGB62721.1	hypothetical protein BCP78_0125 [Bacillus phage BCP78]	YP_006907960	1.71e-44	-	n/a	n/a
069	hypothetical protein	531	-	36891←37421	AGB62720.1	hypothetical protein BCP78_0124 [Bacillus phage BCP78]	YP_006907959	2.24e-49	-	n/a	n/a
070	hypothetical protein	855	-	36037←36891	AGB62719.1	hypothetical protein BCP78_0122 [Bacillus phage BCP78]	YP_006907957	5.34e-28	-	n/a	n/a
071	hypothetical protein	669	-	35272←35940	AGB62718.1	hypothetical protein BCP78_0118 [Bacillus phage BCP78]	YP_006907953	2.57e-11	-	n/a	n/a
073	hypothetical protein	636	-	33787←34422	AGB62716.1	hypothetical protein BCP78_0112 [Bacillus phage BCP78]	YP_006907947	1.03e-21	-	n/a	n/a
074	hypothetical protein	432	-	32989←33420	AGB62715.1	phage protein [Paenibacillus elgii B69]	ZP_09077071	9.57e-18	-	n/a	n/a
087	hypothetical protein	1041	-	26285←27325	AGB62702.1	Ig-like domain containing protein [Bacillus phage B4]	YP_006908362	4.90e-147	SSF53067	n/a	n/a
088	hypothetical protein	267	-	26029←26295	AGB62701.1	hypothetical protein BCB4_0132 [Bacillus phage B4]	YP_006908361	4.64e-12	-	n/a	n/a
090	hypothetical protein	549	-	24464←25012	AGB62699.1	hypothetical protein BCP78_0102 [Bacillus phage BCP78]	YP_006907937	4.49e-22	tmhmm	n/a	n/a
092	hypothetical protein	1011	-	23340←24350	AGB62697.1	hypothetical protein BCP78_0101 [Bacillus phage BCP78]	YP_006907936	1.63e-114	tmhmm	n/a	n/a
101	hypothetical protein	723	-	17029←17751	AGB62688.1	hypothetical protein BCP78_0121 [Bacillus phage BCP78]	YP_006907956	3.64e-105	-	n/a	n/a
102	hypothetical protein	477	-	16538←17014	AGB62687.1	hypothetical protein BCP78_0120 [Bacillus phage BCP78]	YP_006907955	1.36e-57	-	n/a	n/a
105	hypothetical protein	222	-	15112←15333	AGB62684.1	hypothetical protein BCP78_0047 [Bacillus phage BCP78]	YP_006907882	1.35e-15	-	n/a	n/a
107	hypothetical protein	225	-	13854←14078	AGB62682.1	hypothetical protein BAME_13800 [Bacillus sp. M 2-6]	ZP_10162811	1.81e-20	tmhmm	n/a	n/a
109	hypothetical protein	201	-	12476←12676	AGB62680.1	hypothetical protein EFP_gp176 [Enterococcus phage phiEF24C]	YP_001504285	1.87e-17	-	n/a	n/a
115	hypothetical protein	498	-	9206←9703	AGB62674.1	hypothetical protein [Bacillus phage Bastille]	YP_006907490	1.87e-23	-	n/a	n/a
132	hypothetical protein	270	-	2800←3069	AGB62657.1	hypothetical protein GH15_013 [Staphylococcus phage GH15]	YP_007002136	1.62e-14	-	n/a	n/a
154	hypothetical protein	552	+	136091→136642	AGB62635.1	gp128 [Bacillus phage G]	AEO93390	3.00e-50	-	n/a	n/a
160	hypothetical protein	690	+	130506→131195	AGB62629.1	hypothetical protein [Bacillus phage Bastille]	YP_006907554	3.54e-46	tmhmm	n/a	n/a
161	hypothetical protein	801	+	129719→130519	AGB62628.1	gp75 [Listeria phage A511]	YP_001468455	1.74e-31	-	n/a	n/a
165	hypothetical protein	417	+	126759→127175	AGB62624.1	hypothetical protein BCP78_0011 [Bacillus phage BCP78]	YP_006907846	1.03e-45	-	n/a	n/a
169	hypothetical protein	351	-	124951←125301	AGB62620.1	hypothetical protein BCU4_0222 [Bacillus phage BCU4]	AEW47728	7.57e-22	-	n/a	n/a
170	hypothetical protein	327	-	124598←124924	AGB62619.1	hypothetical protein BCP78_0005 [Bacillus phage BCP78]	YP_006907840	1.82e-28	tmhmm	n/a	n/a
171	hypothetical protein	237	-	124339←124575	AGB62618.1	hypothetical protein BCU4_0220 [Bacillus phage BCU4]	AEW47726	1.08e-14	-	n/a	n/a
173	hypothetical protein	606	+	123220→123825	AGB62616.1	hypothetical protein BCB4_0090 [Bacillus phage B4]	YP_006908319	2e-25	-	n/a	n/a
176	hypothetical protein	522	-	119810←120331	AGB62613.1	hypothetical protein HMPREF1012_01050 [Bacillus sp. BT1B_CT2]	ZP_08000016	1.98e-46	-	n/a	n/a
179	hypothetical protein	264	-	118352←118615	AGB62610.1	hypothetical protein BCP78_0226 [Bacillus phage BCP78]	YP_006908061	2.95e-13	-	n/a	n/a
185	hypothetical protein	873	-	112020←112892	AGB62604.1	hypothetical protein BCB4_0264 [Bacillus phage B4]	YP_006908493	5.09e-145	-	n/a	n/a
186	hypothetical protein	846	-	111161←112006	AGB62603.1	hypothetical protein BCB4_0263 [Bacillus phage B4]	YP_006908492	7.16e-113	-	n/a	n/a
187	hypothetical protein	642	-	110536←111177	AGB62602.1	hypothetical protein BCP78_0217 [Bacillus phage BCP78]	YP_006908052	8.62e-92	-	n/a	n/a
188	hypothetical protein	867	-	109657←110523	AGB62601.1	hypothetical protein BCP78_0216 [Bacillus phage BCP78]	YP_006908051	5.12e-102	-	n/a	n/a
189	hypothetical protein	261	-	109404←109664	AGB62600.1	hypothetical protein BCB4_0260 [Bacillus phage B4]	YP_006908489	6.33e-17	-	n/a	n/a
195	hypothetical protein	408	-	104666←105073	AGB62594.1	hypothetical protein BCB4_0251 [Bacillus phage B4]	YP_006908480	1.25e-43	-	n/a	n/a
196	hypothetical protein	591	-	104029←104619	AGB62593.1	hypothetical protein B5S_0245 [Bacillus phage B5S]	AEW47479	1.61e-62	-	n/a	n/a
204	hypothetical protein	765	-	88024←88788	AGB62585.1	hypothetical protein BCP78_0196 [Bacillus phage BCP78]	YP_006908031	5.64e-93	-	n/a	n/a

C. CDSs sharing no significant similarity to known sequences

gp#	Product name	Length	Strand	CDS coordinates	Accession	Similar to	Accession	E Value	InterPro domains	Ontological terms (GO IDs)	EC number
007	hypothetical protein	174	-	79988 ⟵ 80161	AGB62782.1	-	n/a	n/a	-	n/a	n/a
009	hypothetical protein	210	-	76448 ⟵ 76657	AGB62780.1	-	n/a	n/a	-	n/a	n/a
015	hypothetical protein	627	-	69219 ⟵ 69845	AGB62774.1	-	n/a	n/a	-	n/a	n/a
018	hypothetical protein	321	-	67123 ⟵ 67443	AGB62771.1	-	n/a	n/a	-	n/a	n/a
019	hypothetical protein	366	-	66756 ⟵ 67121	AGB62770.1	-	n/a	n/a	-	n/a	n/a
021	hypothetical protein	147	-	65963 ⟵ 66109	AGB62768.1	-	n/a	n/a	-	n/a	n/a
022	hypothetical protein	159	-	65800 ⟵ 65958	AGB62767.1	-	n/a	n/a	-	n/a	n/a
023	hypothetical protein	273	-	65537 ⟵ 65809	AGB62766.1	-	n/a	n/a	-	n/a	n/a
024	hypothetical protein	258	-	65290 ⟵ 65547	AGB62765.1	-	n/a	n/a	-	n/a	n/a
025	hypothetical protein	348	-	64604 ⟵ 64951	AGB62764.1	-	n/a	n/a	-	n/a	n/a
026	hypothetical protein	174	-	64378 ⟵ 64551	AGB62763.1	-	n/a	n/a	-	n/a	n/a
027	hypothetical protein	315	-	64062 ⟵ 64376	AGB62762.1	-	n/a	n/a	-	n/a	n/a
028	hypothetical protein	312	-	63749 ⟵ 64060	AGB62761.1	-	n/a	n/a	-	n/a	n/a
029	hypothetical protein	288	-	63462 ⟵ 63749	AGB62760.1	-	n/a	n/a	-	n/a	n/a
035	hypothetical protein	168	-	59227 ⟵ 59394	AGB62754.1	-	n/a	n/a	-	n/a	n/a
037	hypothetical protein	447	-	57720 ⟵ 58166	AGB62752.1	-	n/a	n/a	-	n/a	n/a
043	hypothetical protein	381	-	55178 ⟵ 55558	AGB62746.1	-	n/a	n/a	-	n/a	n/a
057	hypothetical protein	282	-	42989 ⟵ 43270	AGB62732.1	-	n/a	n/a	-	n/a	n/a
059	hypothetical protein	381	-	41209 ⟵ 41589	AGB62730.1	-	n/a	n/a	-	n/a	n/a
060	hypothetical protein	447	-	41209 ⟵ 41655	AGB62729.1	-	n/a	n/a	-	n/a	n/a
061	hypothetical protein	132	-	40973 ⟵ 41104	AGB62728.1	-	n/a	n/a	-	n/a	n/a
063	hypothetical protein	162	-	40184 ⟵ 40345	AGB62726.1	-	n/a	n/a	-	n/a	n/a
064	hypothetical protein	312	-	39858 ⟵ 40169	AGB62725.1	-	n/a	n/a	-	n/a	n/a
065	hypothetical protein	447	-	39415 ⟵ 39861	AGB62724.1	-	n/a	n/a	-	n/a	n/a
066	hypothetical protein	420	-	39009 ⟵ 39428	AGB62723.1	-	n/a	n/a	-	n/a	n/a
072	hypothetical protein	678	-	34533 ⟵ 35210	AGB62717.1	-	n/a	n/a	-	n/a	n/a
077	hypothetical protein	276	-	31125 ⟵ 31400	AGB62712.1	-	n/a	n/a	-	n/a	n/a
078	hypothetical protein	264	-	30513 ⟵ 30776	AGB62711.1	-	n/a	n/a	-	n/a	n/a
079	hypothetical protein	384	-	30091 ⟵ 30474	AGB62710.1	-	n/a	n/a	-	n/a	n/a
080	hypothetical protein	402	-	29690 ⟵ 30091	AGB62709.1	-	n/a	n/a	-	n/a	n/a
081	hypothetical protein	186	-	29255 ⟵ 29440	AGB62708.1	-	n/a	n/a	-	n/a	n/a
082	hypothetical protein	252	-	28977 ⟵ 29228	AGB62707.1	-	n/a	n/a	-	n/a	n/a
083	hypothetical protein	153	-	28824 ⟵ 28976	AGB62706.1	-	n/a	n/a	PS51257	n/a	n/a
084	hypothetical protein	273	-	28532 ⟵ 28804	AGB62705.1	-	n/a	n/a	-	n/a	n/a
085	hypothetical protein	441	-	27872 ⟵ 28312	AGB62704.1	-	n/a	n/a	-	n/a	n/a
086	hypothetical protein	402	-	27478 ⟵ 27879	AGB62703.1	-	n/a	n/a	-	n/a	n/a
091	hypothetical protein	126	+	24373 ⟶ 24498	AGB62698.1	-	n/a	n/a	-	n/a	n/a
094	hypothetical protein	249	-	20653 ⟵ 20901	AGB62695.1	-	n/a	n/a	-	n/a	n/a
095	hypothetical protein	207	-	20414 ⟵ 20620	AGB62694.1	-	n/a	n/a	-	n/a	n/a
096	hypothetical protein	198	-	20200 ⟵ 20397	AGB62693.1	-	n/a	n/a	-	n/a	n/a
097	hypothetical protein	279	-	19883 ⟵ 20161	AGB62692.1	-	n/a	n/a	-	n/a	n/a
098	hypothetical protein	1032	-	18850 ⟵ 19881	AGB62691.1	-	n/a	n/a	-	n/a	n/a
099	hypothetical protein	330	-	18560 ⟵ 18889	AGB62690.1	-	n/a	n/a	-	n/a	n/a
103	hypothetical protein	327	-	15971 ⟵ 16297	AGB62686.1	-	n/a	n/a	-	n/a	n/a
104	hypothetical protein	477	-	15399 ⟵ 15875	AGB62685.1	-	n/a	n/a	-	n/a	n/a
106	hypothetical protein	843	-	14133 ⟵ 14975	AGB62683.1	-	n/a	n/a	-	n/a	n/a
110	hypothetical protein	396	-	11921 ⟵ 12316	AGB62679.1	-	n/a	n/a	-	n/a	n/a
111	hypothetical protein	378	-	11541 ⟵ 11918	AGB62678.1	-	n/a	n/a	-	n/a	n/a
112	hypothetical protein	183	-	10717 ⟵ 10899	AGB62677.1	-	n/a	n/a	-	n/a	n/a
113	hypothetical protein	330	-	10289 ⟵ 10618	AGB62676.1	-	n/a	n/a	-	n/a	n/a
114	hypothetical protein	303	-	9977 ⟵ 10279	AGB62675.1	-	n/a	n/a	-	n/a	n/a
116	hypothetical protein	153	-	8727 ⟵ 8879	AGB62673.1	-	n/a	n/a	PS51257	n/a	n/a
117	hypothetical protein	249	-	8458 ⟵ 8706	AGB62672.1	-	n/a	n/a	-	n/a	n/a
118	hypothetical protein	573	-	7788 ⟵ 8360	AGB62671.1	-	n/a	n/a	-	n/a	n/a
119	hypothetical protein	126	-	7586 ⟵ 7711	AGB62670.1	-	n/a	n/a	-	n/a	n/a
120	hypothetical protein	552	-	6971 ⟵ 7522	AGB62669.1	-	n/a	n/a	-	n/a	n/a
121	hypothetical protein	303	-	6391 ⟵ 6693	AGB62668.1	-	n/a	n/a	-	n/a	n/a
122	hypothetical protein	222	-	6088 ⟵ 6309	AGB62667.1	-	n/a	n/a	-	n/a	n/a
123	hypothetical protein	213	-	5732 ⟵ 5944	AGB62666.1	-	n/a	n/a	-	n/a	n/a
124	hypothetical protein	306	-	5413 ⟵ 5718	AGB62665.1	-	n/a	n/a	-	n/a	n/a
125	hypothetical protein	405	-	5007 ⟵ 5411	AGB62664.1	-	n/a	n/a	-	n/a	n/a
126	hypothetical protein	243	-	4687 ⟵ 4929	AGB62663.1	-	n/a	n/a	-	n/a	n/a
127	hypothetical protein	282	-	4687 ⟵ 4968	AGB62662.1	-	n/a	n/a	-	n/a	n/a
128	hypothetical protein	327	-	4331 ⟵ 4657	AGB62661.1	-	n/a	n/a	-	n/a	n/a
129	hypothetical protein	438	-	3855 ⟵ 4292	AGB62660.1	-	n/a	n/a	-	n/a	n/a
130	hypothetical protein	219	-	3606 ⟵ 3824	AGB62659.1	-	n/a	n/a	-	n/a	n/a
131	hypothetical protein	414	-	3146 ⟵ 3559	AGB62658.1	-	n/a	n/a	-	n/a	n/a
133	hypothetical protein	381	-	1952 ⟵ 2332,<br>(149127 ⟵ 149507)	AGB62656.1	-	n/a	n/a	-	n/a	n/a
134	hypothetical protein	210	-	1728 ⟵ 1937,<br>(148903 ⟵ 149112)	AGB62655.1	-	n/a	n/a	-	n/a	n/a
135	hypothetical protein	396	-	1166 ⟵ 1561,<br>(148341 ⟵ 148736)	AHB12550.1	-	n/a	n/a	-	n/a	n/a
136	hypothetical protein	291	-	785 ⟵ 1075,<br>(147960 ⟵ 148250)	AHB12549.1	-	n/a	n/a	-	n/a	n/a
137	hypothetical protein	240	-	553 ⟵ 792,<br>(147728 ⟵ 147967)	AGB62652.1	-	n/a	n/a	-	n/a	n/a
138	hypothetical protein	345	-	118 ⟵ 462,<br>(147293 ⟵ 147637)	AHB12548.1	-	n/a	n/a	-	n/a	n/a
141	hypothetical protein	537	+	143475 ⟶ 144011	AGB62648.1	-	n/a	n/a	-	n/a	n/a
142	hypothetical protein	276	+	143187 ⟶ 143462	AGB62647.1	-	n/a	n/a	-	n/a	n/a
143	hypothetical protein	465	+	142733 ⟶ 143197	AGB62646.1	-	n/a	n/a	-	n/a	n/a
144	hypothetical protein	477	+	142267 ⟶ 142743	AGB62645.1	-	n/a	n/a	-	n/a	n/a
145	hypothetical protein	576	+	141702 ⟶ 142277	AGB62644.1	-	n/a	n/a	-	n/a	n/a
146	hypothetical protein	207	+	141494 ⟶ 141700	AGB62643.1	-	n/a	n/a	TIGR03831	n/a	n/a
147	hypothetical protein	675	+	140716 ⟶ 141390	AGB62642.1	-	n/a	n/a	-	n/a	n/a
148	hypothetical protein	411	+	140202 ⟶ 140612	AGB62641.1	-	n/a	n/a	-	n/a	n/a
149	hypothetical protein	798	+	139085 ⟶ 139882	AGB62640.1	-	n/a	n/a	PF12227	n/a	n/a
156	hypothetical protein	1005	+	133428 ⟶ 134432	AGB62633.1	-	n/a	n/a	-	n/a	n/a
159	hypothetical protein	336	+	131188 ⟶ 131523	AGB62630.1	-	n/a	n/a	-	n/a	n/a
166	hypothetical protein	516	+	125973 ⟶ 126488	AGB62623.1	-	n/a	n/a	-	n/a	n/a
167	hypothetical protein	273	+	125689 ⟶ 125961	AGB62622.1	-	n/a	n/a	-	n/a	n/a
168	hypothetical protein	291	-	125291 ⟵ 125581	AGB62621.1	-	n/a	n/a	-	n/a	n/a
174	hypothetical protein	333	-	121502 ⟵ 121834	AGB62615.1	-	n/a	n/a	-	n/a	n/a
175	hypothetical protein	297	-	120404 ⟵ 120700	AGB62614.1	-	n/a	n/a	-	n/a	n/a
177	hypothetical protein	375	-	118890 ⟵ 119264	AGB62612.1	-	n/a	n/a	-	n/a	n/a
182	hypothetical protein	960	-	114797 ⟵ 115756	AGB62607.1	-	n/a	n/a	-	n/a	n/a
184	hypothetical protein	246	-	112916 ⟵ 113161	AGB62605.1	-	n/a	n/a	-	n/a	n/a
192	hypothetical protein	363	-	106684 ⟵ 107046	AGB62597.1	-	n/a	n/a	-	n/a	n/a
194	hypothetical protein	585	-	105156 ⟵ 105740	AGB62595.1	-	n/a	n/a	-	n/a	n/a
202	hypothetical protein	453	-	89205 ⟵ 89657	AGB62587.1	-	n/a	n/a	-	n/a	n/a
203	hypothetical protein	282	-	88877 ⟵ 89158	AGB62586.1	-	n/a	n/a	-	n/a	n/a
